# Effects of interventions on the psychosocial health and well-being of informal caregivers of people with dementia in low- and middle-income countries (LMICs): a systematic review and meta-analysis

**DOI:** 10.1136/bmjgh-2024-016028

**Published:** 2026-01-08

**Authors:** Stephanie Craig, Lana Cook, Carole Parsons, Olinda Santin, John Busby, Gary Mitchell, Catherine Monaghan, Hien Thi Ho, Tran Nguyen, Gillian Carter

**Affiliations:** 1 School of Nursing and Midwifery, Queen’s University Belfast, Belfast, UK; 2 School of Pharmacy, Queen’s University Belfast, Belfast, UK; 3 Centre for Public Health, Queen’s University Belfast, Belfast, UK; 4 Queen’s University Belfast, Belfast, UK; 5 The University of Adelaide, Adelaide, SA, Australia; 6 University of Medicine and Pharmacy at Ho Chi Minh City, Ho Chi Minh City, Ho Chi Minh, Viet Nam

**Keywords:** Systematic review, Global Health

## Abstract

**Background:**

As the incidence of dementia grows globally, so does the number of individuals providing informal care. The highest care burden will likely be experienced by those living in low-resource settings, where need exceeds current service availability. The aim of this review was to explore the effects of interventions to support informal caregivers of people with dementia in low- and middle-income countries (LMICs) with respect to psychosocial outcome measures.

**Methods:**

For this systematic review and meta-analysis, we searched 17 Global Databases and Resources, 12 Regional Databases and Resources, two Global Registers, six Regional Journals and two Global Dementia Organisations, from inception to July 2025. Randomised controlled trials (RCTs) of non-pharmacological interventions (NPIs) delivered to informal caregivers of adults affected by dementia in LMICs were included. Exclusions were made for studies lacking a focus on dementia or informal caregivers, conducted in non-LMICs, focused on needs/attitudes rather than interventions or were reviews (relevant reviews were hand-searched). The primary outcome was caregiver burden, and secondary outcomes included caregiver distress and depression. Where possible, results were synthesised using meta-analysis, and remaining results were reported in a narrative synthesis. Risk of bias was completed using Cochrane Collaboration’s Risk of Bias V.2.0 tool.

**Findings:**

Of 2369 records screened, 35 papers representing 32 RCTs from 13 LMICs were included, of these 24 contributed to the meta-analysis. NPIs in LMICs have a significant improvement post intervention on caregiver burden (MD 8·7, 95% CI 4·1 to 13·3; I^2^=89·9%), distress (MD 4·3, 95% CI 0·5 to 8·2; I^2^=66·5%) and depression (MD 5·9, 95% CI 1·7 to 10·1; I^2^=67·0%). Interventions were delivered in person (10 studies; MD 6.4, 95% CI 1.4 to 11.5; I² = 88.7%), remotely (two studies; MD 8.0, 95% CI 1.9 to 14.1; I² = 37.5%) or as a hybrid of both (two studies; MD 20.9, 95% CI 5.8 to 36.0; I² = 90.1%), with all showing improvement in caregiver burden except for two studies. Narrative synthesis revealed variation in effects on health and well-being; quality of life; anxiety/distress; depression; stress and self-efficacy and factors potentially influencing implementation.

**Interpretation:**

Overall, NPIs for informal dementia carers are effective in LMICs. Due to the heterogeneity of design and delivery, it is impossible to state an optimal component combination. A pragmatic approach is needed to adapt and implement these interventions culturally and contextually. Limitations include inaccessibility of some databases and journals, difficulty in analysing multicomponent interventions and heterogeneity across studies. Nonetheless, consistency in direction of effect was observed, and sensitivity analyses excluding high-risk-of-bias studies did not alter the overall findings.

**PROSPERO registration number:**

CRD42021283611.

**WHAT IS ALREADY KNOWN ON THIS TOPIC**

- Dementia prevalence is rising globally, with the greatest burden projected in low- and middle-income countries (LMICs).
- Informal caregivers will carry much of this burden, with significant health and equity implications.
- No previous international systematic reviews or meta-analyses have examined non-pharmacological interventions (NPIs) for informal caregivers in LMICs.

**WHAT THIS STUDY ADDS**

- This is the first systematic review, meta-analysis and narrative synthesis of NPIs for informal dementia caregivers in LMICs.
- We identified 32 studies (35 reports), including pooled results from 14 studies (2664 participants) using the Zarit Burden Interview.
- Narrative synthesis across six psychosocial domains highlighted factors influencing intervention delivery, such as intervention type, mode and group versus individual delivery.

**HOW THIS STUDY MIGHT AFFECT RESEARCH, PRACTICE OR POLICY**

- NPIs can improve caregiver outcomes, but evidence on long-term effects and resource needs is limited.
- Tailoring interventions to LMIC contexts, considering resources, training and digital readiness is critical for sustainability.
- Co-designed, culturally appropriate approaches may be more cost-effective than repeated effectiveness trials.

## Introduction

 Dementia is a major cause of disability and dependency among older adults worldwide, with significant physical, psychological, social and economic impact.^1^ The global prevalence of dementia is estimated to increase to over 152 million cases by 2050.^2 3^ Projections for low- and middle-income countries (LMICs) suggest they will carry the greatest burden of dementia cases, with 29.8 million cases in 2015 rising to 107.9 million by 2050.^4^ This represents an increase in LMIC prevalence from 58% to 68%.^5^

Accompanying the rising numbers of people with dementia is that of informal caregivers.^3 6 7^ In LMICs, they provide personal care and support to a partner or family member.^8^ Furthermore, caregivers’ unmet needs are the greatest in LMICs.^3^ The deficit of institutional care for people living with dementia in LMICs lays a large burden on informal caregivers,^9^ impacting their physical, psychological and social well-being, alongside financial repercussions.^10 11^

A scoping review of interventions to support family caregivers across Asia found that knowledge gaps exist, particularly in LMICs.^12^ While there is a dynamic dementia-centred healthcare model in many high-income countries (HICs), the development of such in LMICs has been limited.^9 13^ An emerging evidence base exists in HICs for the effects of non-pharmacological interventions (NPIs) to improve caregiver outcomes.^14 15^ However, the reported success of these interventions does not necessarily guarantee generalisability to LMICs.^9^ In this systematic review and meta-analysis, we aimed to examine the effects of NPIs to support informal caregivers of people with dementia in LMICs, with respect to psychosocial outcome measures such as caregiver burden, distress, anxiety and depression.

## Methods

The review question used for this review is: what are the effects of non-pharmacological supportive interventions on psychosocial outcomes (eg, caregiver burden, distress, anxiety and depression) among informal caregivers of people with dementia in LMICs?

### Search strategy and selection criteria

The reporting of this systematic review and meta-analysis was guided by the Preferred Reporting Items for Systematic Reviews and Meta-Analyses (PRISMA) 2020 Statement.^16^ Study selection was limited to randomised controlled trials (RCTs) published as original research in English-language peer-reviewed journals. Non-randomised studies were excluded due to the possibility of confounding and selection biases. Research with informal caregivers of people affected by dementia (of any type and stage of progression) and living in an LMIC as categorised by the World Bank^17^ at time of the review, was eligible for inclusion. Dyadic research where caregivers were a target of the intervention, in relation to both its delivery and outcome measures, was also included. Studies were excluded if they focused on paid caregivers or if the intervention was delivered only to the person with dementia (online supplemental table 1).

Searches were conducted in April–July 2021 and updated in April–May 2023 and re-updated in June 2025. The search space (table 1) comprised: online global and regional databases and resources, global registries and regional journals; trial registries were also searched for unpublished studies. Reported outcomes were compared with the trial protocol (please note slight deviations from the original protocol: (1) this review does not include the outcomes of knowledge and understanding or coping, instead focusing on psychosocial outcomes only and (2) subgroup analyses by delivery mode and risk of bias were conducted post hoc to explore heterogeneity) to examine whether all prespecified review outcomes had been reported. A subject librarian supported the development of the search strategy and the Cochrane LMIC resources list^18^ was consulted when developing the search strategy. Citations were collected originally using EndNote (V.20·4) reference management software and Covidence in 2025 (covidence.org).

**Table 1:** Measures of effectiveness and instruments used to measure outcomes

Dimension (no. of studies; no. of occasions used)	Name of instrument (no. of studies using the scale)
Burden (n=30 studies, 30 occasions)	Zarit Burden Interview*^1^ (n=17)
	Chinese Zarit Burden Interview (n=1)
	Caregiver Burden Inventory^2^ (n=2)
	Neuropsychiatric Inventory Questionnaire (NPIQ): used to indicate burden level of caregiver^3^ (n=1)
Quality of Life (n=14 studies, 13 occasions)	WHO Quality of Life Measure (WHOQOL-BREF)*^4^ (n=5†)
	Self-rated health – EuroQol-Visual Analog Scale^5^ (n=1)
	The Quality of Life Scale^6^ (n=1)
	Quality of Life questionnaire (SF-36)^7^ (n=1)
	SF-36 Iranian version^8^ (n=1)
	WHOQOL-100^9^ (n=1)
	Satisfaction with Life Scale^10^ (n=1)
	Life Satisfaction Scale^10^ (n=1)
	Carer’s Assessment of Satisfaction Index – Turkish Version^11^ (n=1)
Health and well-being (n=11 studies, 19 occasions)	General Health Questionnaire^12^ (n=2)
	Caregiver Mental Health: The Self Reporting Questionnaire – 20^13^ (n=2)
	The Thai General Well-being Schedule^14^ (n=1)
	Subjective Vitality Scales^15^ (n=1)
	Healthy Life Style Behaviour Scale II^16^ (n=1)
	Resilience Scale for Adults^17^ (n=1)
	Caregiver Resilience Scale^18^ (n=1)
	Connor-Davidson Resilience Scale^19^ (n=1)
	Mindfulness Attention Awareness Scale^20^ (n=1)
	Self-Compassion Scale^21^ (n=1)
	12- item Short Form Health Survey^22^ (n=1)
	General Health – exploratory analysis (n=1)†
	Mini-Mental State Examination (n=1)
	Montreal Cognitive Assessment (n=1)
	Activities of daily living (n=1)
	Pittsburgh Sleep Quality Index (n=1)
	Social Support Rating Scale (n=1)
Anxiety/distress (n=12 studies, 13 occasions)	NPIQ Distress Score*^3^ (n=6)
	Beck Anxiety Inventory^23^ (n=2)
	State Trait Anxiety Inventory^24^ (n=1)
	Arabic version The Taylor Manifest Anxiety Scale^25 26^ (n=1)
	Depression, Anxiety and Stress Scale (DASS)−21 (anxiety)^27^ (n=1)
	Hospital Scale of Anxiety and Depression (HAD) – Anxiety subscale^28^ (n=2)
	HAD – Total (n=1)
Depression (n=13 studies, 13 occasions)	Beck Depression Inventory*^29^ (n=4)
	Center for Epidemiological Studies Depression Scale−10^30^ (n=2)
	Arabic version of the Hamilton Depression Rating Scale^31 32^ (n=1)
	DASS−21 (Depression)^27^ (n=1)
	Patient Health Questionnaire (PHQ 9)^33^ (n=1)
	PHQ-4^34^ (n=2)
	HAD – Depression subscale^28^ (n=2)
	Geriatric Depression Scale (n=1)
Stress (n=6 studies, 7 occasions)	Perceived Stress Scale^35^ (n=1)
	Caregiver Strain Index^36^ (n=1)
	Lipp’s Stress Symptoms Inventory for Adults^37^ (n=1)
	DASS − 21 (Stress)^27^ (n=1)
	Stress symptoms – exploratory analysis (n=1)‡
	General stress – exploratory analysis (n=1)‡
Self-efficacy (n=3 studies, 4 occasions)	Revised Caregiving Self-Efficacy Scale^38^ (n=1)
	Self-Efficacy – Relational, Instrumental, Self-soothing (RIS) Eldercare Self-efficacy Scale^39^ (n=1)
	Pearlin Mastery Scale^43^ (n=1)
	General Self-Efficacy Scale (n=1)
	Sense of Competence in Dementia Care Staff Scale (n=1)

* Included in meta-analysis.

† One study used WHOQOL-BREF (Hong Kong version) with different combination of items.

‡ Exploratory analysis by Tran and collegues 58 of study by Hinton and colleagues 29.

SF-36, 36-item Short Form Health Survey.

Conference abstracts, scoping and systematic reviews and meta-analyses were screened along with the citations and reference lists. Global dementia organisations (Alzheimer’s Disease International and Alzheimer’s Society) were contacted for additional ongoing work. Please see online supplemental material 1) for examples of database search strategies.

Search results were exported where titles and abstracts were screened (LC/SC), and a sample ratified (GC). In the case of disagreement, consensus on articles for full text screening was reached by discussion and by a third researcher (CP). Full texts were divided equally among research team pairs who independently assessed each text against the inclusion and exclusion criteria. Decisions were recorded on a shared spreadsheet, a third reviewer resolving any disagreements.

Protocol was registered at PROSPERO CRD42021283611 (https://www.crd.york.ac.uk/prospero/display_record.php?ID=CRD42021283611).

### Data extraction and risk of bias

Two independent reviewers conducted data extraction which incorporated: location, study duration, participant characteristics (gender, duration of caregiving role, relationship to person with dementia), study intervention (delivery format, effect sizes by format, heterogeneity, outcomes) and comparator characteristics (sample size, mean age, SD) and study outcomes (outcome measures, time points, outcome values and type). Risk of bias supplementary table 2) was completed using Cochrane Collaboration’s Risk of Bias V.2.0 tool by two independent reviewers. In both cases, any discrepancies were resolved by a third reviewer (online supplemental material 3).

### Data synthesis and statistical methods

To pool data for meta-analysis, at least three studies needed to report sufficiently similar data using the same scales for the primary/secondary outcomes. Meta-analyses were conducted using DerSimonian-Laird random-effects models with the statistical heterogeneity evaluated using the I^2^ statistic and interpreted using the thresholds defined by the Cochrane Handbook. Scores were extracted for each outcome in the intervention and placebo arm at follow-up and calculated mean differences. When multiple follow-up scores were reported, we used the one closest to the end of the intervention. Additionally, we performed several subgroup analyses to investigate heterogeneity including delivery mode and risk of bias. Publication bias was considered for each meta-analysis with at least ten studies^19^ through visual inspection of funnel plots and the application of Egger’s tests. Data analysis was performed in STATA (V.16).^20^

Narrative synthesis was completed following guidance for systematic reviews.^21^ This addressed intervention effects within specific outcome dimensions where heterogeneous measurement scales were used and meta-analysis was infeasible. These included the dimensions of health and well-being; quality of life; anxiety/distress; depression; stress and self-efficacy. The narrative synthesis also addressed factors potentially influencing intervention delivery including intervention type, delivery mode and being individual or group based. Any improvements from baseline and follow-up for the intervention group and the significance levels between intervention and control group following implementation were examined.

### Role of the funding source

The funder of the study had no role in study design, data collection, data analysis, data interpretation or writing of the report.

## Results

**Figure 1:**
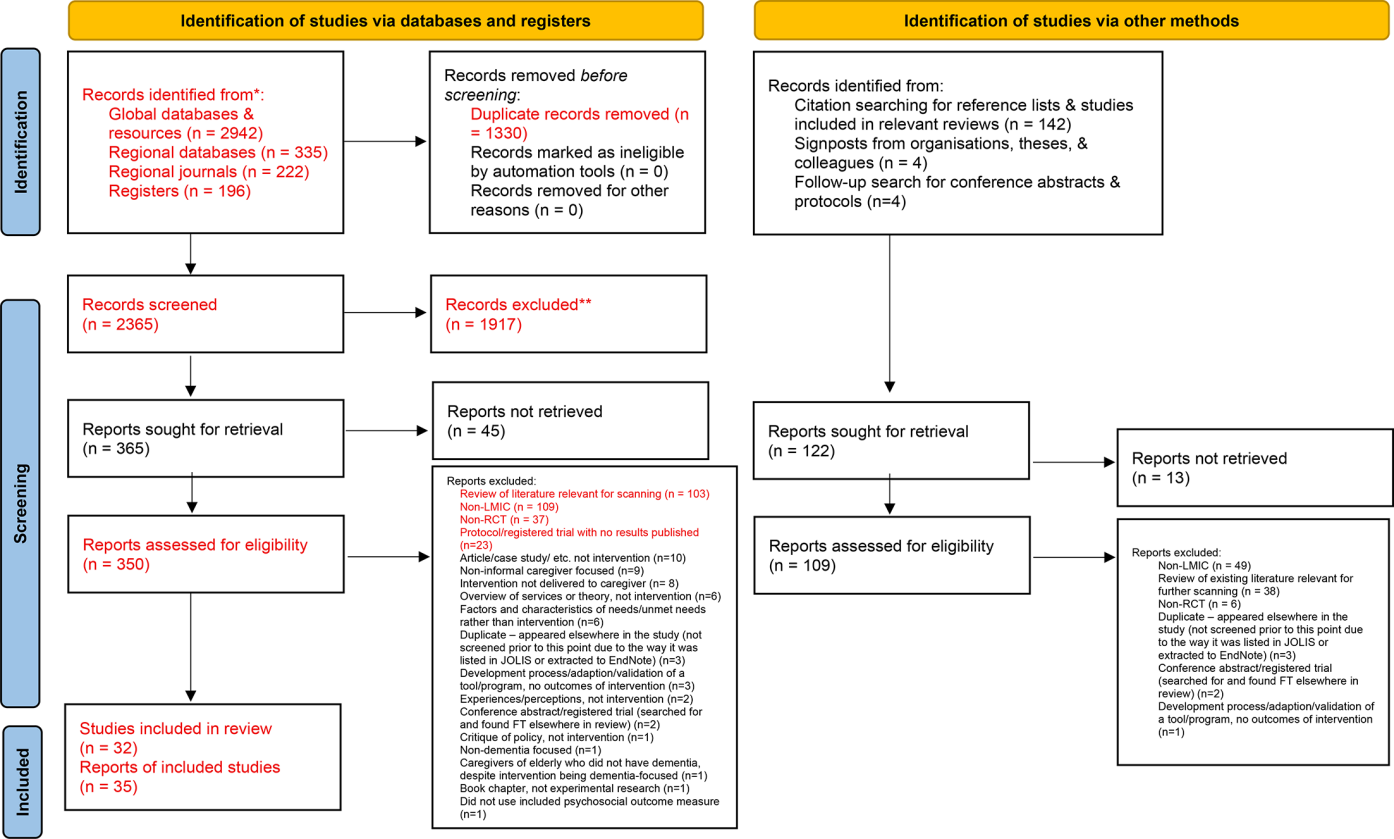
Preferred Reporting Items for Systematic Reviews and Meta-Analyses 2020 flow diagram for new systematic reviews which included searches of databases, registers and other sources. *Consider, if feasible to do so, reporting the number of records identified from each database or register searched (rather than the total number across all databases/registers).**If automation tools were used, indicate how many records were excluded by a human and how many were excluded by automation tools. From: Page MJ, *et al*.^16^ LMIC, low- and middle-income countries; RCT, randomised controlled trial; JOLIS: *Journal of Librarianship and Information Science***.** FT: Full Text

Searches identified 3845 potentially relevant citations. Of these, 1330 were duplicates (figure 1). Following exclusions at title and abstract screening (n=1917), 474 full-text publications were screened (350 from databases and registers and 109 identified via other methods); 137 were reviews. In total, 35 reports of included studies (representing 32 completed studies) were identified as eligible for inclusion, of which 24 reported sufficient data for meta-analysis. Exclusions at each stage were due to an insufficient focus on dementia and/or informal caregivers; non-LMIC; focus on the factors and characteristics of needs/unmet needs or experiences, perceptions and attitudes rather than interventions; or being a review of existing literature (relevant reviews were hand-searched, online supplemental material 4)).

Data were available for 2664 participants across included studies, with individual studies ranging from 21 to 350 participants. Intervention duration ranged from 4 weeks to 5 years. Of the 32 completed studies, eight were conducted in Iran, six in Brazil, four in Turkey, two in India, five in China, three in Vietnam, one in India and Nepal and one each in Colombia, Egypt, Peru, Russia, Malaysia and Thailand.^1^ From the available data, the mean age of caregivers was 54.1 years, and the proportion of female participants was 75.5%.

The assessment tools used in the 32 original studies were organised into seven dimensions of outcome measurements (table 1). The prevalent measure of effects assessed caregiver burden (n=30 studies), examined with the Zarit Burden Interview (ZBI)^22^ in 17 studies.^23^,^41^ ^42^Quality of life was assessed in 12 studies,^2324 26 28 33 34 36 43^,^47^ the prevailing instrument being the WHO Quality of Life Measure (WHOQOL-BREF),^48^ used in five studies.^2433 44^,^46^ Anxiety/distress was assessed in 12 studies;^2427 28 30 33 36 38 40 46 49^,^51^ the Neuropsychiatric Inventory Questionnaire (NPIQ) Distress Scale^52^ was used in six.^24 27 28 33 36 46^ Depression was assessed across 12 studies;^23 26 29 30 34 36 38 40 44 49 51 53 54^ the Beck Depression Inventory^55^ was used in four.^34 36 44 49^ Each of these scales was incorporated into the meta-analysis. The findings of the ZBI measurement are reported, the remainder are provided within online supplemental material 2.

Overall, 13 trials were rated as having a high risk of bias, 19 as some concerns and two as low risk.

### Meta-analysis of the effects of NPIs

Of the original 21 studies assessing the effects of NPIs on caregiver burden, 17 (2011 participants) contributed data to the meta-analysis using the ZBI.^22^ Four studies^23 27 29^ ^42^did not provide adequate data or used a non-compatible variation of the ZBI and were removed from analysis.

**Figure 2:**
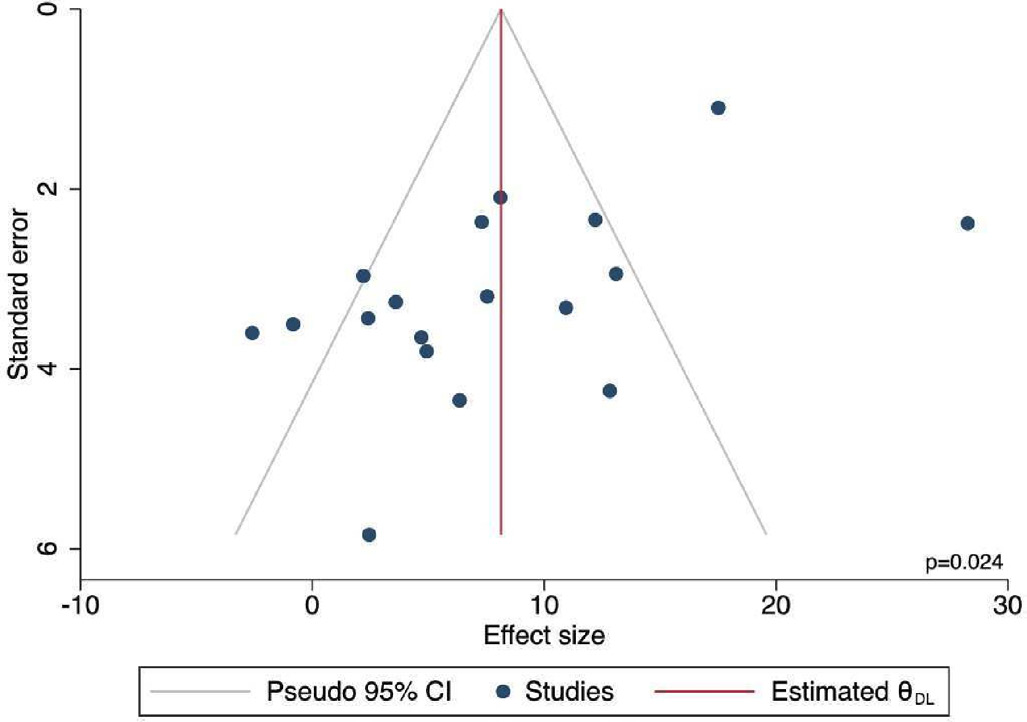
Forest plot for Zarit Burden Interview main results. DL, DerSimonian–Laird.

Results indicated that NPIs in LMICs have a significant improvement on caregiver burden immediately or to first follow-up post intervention (MD 8·1, 95% CI 4·0 to 12·2); although there was substantial heterogeneity between studies (I^2^=89·0%), there was consistency in direction of effects with 15 suggesting an improvement in the ZBI (figure 2). Overall, 11 studies were determined to have low risk of bias or some concern of bias (MD 6.7, 95% CI 1·9 to 11·5; I^2^=86·6%) and high risk of bias for the remaining six (MD 10·5, 95% CI 1·9 to 19·2; I^2^=92·8%), with no evidence of differences between these groups (p=0·236). Sensitivity analysis showed that removing studies at high risk of bias did not impact pooled estimates.

**Figure 3:**
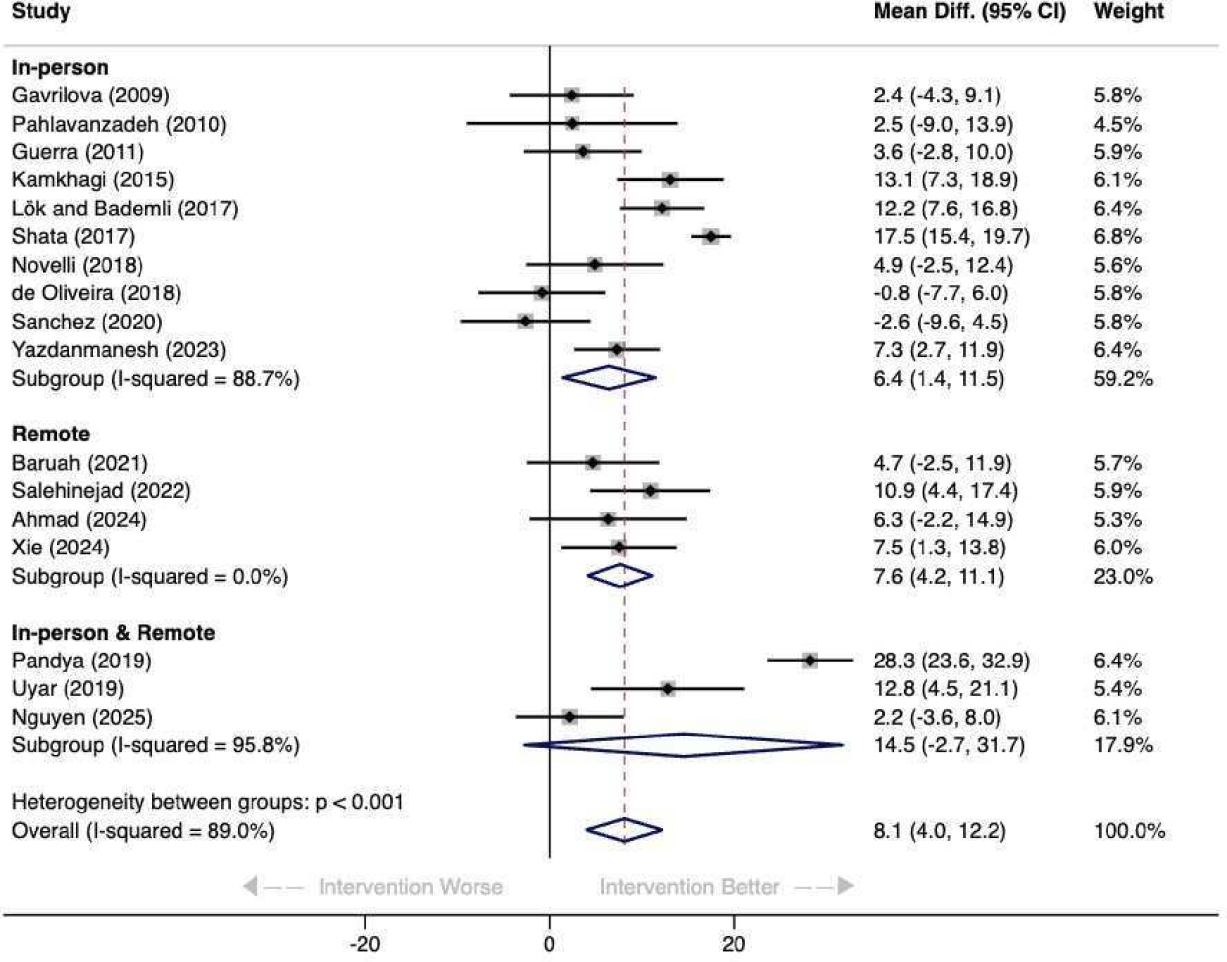
Forest plot of Zarit Burden Interview and intervention delivery modes.

10 studies were delivered in person (MD 6·4, 95% CI 1·4 to 11·5; I^2^=88·7%), four remotely (MD 7.6, 95% CI 4.2 to 11.1; I^2^=0.0%) and three a combination of inperson and remote (MD 14.5, 95% CI −2.7 to 31.7; I^2^=95·8%). All demonstrated improvement in caregiver burden, apart from two.^32 38^ There were significant differences between delivery method subgroups (p<0·001), with overall heterogeneity across all studies high (I² = 89·0%), mostly driven by the large effect of MD 28·3 (95% CI 23·6 to 32·9) reported by Pandya (figure 3).^25^ There was some evidence of potential small study effects (p=0·024) (online supplemental material 2).

### Supplementary and sub-group analysis

We found positive associations for the NPIQ (MD 4·3, 95% CI 0·5 to 8·2; I^2^=66·5%), BDI (MD 5·9, 95% CI 1·7 to 10·1; I^2^=67·0%), but no evidence for WHO Quality of Life BREF subdomains: surrounding environment (MD 2·9, 95% CI −0·1 to 6·0; I^2^=17·5%), physical health (MD 2·0, 95% CI −0·8 to 4·8; I^2^=0·0%), psychological health (MD 2·2, 95% CI −0·4 to 4·7; I^2^=0·0%) and social health (MD 0·8, 95% CI −3·6 to 5·3; I^2^=38·5%). Although there was substantial heterogeneity for NPIQ and BDI, this was not explained by differences in study risk of bias or delivery mode (online supplemental material 2).

### Narrative synthesis

#### Health and well-being

The effects on health and well-being were measured on 19 occasions within 11 studies. In total, seven of these had either low or some concerns of risk of bias^24 27 33 45 56 57^ and the remaining five, a high risk.^25 35 39 54 58^

For low/some concerns studies, outcomes were measured using the General Health Questionnaire (GHQ),^59^ The Thai General Well-being Schedule,^60^ Subjective Vitality Scale,^61^ Self Reporting Questionnaire-20 (SRQ-20) Mental Health,^62^ Connor Davidson Resilience Scale (CD-RISC),^63^ Mindfulness Attention Awareness Scale^64^ and a Self-Compassion Scale.^65^ A significant difference in caregiver well-being between intervention and control groups was reported in three studies.^45 56 57^ Two comprised an educational component, and the third provided a yoga and meditation programme.^45^ No significant difference was reported in other health and well-being measurements (subject vitality and mindfulness) completed in one study.^35^ Two studies measuring SRQ-20 mental health reported a non-significant improvement.^24 33^ Two studies did not provide outcome data for the GHQ^27^ or the CD-RISC.^56^ Of the six studies, all delivered the intervention in person, three individually,^24 27 33^ one as a group^56^ and the remainder via group and individual delivery.^45 57^ An additional study^54^ from China reported significant improvements in caregiver well-being and depression following an 8-week, inperson, multicomponent NPI involving music, reminiscence, touch therapy, mindfulness and cognitive training, delivered in a group format.

Of the four studies judged to have high risk of bias, three^25 35 39^ measured well-being using the Healthy Lifestyle Behaviour Scale,^66^ Resilience Scale for Adults^67^ or Short Form Health Survey (SF-12).^68^ Significant differences were reported for an educational intervention provided individually in person,^35^ a meditation group programme provided in person with individual at-home practice^25^ and an in-person group education intervention.^39^ The fourth study reported an exploratory analysis of general health,^58^ based on data from Hinton and colleagues’ original study^12^; no significant difference was reported.

#### Quality of Life

The impact on caregiver quality of life was measured on 14 occasions within 13 studies. In total, 10 studies had low risk or some concerns of bias,^2426 28 33 36 43^,^47^ and three had high risk.^23 34 54^

In the low/some concerns studies, the outcome measures were: EuroQOL,^69^ Quality of Life Scale,^70^ Quality of Life Questionnaire (SF-36),^71 72^ Life Satisfaction Scale,^73^ Carer’s Assessment of Satisfaction^74^ and WHOQOL-BREF (also used within the meta-analysis).^48^ In all but one of these studies,^45^ the interventions had an educational and training component and three also had a support element.^26 36 46^ Seven reported significant findings in the total scores of scales used^28 44 47^ or in some of the subdomains.^36 43 45 46^ One study of caregivers of older adults with mild cognitive impairment^54^ also reported improved perceived social support following a multicomponent group intervention, although quality of life was not directly measured.

Four of the 10 low/some concerns studies reported non-significant findings^24 26 33 47^ (including a second quality of life scale used by Duru and colleagues).^47^ All had an education/training element in their intervention and two also included a support factor.^26 33^ All were implemented via individual activities, three in person and one online.^26^

In the two high risk of bias studies,^23 34^ one reported a significant improvement in life satisfaction in the intervention group, but non-significant findings when compared with the control.^34^ The other study did not provide data for comparison of intervention to usual care groups.^23^ The interventions in these studies incorporated psychodynamic, psychotherapy or cognitive behavioural therapy (CBT) and were delivered in person as a group session.

#### Anxiety/distress

Anxiety/distress of caregivers was measured on 14 occasions within 13 studies. Nine of these studies had low risk or some concerns of bias,^24 27 28 30 33 36 40 41 46 49 50^ and two were considered high.^38 51^

In the low/some concerns studies, outcome measurements were the Beck Anxiety Inventory,^75^ State Trait Anxiety Inventory,^76^ Taylor Manifest Anxiety Scale^77 78^ and the NPIQ Distress Scale (also assessed in the meta-analysis).^52^ Six of these nine studies,^28 30 36 46 49 50^ (two scales assessed in one study by Uyar and colleagues)^36^ reported a significant improvement in anxiety or distress in the intervention group. Of these studies, one study intervention incorporated a yoga and meditation programme^49^ as both an inperson group and individual activity, four examined an educational and support intervention,^30 36 46 50^ and the remaining study had an educational and training programme.^28^ Of the studies reporting non-significant findings, two revealed a trend towards a reduction in stress in the intervention group.^24 33^ The remaining study^27^ reported a reduction in stress within the intervention, but no information was provided for the control group. These studies were delivered individually in person, two contained an education and support element^27 33^ and the third focused on education and training.^24^ A telephone-delivered psychoeducational intervention in Malaysia,^40^ delivered individually over 12 weeks, demonstrated significant reductions in caregiver anxiety and psychological distress compared with usual care. Similarly, a culturally adapted multicomponent intervention in Vietnam (REACH VN)^41^ significantly reduced psychological distress and perceived stress at 3 months, although most effects were not sustained at 6 months.

In the two high risk of bias studies,^38 51^ both reported no significant impact of the intervention on anxiety measured by Depression, Anxiety and Stress Scale (DASS)−21^79^ or the anxiety subscale of Hospital Scale of Anxiety and Depression (HAD).^80^ A significant difference was found with the total HAD scale score. One study consisted of an education and training programme with a support element, delivered as in-person group sessions,^51^ and the other comprised education and mindfulness, delivered individually in person.

#### Depression

The effect of NPIs on depression experienced by caregivers was measured on 13 occasions within 13 studies. In total, six had low risk or some concerns of risk of bias,^26 30 36 40 44 49^ and seven were considered high.^23 29 34 38 51 53 54^

In the low/some concerns studies, outcomes were assessed using the Beck Depression Inventory (BDI),^55^ (also used in the meta-analysis), Centre for Epidemiological Studies Depression Scale – 10^81^ and Hamilton Depression Rating Scale (Arabic version).^82^ Four of these studies reported a significant improvement in caregivers’ depression levels.^30 36 44 49^ One was a yoga and meditation intervention,^49^ and another was a brief group CBT intervention.^30^ Both delivered their interventions as a group in person. Two focused on education and support intervention components^36 44^ and were delivered in person on an individual basis, with phone call support or in combination with group support.^36^ A telephone-delivered psychoeducational intervention in Malaysia^40^ also reported a significant postintervention reduction in depressive symptoms among caregivers. A multicomponent group programme in China^54^ reported reduced caregiver depression, particularly among those caring for participants with greater cognitive impairment. One study rated at a low risk of bias reported non-significant findings between the intervention (a training and support programme only delivered individually online) and the comparator.^26^

In the six high risk of bias studies, depression was measured via the BDI,^55^ the DASS-21,^79^ Patient Health Questionnaire,^83 84^ (one study measured depression/anxiety but did not separate the results)^29^ and the HAD-depression subscale.^80^ A significant difference was reported in three of these studies,^23 29 53^ in which interventions all incorporated forms of CBT in person (one as a group,^23^ and two individually).^29 53^ The remaining studies reported non-significant findings^34 38 51^; two interventions focused on a psychotherapy element, one delivered in person as a group^34^ and the other individually.^38^ The remaining study focused on inperson education and support.^51^

#### Stress

The effects of NPIs in reducing caregiver stress were measured on seven occasions within six studies, two of which were judged as having some concerns of bias,^41 49 85^ and three had high risk of bias.^23 51 58^

The two studies with some concerns of bias measured stress via Caregiver Strain Index^86^ and Lipp’s Stress Symptoms Inventory for Adults.^87^ They both demonstrated a significant improvement in stress levels, with a yoga and meditation programme intervention^49^ and a spiritual therapy model,^85^ both delivered in person via a group. The REACH VN intervention in Vietnam^41^ was also associated with a significant short-term reduction in perceived stress at 3 months, although this was not maintained at 6 months. Of the remaining studies rated as having a high risk of bias,^23 51^ two measured stress via the Perceived Stress Scale ^88^or DASS-21 (Stress).^79^ They did not report significant findings for in-person group interventions providing CBT^23^ or support and education.^51^ The final study^58^ completed an exploratory analysis of data on stress symptoms and general stress from the original study by Hinton and colleagues^29^; no significant findings are reported.

#### Self-efficacy

The effect of NPIs on caregiver self-efficacy levels was measured on four occasions within three studies, one of which had low risk of bias,^26 89^ and the other a high risk.^25^

The low risk of bias study^26^ used the outcome measures of Relational, Instrumental, Self-soothing (RIS) Eldercare Self-efficacy Scale^90^ and Pearlin Mastery Scale.^91^ No significant differences between intervention and control groups were reported for the individual online skills training and support programme. A low risk of bias study from China^96^ found that a 6-month, internet-based caregiver training programme significantly improved caregiving competence, as measured by the Sense of Competence in Dementia Care Staff Scale. The high risk of bias study^25^ used the Revised Caregiving Self-Efficacy Scale.^92^ The authors demonstrated a significant difference in all domains of self-efficacy due to their meditation programme compared with control. This was delivered in person as a group and individually at home.

## Discussion

To our knowledge, this is the first systematic review, meta-analysis and narrative synthesis to review the effects of NPIs for informal dementia caregivers in LMICs. A total of 35 papers reporting 32 RCT studies, comprising 2664 participants, met the criteria for the review, representing 13 LMICs. The pooled meta-analytic results suggest that NPIs may reduce caregiver burden and symptoms of distress and depression; however, the magnitude of these effects should be interpreted with caution given the wide CIs for some outcomes, variation in study quality and both statistical and clinical heterogeneity. No evidence of benefit was found for quality-of-life subdomains.

Narrative synthesis identified a diverse range of outcomes and measurement tools, with health and well-being being the second most frequently assessed outcome after burden, indicating the recognition of carers’ health needs in both developing and developed nations.^93^ However, health services in LMICs may lack the necessary resources to rapidly develop and meet demand, potentially leaving a significant portion of informal carers without support.^94^

Cost-effectiveness was not measured in any studies; however, existing evidence suggests that psychosocial supportive interventions are more economical than alternative options and can help prevent carers themselves from becoming ill.^95^ Despite global recognition of caregiver burden for both cancer and non-cancer diseases, research remains disproportionately focused on carers in Western or HICs.^96^ Given that caregiving experiences are shaped by cultural, behavioural and socioeconomic factors, the distinct characteristics, challenges and opportunities of LMICs need to be recognised.^97^ NPIs in the included studies were typically delivered in person, remotely or via a hybrid approach. There were no clear patterns of intervention effect based on delivery mode, suggesting flexibility in intervention design. This flexibility is important for tailoring interventions to local contexts, taking into account resource availability, training needs and digital readiness.

Both statistical heterogeneity and clinical heterogeneity were evident across studies. Statistical heterogeneity was present not only for outcomes with no clear effect, but also for some outcomes showing statistically significant pooled results. Clinical heterogeneity was substantial, with differences in intervention content, delivery mode (inperson, remote or hybrid), duration and outcome measures. Caregiver characteristics, dementia severity and variations in healthcare access may also have influenced observed effects. These sources of heterogeneity limit direct comparability between studies and reduce certainty in the pooled estimates. Given these factors, the current evidence should not be interpreted as definitive proof of effectiveness in all LMIC contexts, but rather as an indication of potential benefits that require further confirmation.

This review included multiple databases and journals; however, access to some sources became impossible due to website closures, server errors or search-term restrictions. Despite contacting authors, variations in measurement tools (including modified or adapted scales) presented challenges. Considerable heterogeneity in the included studies limited subgroup analysis, though the overall direction of effect was broadly consistent. Sensitivity analyses excluding studies at high risk of bias did not substantially change results. Other limitations include variability in the definition of ‘people with dementia’, ranging from clinical diagnoses to validated cognitive assessments, which may limit generalisability. The timing of outcome assessments was often unclear, restricting evaluation of sustained intervention effects. Few studies reported follow-up beyond immediate postintervention, meaning the durability of benefits remains uncertain. Recruitment methods were inconsistently reported, raising the possibility of selection bias, particularly in settings where dementia is underdiagnosed. In addition, the Template for Intervention Description and Replication checklist^98^ was not applied, which may limit replicability. Finally, the certainty of evidence was not assessed using the Grading of Recommendations Assessment, Development and Evaluation (GRADE) framework,^99 100^ reducing interpretability for policy and practice audiences.

While this review suggests that NPIs have potential to improve outcomes for informal dementia caregivers in LMICs, the evidence base is limited by heterogeneity, small sample sizes and incomplete reporting. The substantial differences in observed effect sizes remain largely unexplained, emphasising the need for well-designed, culturally adapted and contextually relevant interventions. Future research should prioritise consistent outcome measures, standardised follow-up periods, clear reporting of recruitment strategies and transparent intervention descriptions.

Rather than repeating small-scale effectiveness trials, a more efficient approach may be to co-design and adapt interventions from HICs for LMIC contexts, ensuring they are feasible within existing resource constraints. Such work should also consider digital readiness, workforce training needs and strategies to reach underdiagnosed and underserved populations.

## Conclusion

Overall, NPIs are effective in LMICs for informal dementia caregivers. Due to the heterogeneity of design and delivery, it is not possible to determine how the interventions work, why and due to what combination of components. Instead, there is a need to move from a climate of continuous effectiveness testing of RCTs to a pragmatic approach which considers how we culturally and contextually adapt and implement these effective interventions, so they get to those that need them most.

## Supplementary material

10.1136/bmjgh-2024-016028 online supplemental file 1

## Data Availability

Data are available upon reasonable request.
