# Supplementary material for: Effects of interventions on the psychosocial health and well-being of informal caregivers of people with dementia in low- and middle-income countries (LMICs): a systematic review and meta-analysis
Source: BMJ Glob Health. 2026 Jan 8;11(1):e016028. doi: 10.1136/bmjgh-2024-016028 (PMC13059920; doi:10.1136/bmjgh-2024-016028)
Supplement: online supplemental file 1 [file bmjgh-11-1-s001.docx]

**Supplementary Table 1: Full text articles excluded with rationale**

|  | **Reference of excluded study** | **Rationale for exclusion** |
| --- | --- | --- |
| 1 | de Mol van Otterloo NP. Minimizing Social Isolation in Low-income Communities: Practice, and Implementation Strategies for Dementia Care: University of Southern California; 2019. | Protocol with no published intervention results |
| 2 | Hinton L, Tran D, Nguyen T-N, Ho J, Gitlin L. Interventions to support family caregivers of people living with dementia in high, middle and low-income countries in Asia: a scoping review. 2019. | Review of existing literature, relevant for further scanning |
| 3 | ChiCTR1800015602. Chronic Disease Self-Management Support Programme for Spouse Caregivers of Persons with Dementia in China. <https://trialsearch.who.int/Trial2.aspx?TrialID=ChiCTR1800015602> [Internet]. 2019 Mar 31 [cited 2024 Mar 25]; Available from: <https://www.cochranelibrary.com/central/doi/10.1002/central/CN-01898785/full> | Trial registry record with no published results available |
| 4 | [CTRI/2021/01/030403. A study to develop and use digital media like videos for caregivers to improve dementia care in India. https://trialsearch.who.int/Trial2.aspx?TrialID=CTRI/2021/01/030403 [Internet]. 2021 Mar 31 [cited 2024 Mar 25]; Available from: https://www.cochranelibrary.com/central/doi/10.1002/central/CN-02239701/full?highlightAbstract=study%7Cvide%7Cuse%7Cfor%7Cvideos%7Cmedium%7Cmedia%7Cvideo%7Cindia%7Cmediums%7Ccaregivers%7Cdementi%7Cfour%7Cindi%7Cdigit%7Cdigital%7Ca%7Cin%7Clike%7Ccaregiv%7Cdevelop%7Cimprov%7Cmedi%7Cstudi%7Cimprove%7Cdementia%7Cto%7Ccare](https://www.cochranelibrary.com/central/doi/10.1002/central/CN-02239701/full?highlightAbstract=study%7Cvide%7Cuse%7Cfor%7Cvideos%7Cmedium%7Cmedia%7Cvideo%7Cindia%7Cmediums%7Ccaregivers%7Cdementi%7Cfour%7Cindi%7Cdigit%7Cdigital%7Ca%7Cin%7Clike%7Ccaregiv%7Cdevelop%7Cimprov%7Cmedi%7Cstudi%7Cimprove%7Cdementia%7Cto%7Ccare) | Trial registry record with no published results available |
| 5 | [PACTR201712002803135. Effect of group psycho-education and prayers on mental health of family caregivers of dementia patients attending a psychogeriatric clinic in Nigeria. https://trialsearch.who.int/Trial2.aspx?TrialID=PACTR201712002803135 [Internet]. 2019 Mar 31 [cited 2024 Mar 25]; Available from: https://www.cochranelibrary.com/central/doi/10.1002/central/CN-01884569/full?highlightAbstract=educ%7Ceducation%7Cattend%7Cmental%7Cfamili%7Ccaregivers%7Cpsychogeriatric%7Cdementi%7Cpatient%7Cattending%7Cpsychogeriatr%7Cof%7Cpsych%7Cgroup%7Ca%7Cnigeria%7Cin%7Cpatients%7Ccaregiv%7Chealth%7Cprayer%7Ceffect%7Cdementia%7Cclinic%7Cfamily%7Cprayers%7Cnigeri%7Cpsycho](https://www.cochranelibrary.com/central/doi/10.1002/central/CN-01884569/full?highlightAbstract=educ%7Ceducation%7Cattend%7Cmental%7Cfamili%7Ccaregivers%7Cpsychogeriatric%7Cdementi%7Cpatient%7Cattending%7Cpsychogeriatr%7Cof%7Cpsych%7Cgroup%7Ca%7Cnigeria%7Cin%7Cpatients%7Ccaregiv%7Chealth%7Cprayer%7Ceffect%7Cdementia%7Cclinic%7Cfamily%7Cprayers%7Cnigeri%7Cpsycho) | Trial registry record with no published results available |
| 6 | Luchsinger JA, Burgio L, Mittelman M, et alNorthern Manhattan Hispanic Caregiver Intervention Effectiveness Study: protocol of a pragmatic randomised trial comparing the effectiveness of two established interventions for informal caregivers of persons with dementia. *BMJ Open* 2016;6:e014082. doi: 10.1136/bmjopen-2016-014082 | Non-LMIC |
| 7 | Chen HM, Huang, MF, Yeh YC, Huang WH, Chen CS. (2015), Intervention for dementia caregivers. *Psychogeriatrics.* 2015;**15,**20-25. <https://doi.org/10.1111/psyg.12071> | Non-LMIC |
| 8 | Griffiths AW, Surr CA, Creese B, Garrod L, Chenoweth L. The development and use of the assessment of dementia awareness and person-centred care training tool in long-term care. Dementia. 2019;**18**(7-8):3059-70. doi:10.1177/1471301218768165 | Non-LMIC |
| 9 | Chen H, Chuengsatiansup K, Levkoff SE, He Y, Wandersman A, Lamont A, Chunharus S, Sihapark S, Thongsiri S, Pan T, Fritz S, Hinton L, Gallagher-Thompson D. P4-359: USING IMPLEMENTATION SCIENCE TO ADVANCE MANAGEMENT OF DEMENTIA-RELATED BEHAVIORAL PROBLEMS IN A DEVELOPING COUNTRY. *Alzheimer's & Dementia.* 2018 **14**: P1606-P1607. <https://doi.org/10.1016/j.jalz.2018.07.183> | Conference abstract (unable to obtain full study) |
| 10 | Hinton L, Nguyen H, Pham T, Trong HN, Harvey DJ, Thanh BN, Thi Thanh BN, Ngoc AN, Hong CN, Thi Hoai TN, Le TN, Nguyen T, Phuong A, Nguyen T Bich N, Tiet Q, Nguyen TA, Quy PN O3-14-04: A PILOT CLUSTER RCT TO TEST THE PRELIMINARY EFFICACY OF AN ALZHEIMER'S FAMILY CAREGIVER INTERVENTION IN HANOI, VIETNAM. *Alzheimer's & Dementia.* 2019 **15**: P924-P924. <https://doi.org/10.1016/j.jalz.2019.06.4705> | Conference abstract (unable to obtain full study) |
| 11 | Pires Camargo Novelli MM, Machado SC, Balestra de Lima G, Cantatore L, Pereira de Sena B, Rodrigues RS, Izys C, Fernandez Canon MB, Nitrini R, Gitlin LN, Piersol CV, Yassuda MS. P3-362: The Brazilian Version of Tailored Activity Program (Tap-BR) to Manage Neuropsychiatric Behaviors in Persons with Dementia and Reduce Caregiver Burden in Brazil: a Randomized Pilot Study. *Alzheimer's & Dementia.* 2016 **12**: P988-P988. <https://doi.org/10.1016/j.jalz.2016.06.2027> | Conference abstract (searched for & obtained full study) |
| 12 | Lee HJ, Kim KR, Seo JM. Effects of Telephone Counseling on Burnout, Depression, Life Satisfaction, and Perceived Health in Caregivers of Older Adults with Dementia. The journal of korean academic society of adult nursing 2003; **15**(3): 452‐462. | Unable to obtain English FT |
| 13 | Xiao. The evaluation of the Chinese iSupport for dementia program for caregivers in Australia and Greater China. 2021. Available from: <https://trialsearch.who.int/Trial2.aspx?TrialID=ACTRN12621000276853> | Protocol/registered trial with no results published yet |
| 14 | Nguyen Tran To Tran. (2023) A Mobile Phone-based Intervention on Dementia Patients' Caregivers in Vietnam. Available from: <https://clinicaltrials.gov/study/NCT04958707> | Protocol/registered trial with no results published yet |
| 15 | No authors provided (2022) A Phone Intervention for Family Dementia Caregivers in Vietnam. Available from: <https://clinicaltrials.gov/study/NCT05481320> | Protocol/registered trial with no results published yet |
| 16 | Nguyen TA, Tran K, Esterman A, Brijnath B, Xiao LD, Schofield P, Bhar S, Wickramasinghe N, Sinclair R, Dang TH, Cullum S, Turana Y, Hinton L, Seeher K, Andrade AQ, Crotty M, Kurrle S, Freel S, Pham T, Nguyen TB, Brodaty H  Empowering Dementia Carers With an iSupport Virtual Assistant (e-DiVA) in Asia-Pacific Regional Countries: Protocol for a Pilot Multisite Randomized Controlled Trial  *JMIR Res Protoc* 2021;**10**(11):e33572. doi: 10.2196/33572 | Protocol/registered trial with no results published yet |
| 17 | Araujo and Lacerda. The Effect of Alzheimer's Disease Education Associated with Meditation on Family Caregivers' Stress and Quality of Life. 2022. Available from: <https://trialsearch.who.int/Trial2.aspx?TrialID=RBR-794593r> | Protocol/registered trial with no results published yet |
| 18 | Arendts G, Chenoweth L, Hayes B. et al. CELPI: trial protocol for a randomised controlled trial of a Carer End of Life Planning Intervention in people dying with dementia. *BMC Geriatr* 2022;**22**,869. <https://doi.org/10.1186/s12877-022-03534-1> | Protocol/registered trial with no results published yet |
| 19 | Sarvolia. Investigating the impact of virtual reality on health and anxiety and care of Alzheimer's patients. 2022. Available from: <https://trialsearch.who.int/Trial2.aspx?TrialID=IRCT20221123056586N1> | Protocol/registered trial with no results published yet |
| 20 | Nakigudde and Nakasujja. Evaluation of the Uganda Adopted version of the WHO-iSupport intervention for caregivers of persons with Alzheimer's disease and related Dementias (ADRD). 2022. Available from: <https://trialsearch.who.int/Trial2.aspx?TrialID=PACTR202211700581839> | Protocol/registered trial with no results published yet |
| 21 | Chen H, Levkoff S, Chuengsatiansup K, et al Implementation Science in Thailand: Design and Methods of a Geriatric Mental Health Cluster-Randomized Trial. *Psychiatric Services*. 2022;**73**(1):83-91. | Non-caregiver focused |
| 22 | Lu W, Mao Q. The effects of family follow-up nursing on elderly cognitive impairment patients' Barthel index scores and mental statuses. *Am J Transl Res*. 2021;**13**(6):6702-6709. PMID: 34306415; PMCID: PMC8290769 | Non-caregiver focused |
| 23 | Fink R. The Reality of Family Caregiving for Latinos with Alzheimer’s Disease and Related Dementias: A Qualitative Analysis. *JPSM*. 2022. Accessed <https://www.jpsmjournal.com/article/S0885-3924(22)00127-0/pdf> | Experiences/perceptions, not intervention |
| 24 | Corcoran MA, Gitlin LN. Family caregiver acceptance and use of environmental strategies provided in an occupational therapy intervention. Physical & Occupational Therapy in Geriatrics. 2001;19(1):1-20. | Non-LMIC |
| 25 | Ha J-H, Kwak M, Han JW, Lee HJ, Ingersoll-Dayton B, Spencer B, et al. The effectiveness of a couple-based intervention for people living with mild Alzheimer's disease and their spousal caregivers in Korea. Dementia (14713012). 2021;20(3):831-47. | Non-LMIC |
| 26 | Chou H-K, Yan S-H, Lin I-C, Tsai M-TC, Chu-Chieh Woung, Lin-Chung. A Pilot Study of the Telecare Medical Support System as an Intervention in Dementia Care: The Views and Experiences of Primary Caregivers. Journal of Nursing Research (Lippincott Williams & Wilkins). 2012;20(3):169-80. | Non-LMIC |
| 27 | Kurz A, Bakker C, Böhm M, Diehl-Schmid J, Dubois B, Ferreira C, et al. RHAPSODY - Internet-based support for caregivers of people with young onset dementia: program design and methods of a pilot study. International Psychogeriatrics. 2016;28(12):2091-9. | Non-LMIC |
| 28 | Parveen S, Blakey H, Oyebode JR. Evaluation of a carers' information programme culturally adapted for South Asian families. International Journal of Geriatric Psychiatry. 2018;33(2):e199-e204. | Non-LMIC |
| 29 | Kehoua G, Dubreuil CM, Ndamba‐Bandzouzi B, Guerchet M, Mbelesso P, Dartigues JF, et al. From the social representation of the people with dementia by the family carers in Republic of Congo towards their conviction by a customary jurisdiction, preliminary report from the EPIDEMCA-FU study. Hoboken, New Jersey: John Wiley & Sons, Inc.; 2016. p. 1254-5. | Article/bulletin/letter to editor, not intervention |
| 30 | Wang H, Li T, Barbarino P, Gauthier S, Brodaty H, Molinuevo JL, et al. Dementia care during COVID-19. Philadelphia, Pennsylvania: Lancet; 2020. p. 1190-1. | Article/bulletin/letter to editor, not intervention |
| 31 | Balakrishnan VSaF, F. Supporting families to care for people with dementia. Bulletin of the World Health Organization. 2017;95(11):734-5. | Article/bulletin/letter to editor, not intervention |
| 32 | Hui Z, Yang C, Li J, Lee DTF. Effectiveness of an online education intervention on stress and coping of family members after placing a relative with dementia into a residential care facility: protocol of a randomised controlled trial. BMC Geriatrics. 2020;20(1):N.PAG-N.PAG. | Protocol/registered trial with no results published yet |
| 33 | Mehta KM, Gallagher-Thompson D, Varghese M, Loganathan S, Baruah U, Seeher K, et al. iSupport, an online training and support program for caregivers of people with dementia: study protocol for a randomized controlled trial in India. Trials. 2018;19(1):N.PAG-N.PAG. | Protocol/registered trial. Followed up but feasibility RCT |
| 34 | Nguyen TA, Nguyen H, Pham T, Nguyen TH, Hinton L. A cluster randomized controlled trial to test the feasibility and preliminary effectiveness of a family dementia caregiver intervention in Vietnam: The REACH VN study protocol. Medicine. 2018;97(40):e12553-e. | Protocol/registered trial. Followed up but feasibility RCT |
| 35 | Kerr D. Developing dementia care services across India. Journal of Dementia Care. 2004;12(4):20-1. | Overview of services, not intervention |
| 36 | Wang S, Cheung DSK, Leung AYM. Overview of dementia care under the three‐tier long‐term care system of China. Public Health Nursing. 2019;36(2):199-206. | Overview of services, not intervention |
| 37 | Wu B, Cohen MA, Cong Z, Kim K, Peng C. Improving Care for Older Adults in China: Development of Long-Term Care Policy and System. Research on Aging. 2021;43(3/4):123-6. | Overview of services, not intervention |
| 38 | Resciniti NV, Tang W, Tabassum M, Pearson JL, Spencer SM, Lohman MC, et al. Knowledge evaluation instruments for dementia caregiver education programs: A scoping review. Geriatrics & Gerontology International. 2020;20(5):397-413. | Review of existing literature, relevant for further scan |
| 39 | Shi H, Mao C, Tang J, Liang H. Research on the health of and interventions for family caregivers of people with dementia: a bibliometric analysis of research output during 1988-2018. BMC Geriatrics. 2020;20(1):1-9. | Review of existing literature, relevant for further scan |
| 40 | Tak Y, Song J, Woo H, An J. Realist Review: Understanding Effectiveness of Intervention Programs for Dementia Caregivers. Asian Nursing Research. 2019;13(1):11-9. | Review of existing literature, relevant for further scan |
| 41 | Lasrado R, Baker S, Zubair M, Kaiser P, Lasrado VJ, Rizzo M, et al. Exploring Dementia Care Systems Across the African Caribbean Diaspora: A Scoping Review and Consultation Exercise. Gerontologist. 2021;61(5):e209-e27. | Review of existing literature, relevant for further scan |
| 42 | Aravena JM, Gajardo J, Saguez R, Hinton L, Gitlin LN. Nonpharmacologic Interventions for Family Caregivers of People Living With Dementia in Latin-America: A Scoping Review. American Journal of Geriatric Psychiatry. 2022;30(8):859-77. | Review of existing literature, relevant for further scan |
| 43 | Sun Y, Ji M, Leng M, Wang Z. Which cognitive behavioral therapy delivery formats work for depressive symptoms in dementia caregivers? - A systematic review and network meta-analysis of randomized controlled trials. Journal of Affective Disorders. 2022;308:181-7. | Review of existing literature, relevant for further scan |
| 44 | Senanarong V, Jamjumras P, Harmphadungkit K, Klubwongs M, Udomphanthurak S, Poungvarin N, et al. A counseling intervention for caregivers: effect on neuropsychiatric symptoms. International Journal of Geriatric Psychiatry. 2004;19(8):781-8. | Non-randomised |
| 45 | Zhang SY, Wu F, Tang DL, Rong XS, Guo QH, Fang M, et al. Pilot testing the caregiver self-management intervention for caregivers of relatives with dementia. Geriatric Nursing. 2020;41(2):147-57. | Non-randomised |
| 46 | Sanprakhon P, Chaimongkol N, Hengudomsub P, Lach HW. An Integrative Stress Reduction Program for Family Caregivers of Persons With Advanced Dementia: A Pilot Study. J Gerontol Nurs. 2022;48(10):26-32. | Non-RCT |
| 47 | Han JW, Jeong H, Park JY, Kim TH, Lee DY, Lee DW, et al. Effects of social supports on burden in caregivers of people with dementia. International Psychogeriatrics. 2014;26(10):1639-48. | Exploring factors of needs/unmet needs rather than intervention |
| 48 | Ineu de Oliveira T, Rodrigues Maziero B, Ilha S, Segabinazzi Pacheco L, Schroeder de Oliveira F. DAILY FAMILY MEMBERS/CAREGIVER WITH ALZHEIMER: SUPPORT GROUP CONTRIBUTIONS. Journal of Nursing UFPE / Revista de Enfermagem UFPE. 2017;11(2):506-14. | Exploring factors of needs/unmet needs rather than intervention |
| 49 | Noimuenwai P. Effectiveness of adult day care programs on health outcomes of Thai family caregivers of persons with dementia: University of Kansas; 2012. | Intervention not delivered to the caregiver |
| 50 | Turten Kaymaz T, Ozdemir L. Effects of aromatherapy on agitation and related caregiver burden in patients with moderate to severe dementia: A pilot study. Geriatric Nursing. 2017;38(3):231-7. | Intervention not delivered to the caregiver |
| 51 | Ilha S, Costa Santos SS, Stein Backes D, Lima Barros EJ, Teda Pelzer M, Santini Costenaro RG. Complex educational and care (geron)technology for elderly individuals/families experiencing Alzheimer's disease. Revista Brasileira de Enfermagem. 2017;70(4):726-32. | Non-controlled trial |
| 52 | Lamech N, Lakshminarayanan M, Vaitheswaran S, John S, Rangaswamy T. Support groups for family caregivers of persons with dementia in India. Dementia (14713012). 2021;20(3):1172-81. | Non-controlled trial |
| 53 | McCabe L. Translating policy and practice: day care for people with dementia in Kerala, India. Global Social Policy. 2007;7(2):203-21. | Non-informal caregiver focused |
| 54 | Karungi CK, Wakida EK, Rukundo GZ, Talib ZM, Haberer JE, Obua C. Lay Health Workers in Community-Based Care and Management of Dementia: A Qualitative 'Pre' and 'Post' Intervention Study in Southwestern Uganda. BioMed Research International. 2022:1-10. | Non-informal caregiver focused |
| 55 | Ojagbemi A, Daley S. Implementing the Dementia Carers Support Initiative of the National Institute for Health and Care Excellence in a sub-Saharan African Setting. Journal of Health Care for the Poor & Underserved. 2015;26(4):1368-76. | Critique of policy, not intervention |
| 56 | Ta Park VM, Ton V, Tiet QQ, Vuong Q, Yeo G, Gallagher-Thompson D. Promising results from a pilot study to reduce distress in Vietnamese American dementia and memory loss caregivers. Alzheimer's and Dementia: Translational Research and Clinical Interventions. 2019;5:319-27. | Non-LMIC |
| 57 | Choi Y, Park S, Cho KH, Chun SY, Park EC. A change in social activity affect cognitive function in middle-aged and older Koreans: Analysis of a Korean longitudinal study on aging (2006-2012). International Journal of Geriatric Psychiatry. 2016. | Non-LMIC |
| 58 | Harbi E. Effectiveness of mindfulness-based cognitive therapy and conscious group yoga on depression, anxiety, and stress of female caregivers of elderlies with Alzheimer. Annals of Tropical Medicine and Public Health. 2018;2(Special Issue):104-15. | Non-randomised |
| 59 | Shetty P. Grey matter: Ageing in developing countries. The Lancet. 2012;379(9823):1285-7. | Non-dementia focused |
| 60 | Govindakumari R, Vijayalakshmi, Goothy SSK, Raghvan V. Effectiveness of home based cognitive training programme on quality of life and caregiver burden among elderly persons with mild cognitive impairment in selected community settings of puducherry. Indian Journal of Public Health Research and Development. 2020;11(5):360-3. | Non-informal caregiver focused - reported caregiver burden but not part of participants |
| 61 | Rodrigues L, Mathias T. Effectiveness of planned teaching program on knowledge regarding Alzheimer's disease among the family members of elderly in a selected urban community at Mangalore. Indian Journal of Psychiatry. 2016;58(1):44-8. | Caregivers of elderly who did not necessarily have dementia, despite intervention being dementia-focused |
| 62 | Baruah U, Loganathan S, Shivakumar P, Pot AM, Mehta KM, Gallagher-Thompson D, et al. Adaptation of an online training and support program for caregivers of people with dementia to Indian cultural setting. Asian J Psychiatr. 2021;59 (no pagination). | Development process/adaption/validation of a tool/program, no outcomes of intervention |
| 63 | Anonymous. Addressing global dementia. The Lancet. 2014;383(9936):2185. | Editorial/article, no intervention |
| 64 | Nankinga PN, Maling S, Chemali Z, Wakida EK, Obua C, Okello ES. Informal support for people with Alzheimer's disease and related dementias in rural Uganda: A qualitative study. International Journal of Mental Health Systems. 2020;14(1). | Experiences/perceptions, not intervention |
| 65 | Ogunniyi A, Hall KS, Baiyewu O, Gureje O, Unverzagt FW, Gao S, et al. Caring for individuals with dementia. The Nigerian experience. West African Journal of Medicine. 2005;24(3):259-62. | Review/comparison of existing literature, relevant for further scan |
| 66 | Rong X, Wu F, Zhang S. ECN Award: Effect of a Chronic Disease Self-Management Support Program for Spouse Caregivers of Relatives with Dementia in Shanghai, China: A Randomised Controlled Study. International Psychogeriatrics. 2021;33(SUPPL 1):18. | Conference abtract (searched for & full study not published) |
| 67 | Huong TTT, Tam PTT, Luu PT, An NTT, Liem DT, Truc NTT, et al. DEMENTIA CAREGIVER SUPPORT PROGRAM IN VIETNAM: DEVELOPMENT, IMPLEMENTATION AND FIRST RESULTS. Alzheimer's and Dementia. 2022;18(S8):e062110. | Conference abtract (searched for & full study not published) |
| 68 | Karanja W, Mutunga EK, Timmons F. Public health policies, dementia care and support in LMICs. Alzheimer's and Dementia. 2022;18(S8):e062908. | Conference abtract (searched for & full study not published) |
| 69 | Vaitheswaran S, Natarajan N, Ramanujam V, S S, Venkatesan S, Srinivasan N, et al. Using Technology to Overcome the Challenges of COVID-19 Pandemic in India: The DEMCARES Model of Virtual Intervention Delivery. Alzheimer's & dementia : the journal of the Alzheimer's Association. 2022;18(Supplement 9):e065137. | Conference abtract (searched for & full study not published) |
| 70 | Webster MJ, Bouranis NG, Gitlin LN, Gliebus GP. Developing the 'Good Life' Model: The Recruitment and Training of 'Dementia Ambassadors' to Provide a Novel Coaching Approach for Family Caregivers. Alzheimer's & dementia : the journal of the Alzheimer's Association. 2022;18(Supplement 9):e062616. | Conference abtract (searched for & full study not published) |
| 71 | Lee JA, Ju E, Zhang J, Sabino-Laughlin E, Rahmani A, Gibbs L, et al. Culturally and linguistically appropriate home visit intervention for underserved dementia family caregivers: Preliminary results from a randomized controlled trial. Alzheimer's and Dementia. 2022;18(S8):e058927. | Non-LMIC |
| 72 | Morris L, Innes A, Smith S, Wilson J, Bushell S, Wyatt M. A qualitative evaluation of the impact of a Good Life Club on people living with dementia and care partners. Dementia (London, England). 2021;20(7):2478-93. | Non-LMIC |
| 73 | Jeste DV, Mausbach B, Lee EE. Caring for caregivers/care partners of persons with dementia. International Psychogeriatrics. 2021;33(4):307-10. | Exploring factors of needs/unmet needs rather than intervention |
| 74 | Grinberg A, Lagunoff J, Phillips D, Stern B, Goodman M, Chow T. Multidisciplinary design and implementation of a day program specialized for the frontotemporal dementias. American Journal of Alzheimer's Disease & Other Dementias. 2007;22(6):499-506. | Non-LMIC |
| 75 | Kim JS. Daughters-in-law in Korean caregiving families. J Adv Nurs. 2001;36(3):399-408. | Non-LMIC |
| 76 | Juengst S, Supnet C, Kew CLN, Silva V, Vega M, Han G, et al. Bilingual problem-solving training for caregivers of adults with dementia: A randomized, factorial-design protocol for the CaDeS trial. Contemporary clinical trials. 2021;108:106506. | Non-LMIC |
| 77 | Gitlin LN, Marx K, Piersol CV, Hodgson NA, Parker LJ, Cidav T, et al. Differential race effects of the tailored activity program (TAP) on dementia-related behaviors: A randomized controlled trial. Journal of the American Geriatrics Society. 2022;70(11):3105-15. | Non-LMIC |
| 78 | Raghuraman S, Lakshminarayanan M, Vaitheswaran S, Rangaswamy T. Cognitive Stimulation Therapy for Dementia: Pilot Studies of Acceptability and Feasibility of Cultural Adaptation for India. American Journal of Geriatric Psychiatry. 2017;25(9):1029-32. | Non-informal caregiver focused |
| 79 | Stoner CR, Lakshminarayanan M, Mograbi DC, Vaitheswaran S, Bertrand E, Schimidt Brum P, et al. Development and acceptability of a brief, evidence-based Dementia Awareness for Caregivers course in low- and middle-income countries. Dementia (London, England). 2022;21(2):598-617. | Non-caregiver focused (did not collect baseline data) |
| 80 | Magteppong W, Yamarat K. The Effects of the Modified Transtheoretical Theory of Stress and Coping (TTSC) Program on Dementia Caregivers' Knowledge, Burden, and Quality of Life. International journal of environmental research and public health. 2021;18(24). | Non-RCT |
| 81 | Xiao LD, Wang J, Ratcliffe J, Ullah S, Brodaty H, Brijnath B, et al. A nurse-led multicentre randomized controlled trial on effectiveness and cost-effectiveness of Chinese iSupport for dementia program: A study protocol. J Adv Nurs. 2022;78(5):1524-33. | Non-RCT |
| 82 | Javadpour A, Ahmadzadeh L, Bahredar MJ. An educative support group for female family caregivers: impact on caregivers psychological distress and patient's neuropsychiatry symptoms. International Journal of Geriatric Psychiatry. 2009;24(5):469-71. | Non-controlled trial |
| 83 | Marinho V, Bertrand E, Naylor R, Bomilcar I, Laks J, Spector A, et al. Cognitive stimulation therapy for people with dementia in Brazil (CST-Brasil): Results from a single blind randomized controlled trial. International Journal of Geriatric Psychiatry. 2021;36(2):286-93. | Intervention not delivered to the caregiver |
| 84 | Xiao LD, Wang J, Ratcliffe J, Ullah S, Brodaty H, Brijnath B, et al. A nurse-led multicentre randomized controlled trial on effectiveness and cost-effectiveness of Chinese iSupport for dementia program: A study protocol. J Adv Nurs. 2022;78(5):1524-33. | Protocol, no intervention results |
| 85 | Tongsiri S, Levkoff S, Gallagher-Thompson D, Teri L, Hinton L, Wisetpholchai B, et al. Cultural Adaptation of the Reducing Disability in Alzheimer's Disease (RDAD) Protocol for an Intervention to Reduce Behavioral and Psychological Symptoms of Dementia in Thailand. Journal of Alzheimer's disease : JAD. 2022;87(4):1603-14. | Conference abtract (searched for & full study not published |
| 86 | Pot AM, Gallagher-Thompson D, Xiao LD, Willemse BM, Rosier I, Mehta KM, et al. iSupport: a WHO global online intervention for informal caregivers of people with dementia. World Psychiatry. 2019;18(3):365-6. | Article, not intervention |
| 87 | Ali S, Bokharey IZ. Efficacy of cognitive behavior therapy among caregivers of dementia: An outcome study. Pakistan Journal of Psychological Research. 2015;30(2):249-69. | Non-controlled trial |
| 88 | Pot AM, Egan K, Seeher K. Internet-based interventions for family carers of people with dementia. Improving the lives of people with dementia through technology: Interdisciplinary network for dementia utilising current technology. 2023:150-60. | Book chapter, not intervention |
| 89 | Berkemer E, Bär M. [Outreach dyadic support program for persons with dementia and relatives by dementia nursing experts: qualitative results from a pilot study in rural areas]. Z Gerontol Geriatr. 2022. | Non-LMIC |
| 90 | Boessen A, Verwey R, Duymelinck S, van Rossum E. An Online Platform to Support the Network of Caregivers of People with Dementia. J Aging Res. 2017;2017:3076859. | Non-LMIC |
| 91 | Braly T, Muriathiri D, Brown JC, Taylor BM, Boustani MA, Holden RJ. Technology intervention to support caregiving for Alzheimer's disease (I-CARE): study protocol for a randomized controlled pilot trial. Pilot Feasibility Stud. 2021;7(1):23. | Non-LMIC |
| 92 | Burns R, Nichols LO, Martindale-Adams J, Graney MJ, Lummus A. Primary care interventions for dementia caregivers: 2-year outcomes from the REACH study. Gerontologist. 2003;43(4):547-55. | Non-LMIC |
| 93 | Cheung DSK, Ho LYW, Chan LCK, Kwok RKH, Lai CKY. A Home-Based Dyadic Music-with-Movement Intervention for People with Dementia and Caregivers: A Hybrid Type 2 Cluster-Randomized Effectiveness-Implementation Design. Clin Interv Aging. 2022;17:1199-216. | Non-LMIC |
| 94 | Contreras M, Van Hout E, Farquhar M, McCracken LM, Gould RL, Hornberger M, et al. Internet-delivered guided self-help Acceptance and Commitment Therapy for family carers of people with dementia (iACT4CARERS): a qualitative study of carer views and acceptability. Int J Qual Stud Health Well-being. 2022;17(1):2066255. | Non-LMIC |
| 95 | Cravello L, Martini E, Viti N, Campanello C, Assogna F, Perotta D. Effectiveness of a Family Support Intervention on Caregiving Burden in Family of Elderly Patients With Cognitive Decline After the COVID-19 Lockdown. Front Psychiatry. 2021;12:590104. | Non-LMIC |
| 96 | Cuc AV, Locke DEC, Duncan N, Fields JA, Snyder CH, Hanna S, et al. A pilot randomized trial of two cognitive rehabilitation interventions for mild cognitive impairment: caregiver outcomes. Int J Geriatr Psychiatry. 2017;32(12):e180-e7. | Non-LMIC |
| 97 | Dam AEH, de Vugt ME, van Boxtel MPJ, Verhey FRJ. Effectiveness of an online social support intervention for caregivers of people with dementia: the study protocol of a randomised controlled trial. Trials. 2017;18(1):395. | Non-LMIC |
| 98 | Damianakis T, Wilson K, Marziali E. Family caregiver support groups: spiritual reflections' impact on stress management. Aging Ment Health. 2018;22(1):70-6. | Non-LMIC |
| 99 | Dröes RM, van Rijn A, Rus E, Dacier S, Meiland F. Utilization, effect, and benefit of the individualized Meeting Centers Support Program for people with dementia and caregivers. Clin Interv Aging. 2019;14:1527-53. | Non-LMIC |
| 100 | Duggleby W, Jovel Ruiz K, Ploeg J, McAiney C, Peacock S, Nekolaichuk C, et al. Mixed-methods single-arm repeated measures study evaluating the feasibility of a web-based intervention to support family carers of persons with dementia in long-term care facilities. Pilot Feasibility Stud. 2018;4:165. | Non-LMIC |
| 101 | Fauth EB, Novak JR, Levin ME. Outcomes from a pilot online Acceptance and Commitment Therapy program for dementia family caregivers. Aging Ment Health. 2022;26(8):1620-9. | Non-LMIC |
| 102 | Finkel S, Czaja SJ, Schulz R, Martinovich Z, Harris C, Pezzuto D. E-care: a telecommunications technology intervention for family caregivers of dementia patients. Am J Geriatr Psychiatry. 2007;15(5):443-8. | Non-LMIC |
| 103 | Gaugler JE, Reese M, Tanler R. Care to Plan: An Online Tool That Offers Tailored Support to Dementia Caregivers. Gerontologist. 2016;56(6):1161-74. | Non-LMIC |
| 104 | Gaugler JE, Statz TL, Birkeland RW, Louwagie KW, Peterson CM, Zmora R, et al. The ResidentialCare Transition Module: a single-blinded randomized controlled evaluation of a telehealth support intervention for family caregivers of persons with dementia living in residential long-term care. BMC Geriatr. 2020;20(1):133. | Non-LMIC |
| 105 | Gerdner LA, Buckwalter KC, Reed D. Impact of a psychoeducational intervention on caregiver response to behavioral problems. Nurs Res. 2002;51(6):363-74. | Non-LMIC |
| 106 | Glueckauf RL, Ketterson TU, Loomis JS, Dages P. Online support and education for dementia caregivers: overview, utilization, and initial program evaluation. Telemed J E Health. 2004;10(2):223-32. | Non-LMIC |
| 107 | Glueckauf RL, Loomis JS. Alzheimer's Caregiver Support Online: lessons learned, initial findings and future directions. NeuroRehabilitation. 2003;18(2):135-46. | Non-LMIC |
| 108 | Han A, Kim TH, Hong H. A factorial randomized controlled trial to examine separate and combined effects of a simulation-based empathy enhancement program and a lecture-based education program on family caregivers of people with dementia. Aging Ment Health. 2021;25(10):1930-40. | Non-LMIC |
| 109 | Hepburn K, Lewis M, Tornatore J, Sherman CW, Bremer KL. The Savvy Caregiver program: the demonstrated effectiveness of a transportable dementia caregiver psychoeducation program. J Gerontol Nurs. 2007;33(3):30-6. | Non-LMIC |
| 110 | Hepburn K, Nocera J, Higgins M, Epps F, Brewster GS, Lindauer A, et al. Results of a Randomized Trial Testing the Efficacy of Tele-Savvy, an Online Synchronous/Asynchronous Psychoeducation Program for Family Caregivers of Persons Living with Dementia. Gerontologist. 2022;62(4):616-28. | Non-LMIC |
| 111 | Jenewein J, Moergeli H, Meyer-Heim T, Muijres P, Bopp-Kistler I, Chochinov HM, et al. Feasibility, Acceptability, and Preliminary Efficacy of Dignity Therapy in Patients With Early Stage Dementia and Their Family. A Pilot Randomized Controlled Trial. Front Psychiatry. 2021;12:795813. | Non-LMIC |
| 112 | Judge KS, Yarry SJ, Orsulic-Jeras S. Acceptability and feasibility results of a strength-based skills training program for dementia caregiving dyads. Gerontologist. 2010;50(3):408-17. | Non-LMIC |
| 113 | Kajiyama B, Thompson LW, Eto-Iwase T, Yamashita M, Di Mario J, Marian Tzuang Y, et al. Exploring the effectiveness of an internet-based program for reducing caregiver distress using the iCare Stress Management e-Training Program. Aging Ment Health. 2013;17(5):544-54. | Non-LMIC |
| 114 | Kor PPK, Li ML, Kwok DKS, Leung AYM, Lai DLL, Liu JYW. Evaluating the effectiveness of a 6-week hybrid mindfulness-based intervention in reducing the stress among caregivers of patients with dementia during COVID-19 pandemic: protocol of a randomized controlled trial. BMC Psychol. 2022;10(1):178. | Non-LMIC |
| 115 | Kor PPK, Yu CTK, Liu JYW, Cheung DSK, Kwan RYC, Leung AYM, et al. Pilot evaluation of a home-based multi-sensory cognitive stimulation intervention for older people with dementia and caregiver dyads during the COVID-19 pandemic. Int J Older People Nurs. 2022;17(6):e12471. | Non-LMIC |
| 116 | Kovaleva M, Nocera JR, Hepburn K, Higgins M, Nash R, Epps F, et al. Attention control in a trial of an online psychoeducational intervention for caregivers. Res Nurs Health. 2022;45(3):337-49. | Non-LMIC |
| 117 | Kwok T, Lam L, Chung J. Case management to improve quality of life of older people with early dementia and to reduce caregiver burden. Hong Kong Med J. 2012;18 Suppl 6:4-6. | Non-LMIC |
| 118 | Lu YY, Ellis J, Yang Z, Weaver MT, Bakas T, Austrom MG, et al. Satisfaction With a Family-Focused Intervention for Mild Cognitive Impairment Dyads. J Nurs Scholarsh. 2016;48(4):334-44. | Non-LMIC |
| 119 | Marziali E, Donahue P. Caring for others: Internet video-conferencing group intervention for family caregivers of older adults with neurodegenerative disease. Gerontologist. 2006;46(3):398-403. | Non-LMIC |
| 120 | Mavandadi S, Wray LO, Toseland RW. Measuring Self-Appraised Changes following Participation in an Intervention for Caregivers of Individuals with Dementia. J Gerontol Soc Work. 2019;62(3):324-37. | Non-LMIC |
| 121 | Mizuno E, Hosak T, Ogihara R, Higano H, Mano Y. Effectiveness of a stress management program for family caregivers of the elderly at home. J Med Dent Sci. 1999;46(4):145-53. | Non-LMIC |
| 122 | Novais T, Moutet C, Delphin-Combe F, Dauphinot V, Colin C, Krolak-Salmon P, et al. PHARMAID study protocol: Randomized controlled trial to assess the impact of integrated pharmaceutical care at a psychosocial intervention on caregiver's burden in Alzheimer's disease or related diseases. Contemp Clin Trials. 2017;53:137-42. | Non-LMIC |
| 123 | Pasquini S, Margaritini A, Gambella E, Di Rosa M, Maranesi E, Bevilacqua R, et al. A Psychosocial Intervention for Supporting Informal Caregivers of Older People With Alzheimer Disease: Protocol for the InnFamiglia Randomized Controlled Trial. JMIR Res Protoc. 2022;11(11):e37496. | Non-LMIC |
| 124 | Perales-Puchalt J, Ramírez-Mantilla M, Fracachán-Cabrera M, Vidoni ED, Ellerbeck EF, Ramírez AS, et al. A text message intervention to support latino dementia family caregivers (CuidaTEXT): feasibility study. Clin Gerontol. 2022:1-16. | Non-LMIC |
| 125 | Pot AM, Blom MM, Willemse BM. Acceptability of a guided self-help Internet intervention for family caregivers: mastery over dementia. Int Psychogeriatr. 2015;27(8):1343-54. | Non-LMIC |
| 126 | Prick AE, de Lange J, Twisk J, Pot AM. The effects of a multi-component dyadic intervention on the psychological distress of family caregivers providing care to people with dementia: a randomized controlled trial. Int Psychogeriatr. 2015;27(12):2031-44. | Non-LMIC |
| 127 | Roth DL, Mittelman MS, Clay OJ, Madan A, Haley WE. Changes in social support as mediators of the impact of a psychosocial intervention for spouse caregivers of persons with Alzheimer's disease. Psychol Aging. 2005;20(4):634-44. | Non-LMIC |
| 128 | Schmitter-Edgecombe M, Dyck DG. Cognitive rehabilitation multi-family group intervention for individuals with mild cognitive impairment and their care-partners. J Int Neuropsychol Soc. 2014;20(9):897-908. | Non-LMIC |
| 129 | Serafini JD, Damianakis T, Marziali E. Clinical practice standards and ethical issues applied to a virtual group intervention for spousal caregivers of people with Alzheimer's. Soc Work Health Care. 2007;44(3):225-43. | Non-LMIC |
| 130 | Song Y, McCurry SM, Lee D, Josephson KR, McGowan SK, Fung CH, et al. Development of a dyadic sleep intervention for Alzheimer's disease patients and their caregivers. Disabil Rehabil. 2021;43(13):1861-71. | Non-LMIC |
| 131 | Testad I, Clare L, Anstey K, Selbæk G, Bjørkløf GH, Henderson C, et al. Self-management and HeAlth Promotion in Early-stage dementia with e-learning for carers (SHAPE): study protocol for a multi-centre randomised controlled trial. BMC Public Health. 2020;20(1):1508. | Non-LMIC |
| 132 | Töpfer NF, Sittler MC, Lechner-Meichsner F, Theurer C, Wilz G. Long-term effects of telephone-based cognitive-behavioral intervention for family caregivers of people with dementia: Findings at 3-year follow-up. J Consult Clin Psychol. 2021;89(4):341-9. | Non-LMIC |
| 133 | Tremont G, Davis JD, Ott BR, Galioto R, Crook C, Papandonatos GD, et al. Randomized Trial of the Family Intervention: Telephone Tracking-Caregiver for Dementia Caregivers: Use of Community and Healthcare Resources. J Am Geriatr Soc. 2017;65(5):924-30. | Non-LMIC |
| 134 | Van Mierlo LD, Meiland FJ, Van de Ven PM, Van Hout HP, Dröes RM. Evaluation of DEM-DISC, customized e-advice on health and social support services for informal carers and case managers of people with dementia; a cluster randomized trial. Int Psychogeriatr. 2015;27(8):1365-78. | Non-LMIC |
| 135 | Wijma EM, Veerbeek MA, Prins M, Pot AM, Willemse BM. A virtual reality intervention to improve the understanding and empathy for people with dementia in informal caregivers: results of a pilot study. Aging Ment Health. 2018;22(9):1115-23. | Non-LMIC |
| 136 | Yeung CC, Ho KH, Chan HY. A dyadic advance care planning intervention for people with early-stage dementia and their family caregivers in a community care setting: a feasibility trial. BMC Geriatr. 2023;23(1):115. | Non-LMIC |
| 137 | Zwingmann I, Hoffmann W, Michalowsky B, Dreier-Wolfgramm A, Hertel J, Wucherer D, et al. Supporting family dementia caregivers: testing the efficacy of dementia care management on multifaceted caregivers' burden. Aging Ment Health. 2018;22(7):889-96. | Non-LMIC |
| 138 | Leng M, Zhao Y, Xiao H, Li C, Wang Z. Internet-Based Supportive Interventions for Family Caregivers of People With Dementia: Systematic Review and Meta-Analysis. J Med Internet Res. 2020;22(9):e19468. | Review of existing literature, relevant for further screening |
| 139 | Ma KPK, Saw A. An international systematic review of dementia caregiving interventions for Chinese families. Int J Geriatr Psychiatry. 2020;35(11):1263-84. | Review of existing literature, relevant for further screening |
| 140 | Qiu D, Hu M, Yu Y, Tang B, Xiao S. Acceptability of psychosocial interventions for dementia caregivers: a systematic review. BMC Psychiatry. 2019;19(1):23. | Review of existing literature, relevant for further screening |
| 141 | Zhao Y, Feng H, Hu M, Hu H, Li H, Ning H, et al. Web-Based Interventions to Improve Mental Health in Home Caregivers of People With Dementia: Meta-Analysis. J Med Internet Res. 2019;21(5):e13415. | Review of existing literature, relevant for further screening |
| 142 | Ayoub MF, de Souza YLP, de Almeida T, Falcão D. Synchronous psychological interventions by videoconferencing for caregivers of people with dementia: an integrative review. Dement Neuropsychol. 2022;16(1):1-7. | Review of existing literature, relevant for further screening |
| 143 | Byeon H. Effects of Grief Focused Intervention on the Mental Health of Dementia Caregivers: Systematic Review and Meta-Analysis. Iran J Public Health. 2020;49(12):2275-86. | Review of existing literature, relevant for further screening |
| 144 | Cheng ST, Au A, Losada A, Thompson LW, Gallagher-Thompson D. Psychological Interventions for Dementia Caregivers: What We Have Achieved, What We Have Learned. Curr Psychiatry Rep. 2019;21(7):59. | Review of existing literature, relevant for further screening |
| 145 | Christie HL, Bartels SL, Boots LMM, Tange HJ, Verhey FJJ, de Vugt ME. A systematic review on the implementation of eHealth interventions for informal caregivers of people with dementia. Internet Interv. 2018;13:51-9. | Review of existing literature, relevant for further screening |
| 146 | Christie HL, Martin JL, Connor J, Tange HJ, Verhey FRJ, de Vugt ME, et al. eHealth interventions to support caregivers of people with dementia may be proven effective, but are they implementation-ready? Internet Interv. 2019;18:100260. | Review of existing literature, relevant for further screening |
| 147 | Coumoundouros C, Mårtensson E, Ferraris G, Zuidberg JM, von Essen L, Sanderman R, et al. Implementation of e-Mental Health Interventions for Informal Caregivers of Adults With Chronic Diseases: Mixed Methods Systematic Review With a Qualitative Comparative Analysis and Thematic Synthesis. JMIR Ment Health. 2022;9(11):e41891. | Review of existing literature, relevant for further screening |
| 148 | Gonella S, Mitchell G, Bavelaar L, Conti A, Vanalli M, Basso I, et al. Interventions to support family caregivers of people with advanced dementia at the end of life in nursing homes: A mixed-methods systematic review. Palliat Med. 2022;36(2):268-91. | Review of existing literature, relevant for further screening |
| 149 | Graven LJ, Glueckauf RL, Regal RA, Merbitz NK, Lustria MLA, James BA. Telehealth Interventions for Family Caregivers of Persons with Chronic Health Conditions: A Systematic Review of Randomized Controlled Trials. Int J Telemed Appl. 2021;2021:3518050. | Review of existing literature, relevant for further screening |
| 150 | Han A. Effects of Mindfulness-Based Interventions on Depressive Symptoms, Anxiety, Stress, and Quality of Life in Family Caregivers of Persons Living with Dementia: A Systematic Review and Meta-analysis. Res Aging. 2022;44(7-8):494-509. | Review of existing literature, relevant for further screening |
| 151 | He J, Wang J, Zhong H, Guan C. The Effectiveness of Multi-Component Interventions on the Positive and Negative Aspects of Well-Being among Informal Caregivers of People with Dementia: A Systematic Review and Meta-Analysis. Int J Environ Res Public Health. 2022;19(12). | Review of existing literature, relevant for further screening |
| 152 | Hui Z, Yang C, Lee DTF. Interventions for Family Members After Long-Term Care Placement of a Relative With Dementia: A Systematic Review and Meta-Analysis. Res Gerontol Nurs. 2021;14(1):43-52. | Review of existing literature, relevant for further screening |
| 153 | Irons JY, Garip G, Cross AJ, Sheffield D, Bird J. An integrative systematic review of creative arts interventions for older informal caregivers of people with neurological conditions. PLoS ONE. 2020;15(12):e0243461. | Review of existing literature, relevant for further screening |
| 154 | Klimova B, Valis M, Kuca K, Masopust J. E-learning as valuable caregivers' support for people with dementia - A systematic review. BMC Health Serv Res. 2019;19(1):781. | Review of existing literature, relevant for further screening |
| 155 | Lee E. Do Technology-Based Support Groups Reduce Care Burden Among Dementia Caregivers? A Review. J Evid Inf Soc Work. 2015;12(5):474-87. | Review of existing literature, relevant for further screening |
| 156 | Liu X, Wang Y, Wang S. The efficacy of psychological interventions for depressed primary caregivers of patients with Alzheimer's disease: A systematic review and meta-analysis. J Nurs Scholarsh. 2022;54(3):355-66. | Review of existing literature, relevant for further screening |
| 157 | Meng X, Su J, Li H, Ma D, Zhao Y, Li Y, et al. Effectiveness of caregiver non-pharmacological interventions for behavioural and psychological symptoms of dementia: An updated meta-analysis. Ageing Res Rev. 2021;71:101448. | Review of existing literature, relevant for further screening |
| 158 | Müller C, Lautenschläger S, Meyer G, Stephan A. Interventions to support people with dementia and their caregivers during the transition from home care to nursing home care: A systematic review. Int J Nurs Stud. 2017;71:139-52. | Review of existing literature, relevant for further screening |
| 159 | Murfield J, Moyle W, O'Donovan A. Mindfulness- and compassion-based interventions for family carers of older adults: A scoping review. Int J Nurs Stud. 2021;116:103495. | Review of existing literature, relevant for further screening |
| 160 | Ottaviani AC, Monteiro DQ, Oliveira D, Gratão ACM, Jacinto AF, Campos CRF, et al. Usability and acceptability of internet-based interventions for family carers of people living with dementia: systematic review. Aging Ment Health. 2022;26(10):1922-32. | Review of existing literature, relevant for further screening |
| 161 | Piersol CV, Canton K, Connor SE, Giller I, Lipman S, Sager S. Effectiveness of Interventions for Caregivers of People With Alzheimer's Disease and Related Major Neurocognitive Disorders: A Systematic Review. Am J Occup Ther. 2017;71(5):7105180020p1-p10. | Review of existing literature, relevant for further screening |
| 162 | Raj SE, Mackintosh S, Fryer C, Stanley M. Home-Based Occupational Therapy for Adults With Dementia and Their Informal Caregivers: A Systematic Review. Am J Occup Ther. 2021;75(1):7501205060p1-p27. | Review of existing literature, relevant for further screening |
| 163 | Ramachandran M, Bangera K, Anita Dsouza S, Belchior P. A scoping review of family-centered interventions in dementia care. Dementia (London). 2023;22(2):405-38. | Review of existing literature, relevant for further screening |
| 164 | Richardson A, Pedley G, Pelone F, Akhtar F, Chang J, Muleya W, et al. Psychosocial interventions for people with young onset dementia and their carers: a systematic review. Int Psychogeriatr. 2016;28(9):1441-54. | Review of existing literature, relevant for further screening |
| 165 | Ruggiano N, Brown EL, Roberts L, Framil Suarez CV, Luo Y, Hao Z, et al. Chatbots to Support People With Dementia and Their Caregivers: Systematic Review of Functions and Quality. J Med Internet Res. 2021;23(6):e25006. | Review of existing literature, relevant for further screening |
| 166 | Saragih ID, Tonapa SI, Lin CJ, Lee BO. Effects of case management intervention for people with dementia and their carers: A systematic review and meta-analysis of experimental studies. Int J Nurs Stud. 2021;121:104012. | Review of existing literature, relevant for further screening |
| 167 | Saragih ID, Tonapa SI, Porta CM, Lee BO. Effects of telehealth intervention for people with dementia and their carers: A systematic review and meta-analysis of randomized controlled studies. J Nurs Scholarsh. 2022;54(6):704-19. | Review of existing literature, relevant for further screening |
| 168 | Scott JL, Dawkins S, Quinn MG, Sanderson K, Elliott KE, Stirling C, et al. Caring for the carer: a systematic review of pure technology-based cognitive behavioral therapy (TB-CBT) interventions for dementia carers. Aging Ment Health. 2016;20(8):793-803. | Review of existing literature, relevant for further screening |
| 169 | Shim M, Tilley JL, Im S, Price K, Gonzalez A. A Systematic Review of Mindfulness-Based Interventions for Patients with Mild Cognitive Impairment or Dementia and Caregivers. J Geriatr Psychiatry Neurol. 2021;34(6):528-54. | Review of existing literature, relevant for further screening |
| 170 | Sin J, Henderson C, Spain D, Cornelius V, Chen T, Gillard S. eHealth interventions for family carers of people with long term illness: A promising approach? Clin Psychol Rev. 2018;60:109-25. | Review of existing literature, relevant for further screening |
| 171 | Sitges-Maciá E, Bonete-López B, Sánchez-Cabaco A, Oltra-Cucarella J. Effects of e-Health Training and Social Support Interventions for Informal Caregivers of People with Dementia-A Narrative Review. Int J Environ Res Public Health. 2021;18(15). | Review of existing literature, relevant for further screening |
| 172 | Van Mierlo LD, Meiland FJ, Van der Roest HG, Dröes RM. Personalised caregiver support: effectiveness of psychosocial interventions in subgroups of caregivers of people with dementia. Int J Geriatr Psychiatry. 2012;27(1):1-14. | Review of existing literature, relevant for further screening |
| 173 | Waller A, Dilworth S, Mansfield E, Sanson-Fisher R. Computer and telephone delivered interventions to support caregivers of people with dementia: a systematic review of research output and quality. BMC Geriatr. 2017;17(1):265. | Review of existing literature, relevant for further screening |
| 174 | Wasilewski MB, Stinson JN, Cameron JI. Web-based health interventions for family caregivers of elderly individuals: A Scoping Review. Int J Med Inform. 2017;103:109-38. | Review of existing literature, relevant for further screening |
| 175 | Wen Y, Xing Y, Ding Y, Xu W, Wang X. Challenges of conducting of online educational programs for family caregivers of people with dementia living at home: An integrative review. Int J Nurs Sci. 2023;10(1):121-8. | Review of existing literature, relevant for further screening |
| 176 | Ying J, Wang Y, Zhang M, Wang S, Shi Y, Li H, et al. Effect of multicomponent interventions on competence of family caregivers of people with dementia: A systematic review. J Clin Nurs. 2018;27(9-10):1744-58. | Review of existing literature, relevant for further screening |
| 177 | Yousaf K, Mehmood Z, Saba T, Rehman A, Munshi AM, Alharbey R, et al. Mobile-Health Applications for the Efficient Delivery of Health Care Facility to People with Dementia (PwD) and Support to Their Carers: A Survey. Biomed Res Int. 2019;2019:7151475. | Review of existing literature, relevant for further screening |
| 178 | Yu Y, Xiao L, Ullah S, Meyer C, Wang J, Pot AM, et al. The effectiveness of internet-based psychoeducation programs for caregivers of people living with dementia: a systematic review and meta-analysis. Aging Ment Health. 2023:1-17. | Review of existing literature, relevant for further screening |
| 179 | Zhu A, Cao W, Zhou Y, Xie A, Cheng Y, Chu SF. Tele-Health Intervention for Carers of Dementia Patients-A Systematic Review and Meta-Analysis of Randomized Controlled Trials. Front Aging Neurosci. 2021;13:612404. | Review of existing literature, relevant for further screening |
| 180 | Fialho PP, Köenig AM, Santos MD, Barbosa MT, Caramelli P. Positive effects of a cognitive-behavioral intervention program for family caregivers of demented elderly. Arq Neuropsiquiatr. 2012;70(10):786-92. | Non-RCT |
| 181 | Miller PA, Butin D. The role of occupational therapy in dementia-C.O.P.E. (Caregiver options for practical experiences). Int J Geriatr Psychiatry. 2000;15(1):86-9. | Non-RCT |
| 182 | Steuer JL, Clark EO. Family support groups within a research project on dementia. Clin Gerontol. 1982;1(1):87-95. | Non-RCT |
| 183 | Mollaei P, Momtaz YA, Taheri-Tanjani P. The Effect of Cyberspace-Based Education on the Well-Being of Caregivers of Elderly People with Dementia. Curr Aging Sci. 2021;14(2):105-11. | Non-RCT |
| 184 | Harris ML, Van Houtven CH, Hastings SN. Development of a Home-Based Stress Management Toolkit for Dementia Caring Dyads: Protocol for A Pilot Intervention Development and Feasibility Study. JMIR Res Protoc. 2022. | Protocol, no intervention results |
| 185 | Than TN, Nguyen TT, Nguyen TC, Vu LT, Vo PT, K TT, et al. Smartphone app-based intervention for reducing stress, depression, and anxiety in caregivers of people with dementia in Vietnam: Study protocol for a pilot randomized controlled trial. Digit Health. 2023;9:20552076231163786. | Protocol, no intervention results |
| 186 | Levinson AJ, Bousfield J, Douglas W, Ayers S, Sztramko R. A Novel Educational Prescription Web-Based Application to Support Education for Caregivers of People Living With Dementia: Development and Usability Study With Clinicians. JMIR Hum Factors. 2020;7(4):e23904. | Development process/adaption/validation of a tool/program, no outcomes of intervention |
| 187 | Masterson-Algar P, Egan K, Flynn G, Hughes G, Spector A, Stott J, et al. iSupport for Young Carers: An Adaptation of an e-Health Intervention for Young Dementia Carers. Int J Environ Res Public Health. 2022;20(1). | Development process/adaption/validation of a tool/program, no outcomes of intervention |
| 188 | Hain DJ. Home-based biobehavioural intervention reduces dependence, increases engagement of patients with dementia in the short term and improves care giver well-being and confidence. Evid Based Nurs. 2011;14(2):39-40. | Commentary, not intervention |
| 189 | Knapp M, Shehaj X, Wong G. Digital interventions for people with dementia and carers: effective, cost-effective and equitable? Neurodegener Dis Manag. 2022;12(5):215-9. | Overview of services or theory, not intervention |
| 190 | Deck R, Bussmann ML, Simons J, Al-Hashimy S, Stark M. [Rehabilitation Program for Persons with Relatives Suffering from Dementia: Results of a Longitudinal Observational Study]. Rehabilitation (Stuttg). 2019;58(2):112-20. | Unable to obtain English or Vietnamese FT |
| 191 | González-Fraile E, Ballesteros J, Rueda JR, Santos-Zorrozúa B, Solà I, McCleery J. Remotely delivered information, training and support for informal caregivers of people with dementia. Cochrane Database of Systematic Reviews. 2021;2021(1). | Review of existing literature, relevant for further screening |
| 192 | Rathnayake S, Moyle W, Jones C, Calleja P. mHealth applications as an educational and supportive resource for family carers of people with dementia: An integrative review. Dementia. 2019;18(7-8):3091-112. | Review of existing literature, relevant for further screening |
| 193 | Cheng ST, Li KK, Losada A, Zhang F, Au A, Thompson LW, et al. The effectiveness of nonpharmacological interventions for informal dementia caregivers: An updated systematic review and meta-analysis. Psychol Aging. 2020;35(1):55-77. | Review of existing literature, relevant for further screening |
| 194 | Clarkson P, Hughes J, Roe B, Giebel CM, Jolley D, Poland F, et al. Systematic review: Effective home support in dementia care, components and impacts – Stage 2, effectiveness of home support interventions. J Adv Nurs. 2018;74(3):507-27. | Review of existing literature, relevant for further screening |
| 195 | Jensen M, Agbata IN, Canavan M, McCarthy G. Effectiveness of educational interventions for informal caregivers of individuals with dementia residing in the community: Systematic review and meta-analysis of randomised controlled trials. International Journal of Geriatric Psychiatry. 2015;30(2):130-43. | Review of existing literature, relevant for further screening |
| 196 | Vandepitte S, Van Den Noortgate N, Putman K, Verhaeghe S, Faes K, Annemans L. Effectiveness of Supporting Informal Caregivers of People with Dementia: A Systematic Review of Randomized and Non-Randomized Controlled Trials. J Alzheimer's Dis. 2016;52(3):929-65. | Review of existing literature, relevant for further screening |
| 197 | Verreault P, Turcotte V, Ouellet MC, Robichaud LA, Hudon C. Efficacy of cognitive-behavioural therapy interventions on reducing burden for caregivers of older adults with a neurocognitive disorder: a systematic review and meta-analysis. Cogn Behav Ther. 2021;50(1):19-46. | Review of existing literature, relevant for further screening |
| 198 | Wiegelmann H, Speller S, Verhaert LM, Schirra-Weirich L, Wolf-Ostermann K. Psychosocial interventions to support the mental health of informal caregivers of persons living with dementia – a systematic literature review. BMC Geriatrics. 2021;21(1). | Review of existing literature, relevant for further screening |
| 199 | Williams F, Moghaddam N, Ramsden S, De Boos D. Interventions for reducing levels of burden amongst informal carers of persons with dementia in the community. A systematic review and meta-analysis of randomised controlled trials. Aging and Mental Health. 2019;23(12):1629-42. | Review of existing literature, relevant for further screening |
| 200 | Bui LK, Park M, Giap TTT. eHealth interventions for the informal caregivers of people with dementia: A systematic review of systematic reviews. Geriatric Nursing. 2022;48:199-209. | Review of existing literature, relevant for further screening |
| 201 | Sun Y, Ji M, Leng M, Li X, Zhang X, Wang Z. Comparative efficacy of 11 non-pharmacological interventions on depression, anxiety, quality of life, and caregiver burden for informal caregivers of people with dementia: A systematic review and network meta-analysis. International Journal of Nursing Studies. 2022;129. | Review of existing literature, relevant for further screening |
| 202 | Wu KC, Su Y, Chu F, Chen AT, Zaslavsky O. Behavioral Change Factors and Retention in Web-Based Interventions for Informal Caregivers of People Living With Dementia: Scoping Review. Journal of Medical Internet Research. 2022;24(7). | Review of existing literature, relevant for further screening |
| 203 | Caprioli T, Mason S, Tetlow H, Reilly S, Giebel C. Exploring the views and the use of information and communication technologies to access post-diagnostic support by people living with dementia and unpaid carers: a systematic review. Aging and Mental Health. 2023. | Review of existing literature, relevant for further screening |
| 204 | Fossey J, Charlesworth G, Fowler JA, Frangou E, Pimm TJ, Dent J, et al. Online Education and Cognitive Behavior Therapy Improve Dementia Caregivers' Mental Health: A Randomized Trial. Journal of the American Medical Directors Association. 2020. | Non-LMIC |
| 205 | Graff MJL, Vernooij-Dassen MJM, Thijssen M, Dekker J, Hoefnagels WHL, Olderikkert MGM. Effects of community occupational therapy on quality of life, mood, and health status in dementia patients and their caregivers: A randomized controlled trial. J Gerontol Ser A Biol Sci Med Sci. 2007;62(9):1002-9. | Non-LMIC |
| 206 | van Wezel N, van der Heide I, Devillé WL, Kayan Acun E, Meerveld JHCM, Spreeuwenberg P, et al. Effects of an educational peer-group intervention on knowledge about dementia among family caregivers with a Turkish or Moroccan immigrant background: A cluster randomised controlled trial. Patient Education and Counseling. 2020. | Non-LMIC |
| 207 | Jain FA, Chernyak SV, Nickerson LD, Morgan S, Schafer R, Mischoulon D, et al. Four-Week Mentalizing Imagery Therapy for Family Dementia Caregivers: A Randomized Controlled Trial with Neural Circuit Changes. Psychother Psychosom. 2022;91(3):180-9. | Non-LMIC |
| 208 | Kor PPK, Liu JYW, Chien WT. Effects of a modified mindfulness-based cognitive therapy for family caregivers of people with dementia: A randomized clinical trial. Gerontologist. 2021;61(6):977-90. | Non-LMIC |
| 209 | Li B, Ho RTH, Leung WYV, Tsang KT. Including formal and informal caregivers in the development of Play Intervention for Dementia: a qualitative study. BMC Geriatrics. 2022;22(1). | Non-LMIC |
| 210 | Osté J, Dröes RM. A meeting centre for Surinam people with dementia and their informal carers; development and implementation of culture specific combined support. Tijdschr Gerontol Geriatr. 2005;36(6):232-42. | Not available in English |
| 211 | Sözeri Varma G, Kalkan Oğuzhanoğlu N, Çulha Ateşci F, Karagöz N, Apa F. Psychodramatic group therapy on alzheimer patients’ caregiver relatives. Anadolu Psikiyatr Derg. 2018;19(6):567-76. | Not available in English |
| 212 | Gok Ugur H, Orak OS, Yaman Aktas Y, Enginyurt O, Saglambilen O. Effects of music therapy on the care burden of in-home caregivers and physiological parameters of their in-home dementia patients: A randomized controlled trial. Comp Med Res. 2019;26(1):22-30. | Intervention not delivered to the caregivers |
| 213 | Maneewat T, Lertmaharit S, Tangwongchai S, Phansuea P. Development of multi-component counseling program for enhancing resilience among Thai caregivers of older persons with dementia. J Health Res. 2022;36(5):813-22. | Non-RCT — quasi-experimental |
| 214 | Au A, Lai DWL, Biggs S, Cheng ST, Haapala-Biggs I, Chow A, et al. Perspective-Taking Interventions for Intergenerational Caregivers of Alzheimer's Diseases: A Randomized Controlled Trial. Res Soc Work Pract. 2020;30(3):306-19. | Non-LMIC |
| 215 | Au A, Yip HM, Lai S, Ngai S, Cheng ST, Losada A, et al. Telephone-based behavioral activation intervention for dementia family caregivers: Outcomes and mediation effect of a randomized controlled trial. Patient Education and Counseling. 2019;102(11):2049-59. | Non-LMIC |
| 216 | Cheng ST, Chan WC, Lam LCW. Long-Term Outcomes of the Benefit-Finding Group Intervention for Alzheimer Family Caregivers: A Cluster-Randomized Double-Blind Controlled Trial. American Journal of Geriatric Psychiatry. 2019;27(9):984-94. | Non-LMIC |
| 217 | Cheng ST, Fung HH, Chan WC, Lam LCW. Short-Term Effects of a Gain-Focused Reappraisal Intervention for Dementia Caregivers: A Double-Blind Cluster-Randomized Controlled Trial. American Journal of Geriatric Psychiatry. 2016;24(9):740-50. | Non-LMIC |
| 218 | Cheung DSK, Kor PPK, Jones C, Davies N, Moyle W, Chien WT, et al. The Use of Modified Mindfulness-Based Stress Reduction and Mindfulness-Based Cognitive Therapy Program for Family Caregivers of People Living with Dementia: A Feasibility Study. Asian Nursing Research. 2020;14(4):221-30. | Non-LMIC |
| 219 | Hsiao HT, Chang CC, Chen NC, Chiu HC, Huang CW, Lee FP, et al. Effects of a dementia dietary educational program on nutritional knowledge and healthy eating behavior of family caregivers. Educational Gerontology. 2020;46(5):270-83. | Non-LMIC |
| 220 | Kor PPK, Liu JYW, Chien WT. Effects of a modified mindfulness-based cognitive therapy for family caregivers of people with dementia: A pilot randomized controlled trial. International Journal of Nursing Studies. 2019;98:107-17. | Non-LMIC |
| 221 | Kwok T, Au A, Wong B, Ip I, Mak V, Ho F. Effectiveness of online cognitive behavioral therapy on family caregivers of people with dementia. Clin Interv Aging. 2014;9:631-6. | Non-LMIC |
| 222 | Lai CKY, Lai DLL, Ho JSC, Wong KKY, Cheung DSK. Interdisciplinary collaboration in the use of a music-with-movement intervention to promote the wellbeing of people with dementia and their families: Development of an evidence-based intervention protocol. Nursing & Health Sciences. 2016;18(1):79-84. | Non-LMIC |
| 223 | Lai DLL, Lai CKY. A case study on a home-based caregiver-delivered music-with-movement intervention for people with early dementia. SAGE Open Med Case Rep. 2017;5:4. | Non-LMIC |
| 224 | Law W, Kwok TCY. Impacts of a multicomponent intervention programme on neuropsychiatric symptoms in people with dementia and psychological health of caregivers: A feasibility pilot study. International Journal of Geriatric Psychiatry. 2019;34(12):1765-75. | Non-LMIC |
| 225 | Mbakile-Mahlanza L, van der Ploeg ES, Busija L, Camp C, Walker H, O'Connor DW. A cluster-randomized crossover trial of Montessori activities delivered by family carers to nursing home residents with behavioral and psychological symptoms of dementia. International Psychogeriatrics. 2020;32(3):347-58. | Non-LMIC |
| 226 | Yu DSF, Li PWC, Zhang F, Cheng ST, Ng TK, Judge KS. The effects of a dyadic strength-based empowerment program on the health outcomes of people with mild cognitive impairment and their family caregivers: a randomized controlled trial. Clin Interv Aging. 2019;14:1705-17. | Non-LMIC |
| 227 | Fredriksen-Goldsen K, Teri L, Kim HJ, La Fazia D, McKenzie G, Petros R, et al. Design and development of the first randomized controlled trial of an intervention (IDEA) for sexual and gender minority older adults living with dementia and care partners. Contemporary Clinical Trials. 2023;128:9. | Non-LMIC |
| 228 | Lee JA, Aqajari SAH, Ju E, Kehoe P, Gibbs L, Rahmani A. HOME-VISIT INTERVENTION TO REDUCE STRESS OF UNDERSERVED FAMILY CAREGIVERS FOR PERSONS WITH DEMENTIA. Innov Aging. 2021;5:152 | Non-LMIC |
| 229 | Maneewat T, Lertmaharit S, Tangwongchai S, Phansuea P. Development of multi-component counseling program for enhancing resilience among Thai caregivers of older persons with dementia. J Health Res. 2022;36(5):813-22. | Non-randomised |
| 230 | Lertkratoke S, Amnatsatsue K, Kerdmongkol P, Nanthamongkolchai S. Effectiveness of Thai Integrated Care Program for Older Adults with Dementia in the Community: A Quasi-experimental Study. Pacific Rim International Journal of Nursing Research. 2021;25(4):510-24. | Non-RCT |
| 231 | Balli FN, Unsal P, Halil MG, Dogu BB, Cankurtaran M, Demirkan K. Effect of clinical pharmacists' interventions on dementia treatment adherence and caregivers' knowledge. Geriatrics & Gerontology International.6. | Non-controlled trial |
| 232 | de Oliveira TI, Maziero BR, Buriol D, da Rosa PH, Ilha S. QUALITY OF LIFE OF FAMILY MEMBERS/CAREGIVERS OF ELDERLY PEOPLE BEARING ALZHEIMER'S DISEASE: SUPPORT GROUP CONTRIBUTIONS. Rev Pesqui-Cuid Fundam Online. 2020;12:827-32. | Non-controlled trial |
| 233 | Han J, Guo GF, Hong L. Impact of professionally facilitated peer support for family carers of people with dementia in a WeChat virtual community. J Telemed Telecare. 2020:9. | Non-controlled trial |
| 234 | Kucukguclu O, Soylemez BA, Yener G, Isik AT. The effects of support groups on dementia caregivers: A mixed method study. Geriatric Nursing. 2018;39(2):151-6. | Non-controlled trial |
| 235 | Kuzu N, Beser N, Zencir M, Sahiner T, Nesrin E, Ahmet E, et al. Effects of a comprehensive educational program on quality of life and emotional issues of dementia patient caregivers. Geriatric Nursing. 2005;26(6):378-86. | Non-controlled trial |
| 236 | Santos RL, de Sousa MFB, Arcoverde C, Dourado MCN. Efficacy of a psychoeducational group with caregivers of patients with dementia. Rev Psiquiatr Clin. 2013;40(4):162-4. | Non-controlled trial |
| 237 | Zakaria R, Ab Razak A. THE EFFECTIVENESS OF A CULTURAL-BASED SUPPORT GROUP FOR MALAY DEMENTIA CAREGIVERS IN KELANTAN, MALAYSIA: A PRE-POST INTERVENTION STUDY. ASEAN J Psychiatr. 2017;18(1):20-30. | Non-controlled trial |
| 238 | Cordoba AMC, Poches DKP, Ruiz AL. Implementation of the Psychological Intervention program in Resilience for Informal Caregivers of Patients with Alzheimer's Dementia PIRCA. Univ Psychol. 2017;16(2):12. | Not available in English |
| 239 | Hosseini MA, Mohammadzaheri S, Khoshkenab MF, Shahbolaghi FM, Soltani PR, Mohseni MS. Effect of Mindfulness Program on Caregivers' Strain on Alzheimer's Disease Caregivers. Salmand. 2016;11(3):448-54. | Not available in English |
| 240 | Mahmoodi M, Khani PM, Banab BG, Bagheri F. Effectiveness of Group Cognitive-Behavioral Therapy on Strategies for Coping with Stress of Family Caregivers of Patients with Alzheimer's Disease. Salmand. 2016;11(1):190-200. | Not available in English |
| 241 | Rojas TAS, Riano LJR. CUIDO COMUNICANDO: DESIGN AND VALIDATION OF A COMMUNICATIVE SKILLS PROGRAM FOR CAREGIVERS OF PEOPLE WITH ALZHEIMER'S DISEASE. Cult Cuid. 2020;24(58):304-14. | Not available in English |
| 242 | Sozeri Varma G, Kalkan Oguzhanoglu N, Culha Atesci F, Karagoz N, Apa F. Psychodramatic group therapy on Alzheimer patients' caregiver relatives. Anadolu Psikiyatri Dergisi-Anatolian Journal of Psychiatry. 2018;19(6):567-76. | Not available in English |
| 243 | Chen CY, Huang YY, Liu CX, Xu Y, Zheng LY, Li J. Effects of an Interdisciplinary Care Team on the Management of Alzheimer's Disease in China. J Gerontol Nurs. 2019;45(5):39-45. | Intervention not delivered to the caregivers |
| 244 | Kolykhalov IV, Rassadina GA, Gavrilova SI, Gerasimov NP. Cholinergic therapy of Alzheimer's disease and its effect on health and quality of life of caregivers to the patients. Z Nevrol Psikhiatrii Im S S Korsakova. 2010;110(5):33-8. | Intervention not delivered to the caregivers |
| 245 | Ma MY, Wang JT, Zhang BA. Changes in psychological states of caregivers of patients with moderate or severe Alzheimer's disease following Memantine therapy. Life Sci J. 2012;9(3):155-8. | Intervention not delivered to the caregivers |
| 246 | Stella F, Canonici AP, Gobbi S, Santos-Galduroz RF, Cacao JD, Gobbi LTB. Attenuation of neuropsychiatric symptoms and caregiver burden in Alzheimer's disease by motor intervention: a controlled trial. Clinics. 2011;66(8):1353-60. | Intervention not delivered to the caregivers |
| 247 | Aboulafia-Brakha T, Suchecki D, Gouveia-Paulino F, Nitrini R, Ptak R. Cognitive-behavioural group therapy improves a psychophysiological marker of stress in caregivers of patients with Alzheimer's disease. Aging & Mental Health. 2014;18(6):801-8. | Non-randomised |
| 248 | Ghezelsefloo M, Saadati N, Yousefi Z, Zamanpour M. Study the Effect of Resilience Training on Reducing Stress and Communication Problems in the Primary Caregivers of the Elderly With Alzheimer Disease. Salmand. 2019;14(3):284-96. | Non-randomised |
| 249 | Villareal-Reyna MD, Salazar-Gonzalez BC, Cruz-Quevedo JE, Carrillo-Cervantes AL, Champion JD. Outcomes of Interventions for Alzheimer's Family Caregivers in Mexico. West J Nurs Res. 2012;34(7):973-90. | Non-randomised |
| 250 | Li YN, Hu LY, Mao XE, Shen YJ, Xue HP, Hou P, et al. Health literacy, social support, and care ability for caregivers of dementia patients: Structural equation modeling. Geriatric Nursing. 2020;41(5):600-7. | Exploring factors of needs/unmet needs rather than intervention |
| 251 | Rong XS, Wu F, Tang DL, Zhao YX, Guo QH, He F, et al. Development of a self-management support program for caregivers of relatives with dementia in Shanghai. Geriatric Nursing. 2020;41(2):98-104. | Exploring factors of needs/unmet needs rather than intervention |
| 252 | Rote S, Angel J, Vega W. CARE IN CONTEXT: DEMENTIA SUPPORT IN MEXICO AND THE UNITED STATES. Innov Aging. 2021;5:476. | Exploring factors of needs/unmet needs rather than intervention |
| 253 | Rao GP, Sivakumar PT, Srivastava S, Sidana RC. Cognitive Therapy and Family Intervention for Patients with Dementia and Psychosis. Indian Journal of Psychiatry. 2020;62:8183-91. | Article/guidelines, not intervention |
| 254 | Sukhdeve N, Singh S. ASSESS THE EFFECTIVENESS OF PLANNED TEACHING ON KNOWLEDGE REGARDING EARLY WARNING SIGNS AND MANAGEMENT OF ALZHEIMER'S DISEASE AMONG CARE GIVERS OF ELDERLY CLIENT. Int J Mod Agric. 2020;9(3):148-53. | Protocol, no intervention results (followed up 23 Jan 2023 — non RCT) |
| 255 | Canonici AP, de Andrade LP, Gobbi S, Santos-Galduroz RF, Gobbi LTB, Stella F. Functional dependence and caregiver burden in Alzheimer's disease: a controlled trial on the benefits of motor intervention. Psychogeriatrics. 2012;12(3):186-92. | Intervention not focused on caregivers |
| 256 | Pan YQ, Chen RY, Yang DL. The Role of Mutuality and Coping in a Nurse-Led Cognitive Behavioral Intervention on Depressive Symptoms Among Dementia Caregivers. Res Gerontol Nurs. 2019;12(1):44-55. | Duplicate (not picked up due to way exported to EndNote and different screening authors for each version – different journals, same outcome measures reported) |
| 257 | Cheng ST, Zhang F. A comprehensive meta-review of systematic reviews and meta-analyses on nonpharmacological interventions for informal dementia caregivers. Bmc Geriatrics. 2020;20(1):24. | Review of existing literature, relevant for further screening |
| 258 | Sari YM, Hill KD, Lee DCA, Burton E. Effectiveness of exercise programmes in improving physical function and reducing behavioural symptoms of community living older adults with dementia living in Asia, and impact on their informal carers: A systematic review and meta-analysis. Hong Kong Physiother J.15. | Review of existing literature, relevant for further screening |
| 259 | Srikanth P, Antony S, Manjunatha S, Thirumoorthy A. Importance of Day-Care Centers in Dementia Care: A case Study From India. Indian Journal of Community Medicine. 2021;46(2):321-2. | Case study |
| 260 | Akkerman RL, Ostwald SK. Reducing anxiety in Alzheimer's disease family caregivers: The effectiveness of a nine-week cognitive-behavioral intervention. American Journal of Alzheimer's Disease & Other Dementias®. 2004;19(2):117-23. | Non-LMIC |
| 261 | Au A, Gallagher-Thompson D, Wong M-K, Leung J, Chan W-C, Chan CC, et al. Behavioral activation for dementia caregivers: scheduling pleasant events and enhancing communications. Clin Interv Aging. 2015:611-9. | Non-LMIC |
| 262 | Bass DM, Clark PA, Looman WJ, McCarthy CA, Eckert S. The Cleveland Alzheimer's Managed Care Demonstration: Outcomes After 12 Months of Implementation. The Gerontologist. 2003;43(1):73-85. | Non-LMIC |
| 263 | Beauchamp N, Irvine AB, Seeley J, Johnson B. Worksite-Based Internet Multimedia Program for Family Caregivers of Persons With Dementia. The Gerontologist. 2005;45(6):793-801. | Non-LMIC |
| 264 | Brennan PF, Moore SM, Smyth KA. The effects of a special computer network on caregivers of persons with Alzheimer's disease. Nurs Res. 1995;44(3):166-72. | Non-LMIC |
| 265 | Buckwalter KC, Gerdner L, Kohout F, Hall GR, Kelly A, Richards B, et al. A nursing intervention to decrease depression in family caregivers of persons with dementia. Archives of Psychiatric Nursing. 1999;13(2):80-8. | Non-LMIC |
| 266 | Chang BL. Cognitive-behavioral intervention for homebound caregivers of persons with dementia. Nursing Research. 1999;48(3):173-82. | Non-LMIC |
| 267 | Connell CM, Janevic MR. Effects of a telephone-based exercise intervention for dementia caregiving wives: A randomized controlled trial. Journal of Applied Gerontology. 2009;28(2):171-94. | Non-LMIC |
| 268 | Coon DW, Thompson L, Steffen A, Sorocco K, Gallagher-Thompson D. Anger and Depression Management: Psychoeducational Skill Training Interventions for Women Caregivers of a Relative With Dementia. The Gerontologist. 2003;43(5):678-89. | Non-LMIC |
| 269 | Czaja SJ, Loewenstein D, Schulz R, Nair SN, Perdomo D. A Videophone Psychosocial Intervention for Dementia Caregivers. The American Journal of Geriatric Psychiatry. 2013;21(11):1071-81. | Non-LMIC |
| 270 | Davis B, Shehab M, Shenk D, Nies M. E-mobile pilot for community-based dementia caregivers identifies desire for security. Gerontechnology. 2015;13(3):332-6. | Non-LMIC |
| 271 | Davis LL, Burgio LD, Buckwalter KC, Weaver M. A comparison of in-home and telephone-based skill training interventions with caregivers of persons with Dementia. Journal of Mental Health and Aging. 2004. | Non-LMIC |
| 272 | Ducharme F, Dubé V, Lévesque L, Saulnier D, Giroux F. An online stress management training program as a supportive nursing intervention for family caregivers of an elderly person. Canadian Journal of Nursing Informatics. 2011;6(2):1-19. | Non-LMIC |
| 273 | Farran CJ, Gilley DW, McCann JJ, Bienias JL, Lindeman DA, Evans DA. Psychosocial interventions to reduce depressive symptoms of dementia caregivers: A randomized clinical trial comparing two approaches. Journal of Mental Health and Aging. 2004. | Non-LMIC |
| 274 | Farran CJ, Paun O, Cothran F, Etkin CD, Rajan KB, Eisenstein A, et al. Impact of an individualized physical activity intervention on improving mental health outcomes in family caregivers of persons with dementia: A randomized controlled trial. AIMS medical science. 2016;3(1):15. | Non-LMIC |
| 275 | Fowler C, Haney T, Rutledge CM. An interprofessional virtual healthcare neighborhood for caregivers of elderly with dementia. The Journal for Nurse Practitioners. 2014;10(10):829-34. | Non-LMIC |
| 276 | Gant JR, Steffen AM, Lauderdale SA. Comparative outcomes of two distance-based interventions for male caregivers of family members with dementia. American Journal of Alzheimer's Disease and other Dementias. 2007;22(2):120-8. | Non-LMIC |
| 277 | Hicken BL, Daniel C, Luptak M, Grant M, Kilian S, Rupper RW. Supporting caregivers of rural veterans electronically (SCORE). The Journal of Rural Health. 2017;33(3):305-13. | Non-LMIC |
| 278 | Hopwood J, Walker N, McDonagh L, Rait G, Walters K, Iliffe S, et al. Internet-Based Interventions Aimed at Supporting Family Caregivers of People With Dementia: Systematic Review. J Med Internet Res. 2018;20(6):e216. | Non-LMIC |
| 279 | King AC, Baumann K, O'Sullivan P, Wilcox S, Castro C. Effects of moderate-intensity exercise on physiological, behavioral, and emotional responses to family caregiving: a randomized controlled trial. The Journals of Gerontology Series A: Biological Sciences and Medical Sciences. 2002;57(1):M26-M36. | Non-LMIC |
| 280 | Ko JW. Alzheimer's disease and related disorders caregiver's acceptance of a web-based structured written emotional expression intervention [Ph.D.]. United States -- Iowa: The University of Iowa; 2011. | Non-LMIC |
| 281 | Lewis ML, Hobday JV, Hepburn KW. Internet-based program for dementia caregivers. Am J Alzheimers Dis Other Demen. 2010;25(8):674-9. | Non-LMIC |
| 282 | Liddle J, Smith-Conway ER, Baker R, Angwin AJ, Gallois C, Copland DA, et al. Memory and communication support strategies in dementia: Effect of a training program for informal caregivers. International Psychogeriatrics. 2012;24(12):1927-42. | Non-LMIC |
| 283 | Loi SM, Tropea J, Gaffy E, Panayiotou A, Capon H, Chiang J, et al. START-online: acceptability and feasibility of an online intervention for carers of people living with dementia. Pilot and Feasibility Studies. 2022;8(1):41. | Non-LMIC |
| 284 | Mahoney DF, Tarlow BJ, Jones RN. Effects of an automated telephone support system on caregiver burden and anxiety: findings from the REACH for TLC intervention study. The Gerontologist. 2003;43(4):556-67. | Non-LMIC |
| 285 | Martindale-Adams J, Nichols LO, Burns R, Graney MJ, Zuber J. A trial of dementia caregiver telephone support. Can J Nurs Res. 2013;45(4):30-48. | Non-LMIC |
| 286 | Marziali E, Garcia LJ. Dementia caregivers' responses to 2 Internet-based intervention programs. Am J Alzheimers Dis Other Demen. 2011;26(1):36-43. | Non-LMIC |
| 287 | Moskowitz JT, Cheung EO, Snowberg KE, Verstaen A, Merrilees J, Salsman JM, et al. Randomized controlled trial of a facilitated online positive emotion regulation intervention for dementia caregivers. Health Psychol. 2019;38(5):391-402. | Non-LMIC |
| 288 | Perin S, Lai R, Diehl-Schmid J, You E, Kurz A, Tensil M, et al. Online counselling for family carers of people with young onset dementia: The RHAPSODY-Plus pilot study. Digit Health. 2023;9:20552076231161962. | Non-LMIC |
| 289 | RM OD. Mindfulness‐Based Stress Reduction as an Intervention Among Family Caregivers of Persons With Neurocognitive Disorders: The University of Arizona; 2013. | Non-LMIC |
| 290 | Spalding-Wilson KN, Guzmán-Vélez E, Angelica J, Wiggs K, Savransky A, Tranel D. A novel two-day intervention reduces stress in caregivers of persons with dementia. Alzheimer's & Dementia: Translational Research & Clinical Interventions. 2018;4(1):450-60. | Non-LMIC |
| 291 | Steffen AM, Gant JR. A telehealth behavioral coaching intervention for neurocognitive disorder family carers. International Journal of Geriatric Psychiatry. 2016;31(2):195-203. | Non-LMIC |
| 292 | Strawn BD, Hester S, Brown WS. Telecare: A social support intervention for family caregivers of dementia victims. Clinical Gerontologist: The Journal of Aging and Mental Health. 1998;18(3):66-9. | Non-LMIC |
| 293 | Tremont G, Davis J, Papandonatos GD, Grover C, Ott BR, Fortinsky RH, et al. A telephone intervention for dementia caregivers: Background, design, and baseline characteristics. Contemporary Clinical Trials. 2013;36(2):338-47. | Non-LMIC |
| 294 | Tremont G, Davis JD, Bishop DS, Fortinsky RH. Telephone-Delivered Psychosocial Intervention Reduces Burden in Dementia Caregivers. Dementia (London). 2008;7(4):503-20. | Non-LMIC |
| 295 | Tremont G, Davis JD, Papandonatos GD, Ott BR, Fortinsky RH, Gozalo P, et al. Psychosocial telephone intervention for dementia caregivers: A randomized, controlled trial. Alzheimers Dement. 2015;11(5):541-8. | Non-LMIC |
| 296 | van der Roest HG, Meiland FJ, Jonker C, Dröes RM. User evaluation of the DEMentia-specific Digital Interactive Social Chart (DEM-DISC). A pilot study among informal carers on its impact, user friendliness and, usefulness. Aging Ment Health. 2010;14(4):461-70. | Non-LMIC |
| 297 | Wilz G, Schinköthe D, Soellner R. Goal attainment and treatment compliance in a cognitive-behavioral telephone intervention for family caregivers of persons with dementia. GeroPsych: The Journal of Gerontopsychology and Geriatric Psychiatry. 2011;24(3):115-25. | Non-LMIC |
| 298 | Wilz G, Soellner R. Evaluation of a Short-Term Telephone-Based Cognitive Behavioral Intervention for Dementia Family Caregivers. Clinical Gerontologist. 2016;39(1):25-47. | Non-LMIC |
| 299 | Winter L, Gitlin LN. Evaluation of a telephone-based support group intervention for female caregivers of community-dwelling individuals with dementia. Am J Alzheimers Dis Other Demen. 2006;21(6):391-7. | Non-LMIC |
| 300 | Winter L, Gitlin LN. Evaluation of a Telephone-Based Support Group Intervention for Female Caregivers of Community-Dwelling Individuals With Dementia. American Journal of Alzheimer's Disease & Other Dementias®. 2007;21(6):391-7. | Non-LMIC |
| 301 | Zanetti O, Metitieri T, Bianchetti A, Trabucchi M. Effectiveness of an educational program for demented person's relatives. Archives of Gerontology and Geriatrics. 1998;26:531-8. | Non-LMIC |
| 302 | Zimmerman S, Sloane PD, Ward K, Beeber A, Reed D, Lathren C, et al. Helping dementia caregivers manage medical problems: Benefits of an educational resource. American Journal of Alzheimer's Disease & Other Dementias®. 2018;33(3):176-83. | Non-LMIC |
| 303 | Davis JD, Tremont G, Bishop DS, Fortinsky RH. A telephone-delivered psychosocial intervention improves dementia caregiver adjustment following nursing home placement. International Journal of Geriatric Psychiatry. 2011;26(4):380-7. | Non-LMIC |
| 304 | Ducharme F, Lévesque L, Giroux F, Lachance L. Follow-up of an intervention program for caregivers of a relative with dementia living in a long-term care setting: Are there any persistent and delayed effects? Aging & Mental Health. 2005;9(5):461-9. | Non-LMIC |
| 305 | Gaugler JE, Reese M, Sauld J. A Pilot Evaluation of Psychosocial Support for Family Caregivers of Relatives with Dementia in Long-Term Care: The Residential Care Transition Module. Res Gerontol Nurs. 2015;8(4):161-72. | Non-LMIC |
| 306 | McCallion P, Toseland RW, Freeman K. An Evaluation of a Family Visit Education Program. Journal of the American Geriatrics Society. 1999;47(2):203-14. | Non-LMIC |
| 307 | Paun O, Farran CJ, Fogg L, Loukissa D, Thomas PE, Hoyem R. A chronic grief intervention for dementia family caregivers in long-term care. West J Nurs Res. 2015;37(1):6-27. | Non-LMIC |
| 308 | Gaugler JE, Reese M, Sauld J. A Pilot Evaluation of Psychosocial Support for Family Caregivers of Relatives with Dementia in Long-Term Care: The Residential Care Transition Module. Res Gerontol Nurs. 2015;8(4):161-72. | Duplicate (not picked up due to way exported to EndNote) |
| 309 | Fowler C, Haney T, Rutledge CM. An interprofessional virtual healthcare neighborhood for caregivers of elderly with dementia. J Nurse Pract. 2014;10(10):829-34. | Duplicate (not picked up due to way exported to EndNote) |
| 310 | Abrahams R, Liu KPY, Bissett M, Fahey P, Cheung KSL, Bye R, et al. Effectiveness of interventions for co-residing family caregivers of people with dementia: Systematic review and meta-analysis. Australian Occupational Therapy Journal. 2018;65(3):208-24. | Review of existing literature, relevant for further screening |
| 311 | Christie HL, Bartels SL, Boots LMM, Tange HJ, Verhey FRJ, de Vugt ME. A systematic review on the implementation of eHealth interventions for informal caregivers of people with dementia. Internet Interventions. 2018;13:51-9. | Review of existing literature, relevant for further screening |
| 312 | Collins RN, Kishita N. The Effectiveness of Mindfulness- and Acceptance-Based Interventions for Informal Caregivers of People With Dementia: A Meta-Analysis. Gerontologist. 2019;59(4):e363-e79. | Review of existing literature, relevant for further screening |
| 313 | Dam AEH, de Vugt ME, Klinkenberg IPM, Verhey FRJ, van Boxtel MPJ. A systematic review of social support interventions for caregivers of people with dementia: Are they doing what they promise? Maturitas. 2016;85:117-30. | Review of existing literature, relevant for further screening |
| 314 | Deeken F, Rezo A, Hinz M, Discher R, Rapp MA. Evaluation of Technology-Based Interventions for Informal Caregivers of Patients With Dementia—A Meta-Analysis of Randomized Controlled Trials. The American Journal of Geriatric Psychiatry. 2019;27(4):426-45. | Review of existing literature, relevant for further screening |
| 315 | Egan KJ, Pinto-Bruno ÁC, Bighelli I, Berg-Weger M, van Straten A, Albanese E, et al. Online Training and Support Programs Designed to Improve Mental Health and Reduce Burden Among Caregivers of People With Dementia: A Systematic Review. Journal of the American Medical Directors Association. 2018;19(3):200-6.e1. | Review of existing literature, relevant for further screening |
| 316 | Gallagher-Thompson D, Coon DW. Evidence-based psychological treatments for distress in family caregivers of older adults. Psychol Aging. 2007;22(1):37-51. | Review of existing literature, relevant for further screening |
| 317 | Henry Brodaty, D.Sc. , and, Caroline Arasaratnam, B.Psych. Meta-Analysis of Nonpharmacological Interventions for Neuropsychiatric Symptoms of Dementia. American Journal of Psychiatry. 2012;169(9):946-53. | Review of existing literature, relevant for further screening |
| 318 | Hopkinson MD, Reavell J, Lane DA, Mallikarjun P. Cognitive Behavioral Therapy for Depression, Anxiety, and Stress in Caregivers of Dementia Patients: A Systematic Review and Meta-Analysis. The Gerontologist. 2018;59(4):e343-e62. | Review of existing literature, relevant for further screening |
| 319 | Kaddour L, Kishita N, Schaller A. A meta-analysis of low-intensity cognitive behavioral therapy-based interventions for dementia caregivers. International Psychogeriatrics. 2019;31(7):961-76. | Review of existing literature, relevant for further screening |
| 320 | Kishita N, Hammond L, Dietrich CM, Mioshi E. Which interventions work for dementia family carers?: an updated systematic review of randomized controlled trials of carer interventions. International Psychogeriatrics. 2018;30(11):1679-96. | Review of existing literature, relevant for further screening |
| 321 | Kor PPK, Chien WT, Liu JYW, Lai CKY. Mindfulness-Based Intervention for Stress Reduction of Family Caregivers of People with Dementia: A Systematic Review and Meta-Analysis. Mindfulness. 2018;9(1):7-22. | Review of existing literature, relevant for further screening |
| 322 | Lamotte G, Shah RC, Lazarov O, Corcos DM. Exercise Training for Persons with Alzheimer's Disease and Caregivers: A Review of Dyadic Exercise Interventions. J Mot Behav. 2017;49(4):365-77. | Review of existing literature, relevant for further screening |
| 323 | Laver K, Milte R, Dyer S, Crotty M. A Systematic Review and Meta-Analysis Comparing Carer Focused and Dyadic Multicomponent Interventions for Carers of People With Dementia. Journal of aging and health. 2017;29(8):1308-49. | Review of existing literature, relevant for further screening |
| 324 | Liu Z, Chen Q-L, Sun Y-Y. Mindfulness training for psychological stress in family caregivers of persons with dementia: a systematic review and meta-analysis of randomized controlled trials. Clin Interv Aging. 2017;12:1521-9. | Review of existing literature, relevant for further screening |
| 325 | Morris L, Horne M, McEvoy P, Williamson T. Communication training interventions for family and professional carers of people living with dementia: a systematic review of effectiveness, acceptability and conceptual basis. Aging & Mental Health. 2018;22(7):863-80. | Review of existing literature, relevant for further screening |
| 326 | Nguyen H, Terry D, Phan H, Vickers J, McInerney F. Communication training and its effects on carer and care-receiver outcomes in dementia settings: A systematic review. J Clin Nurs. 2019;28(7-8):1050-69. | Review of existing literature, relevant for further screening |
| 327 | Parker D, Mills S, Abbey J. Effectiveness of interventions that assist caregivers to support people with dementia living in the community: a systematic review. Int J Evid Based Healthc. 2008;6(2):137-72. | Review of existing literature, relevant for further screening |
| 328 | Wu B, Petrovsky DV, Wang J, Xu H, Zhu Z, McConnell ES, et al. Dementia caregiver interventions in Chinese people: A systematic review. J Adv Nurs. 2019;75(3):528-42. | Review of existing literature, relevant for further screening |
| 329 | Arruda EH, Paun O. Dementia Caregiver Grief and Bereavement: An Integrative Review. West J Nurs Res. 2017;39(6):825-51. | Review of existing literature, relevant for further screening |
| 330 | Chacko E, Ling B, Avny N, Barak Y, Cullum S, Sundram F, et al. Mindfulness-Based Cognitive Therapy for Stress Reduction in Family Carers of People Living with Dementia: A Systematic Review. International Journal of Environmental Research and Public Health. 2022;19(1):614. | Review of existing literature, relevant for further screening |
| 331 | Etxeberria I, Salaberria K, Gorostiaga A. Online support for family caregivers of people with dementia: a systematic review and meta-analysis of RCTs and quasi-experimental studies. Aging & Mental Health. 2021;25(7):1165-80. | Review of existing literature, relevant for further screening |
| 332 | Frias CE, Garcia-Pascual M, Montoro M, Ribas N, Risco E, Zabalegui A. Effectiveness of a psychoeducational intervention for caregivers of People With Dementia with regard to burden, anxiety and depression: A systematic review. J Adv Nurs. 2020;76(3):787-802. | Review of existing literature, relevant for further screening |
| 333 | Hopwood J, Walker N, McDonagh L, Rait G, Walters K, Iliffe S, et al. Internet-Based Interventions Aimed at Supporting Family Caregivers of People With Dementia: Systematic Review. J Med Internet Res. 2018;20(6):e216. | Review of existing literature, relevant for further screening |
| 334 | Jackson D, Roberts G, Wu ML, Ford R, Doyle C. A systematic review of the effect of telephone, internet or combined support for carers of people living with Alzheimer’s, vascular or mixed dementia in the community. Archives of Gerontology and Geriatrics. 2016;66:218-36. | Review of existing literature, relevant for further screening |
| 335 | Liu Z, Sun YY, Zhong BL. Mindfulness‐based stress reduction for family carers of people with dementia. Cochrane Database of Systematic Reviews. 2018(8). | Review of existing literature, relevant for further screening |
| 336 | Lucero RJ, Fehlberg EA, Patel AGM, Bjarnardottir RI, Williams R, Lee K, et al. The effects of information and communication technologies on informal caregivers of persons living with dementia: A systematic review. Alzheimer's and Dementia: Translational Research and Clinical Interventions. 2019;5:1-12. | Review of existing literature, relevant for further screening |
| 337 | Parra-Vidales E, Soto-Pérez F, Victoria Perea-Bartolomé M, Franco-Martín MA, Muñoz-Sánchez JL. Online interventions for caregivers of people with dementia: A systematic review. Actas Esp Psiquiatr. 2017;45(3):116-26. | Review of existing literature, relevant for further screening |
| 338 | Powell J, Chiu T, Eysenbach G. A systematic review of networked technologies supporting carers of people with dementia. J Telemed Telecare. 2008;14(3):154-6. | Review of existing literature, relevant for further screening |
| 339 | Rueda Daz LJ, Monteiro da Cruz DL. The efficacy of telephone use to assist and improve the wellbeing of family caregivers of persons with chronic diseases: a systematic review. JBI Evidence Synthesis. 2014;12(12). | Review of existing literature, relevant for further screening |
| 340 | Ruggiano N, Brown Ellen L, Li J, Scaccianoce M. Rural Dementia Caregivers and Technology: What Is the Evidence? Res Gerontol Nurs. 2018;11(4):216-24. | Review of existing literature, relevant for further screening |
| 341 | Shim M, Tilley JL, Im S, Price K, Gonzalez A. A Systematic Review of Mindfulness-Based Interventions for Patients with Mild Cognitive Impairment or Dementia and Caregivers. J Geriatr Psychiatry Neurol. 2021;34(6):528-54. | Review of existing literature, relevant for further screening |
| 342 | Sörensen S, Pinquart M, Duberstein P. How effective are interventions with caregivers? An updated meta-analysis. Gerontologist. 2002;42(3):356-72. | Review of existing literature, relevant for further screening |
| 343 | Tang WK, Chan CYJ. Effects of psychosocial interventions on self-efficacy of dementia caregivers: a literature review. International Journal of Geriatric Psychiatry. 2016;31(5):475-93. | Review of existing literature, relevant for further screening |
| 344 | Teahan Á, Lafferty A, McAuliffe E, Phelan A, O’Sullivan L, O’Shea D, et al. Psychosocial Interventions for Family Carers of People With Dementia: A Systematic Review and Meta-Analysis. Journal of Aging and Health. 2020;32(9):1198-213. | Review of existing literature, relevant for further screening |
| 345 | Walter E, Pinquart M. How Effective Are Dementia Caregiver Interventions? An Updated Comprehensive Meta-Analysis. Gerontologist. 2020;60(8):609-19. | Review of existing literature, relevant for further screening |
| 346 | Wennberg, Alexandra, Dye, Cheryl, Streetman-Loy, Blaiz, Pham, Hiep. Alzheimer's Patient Familial Caregivers: A Review of Burden and Interventions. Health & Social Work. 2015;40(4):e162-e9. | Review of existing literature, relevant for further screening |
| 347 | Campos CRF, Carvalho TRd, Barham EJ, Andrade LFd, Giannini AS. Entender e envolver: avaliando dois objetivos de um programa para cuidadores de idosos com Alzheimer. Psico. 2019;50(e29444). | English/Vietnamese FT Unavailable |
| 348 | Ferreira CR, Barham EJ. Uma intervenção para reduzir a sobrecarga em cuidadores que assistem idosos com doença de Alzheimer  An intervention to reduce burden among caregivers who assist elderly people with Alzheimer’s disease. Revista Kairós Gerontologia. 2016;19(4):111-30. | English/Vietnamese FT Unavailable |
| 349 | Huang B, Wei H, He J. The effect of cognitive behavioral intervention on coping style and emotional state of primary caregivers of Alzheimer′ s disease. Anhui Medical and Pharmaceutical Journal. 2019;3:580-3. | English/Vietnamese FT Unavailable |
| 350 | Huang YN. Impact of comprehensive intervention on caregivers of patients with Alzheimer’s disease. Health Guide Med Res. 2015;7:66-7. | English/Vietnamese FT Unavailable |
| 351 | Liu QSS, S.M.; Wang, Z.W.; Fu, Y.; Yue, P.; Zhang, H.; Liu, Y. 护理干预对减轻居家痴呆照顾者抑郁焦虑情绪的效果评价  Effects of a one-year nursing intervention on depression and anxiety in family caregivers of homebound patients with dementia. Chinese Journal of Behavioral Medicine and Brain Science. 2007;16. | English/Vietnamese FT Unavailable |
| 352 | Luo LCW, J.A. . Effect evaluation of nursing intervention on alleviating depression and anxiety among caregivers with Alzheimer’s disease at home. Today Nurse. 2012;10:22-4. | English/Vietnamese FT Unavailable |
| 353 | Pan YXZ, H.Y. . Study of the Effect of Psychosocial Intervention on Depressive Psychology and Care Burden of Family Caregivers of Dementia Patients. . Shanghai Med Pharm J 2020;41:13–5, 49. | English/Vietnamese FT Unavailable |
| 354 | Zhu B, Lin Z, He X, Shao Z. Influence of collaborative care model on the burden of family caregivers of senile dementia patients in the community. Mod Med J. 2016;44:1780-4. | English/Vietnamese FT Unavailable |
| 355 | 1. 姚爱华, Yao AH. 认知行为干预对老年痴呆患者照护者的影响  Effect of cognitive-behavior intervention on caregivers of alzheimer's disease patients. 中华现代护理杂志  Chin J Mod Nurs. 2011;17(4):373-5. | English/Vietnamese FT Unavailable |
| 356 | 王姝亚, 金丽芬, 白志仙, 刘凯, 高娟, 吴婷婷. 系统心理干预在缓解阿尔茨海默病患者照顾者抑郁症状中的实践  Effect of the comprehensive intervention on depression of caregivers of patients with Alzheimer's Disease. 中国护理管理. 2018;18(2):268-72. | English/Vietnamese FT Unavailable |
| 357 | 费静霞, 赵徐萍, 沈丽珍, Fei JXZ, X.P.; Shen, L.Z. . 综合护理干预对改善老年痴呆患者亲属情绪障碍及应对方式的影响  Investigation on nursing intervention improving coping style and emotional disorder of relatives of the senile dementia in hospital. 中华现代护理杂志  Chin J Mod Nurs. 2009;15(23):2249-51. | English/Vietnamese FT Unavailable |
| 358 | Fialho PPA, Köenig AM, Santos MDLD, Barbosa MT, Caramelli P. Positive effects of a cognitive-behavioral intervention program for family caregivers of demented elderly. Arq Neuro-Psiquiatr. 2012;70(10):786-92. | Non-RCT |
| 359 | Foss MP, Lange C, Silva Filho JH, Brunini F, Carvalho do Vale FA. Support groups for caregivers of patients with Dementia. A comparative study. Dementia & Neuropsychologia. 2007;1(2):196-202. | Non-RCT |
| 360 | Norouzi M, Golzari M, Sohrabi F. Effectiveness of Mindfulness Based Cognitive Therapy on the Quality of Life, Depression and Burden of Demented Women Caregivers. Zahedan J Res Med Sci. 2014;16(9). | Non-RCT |
| 361 | Mollaei P, Momtaz YA, Taheri-Tanjani P. The Effect of Cyberspace-Based Education on the Well-Being of Caregivers of Elderly People with Dementia. Curr Aging Sci. 2021;14(2):105-11. | Non-RCT |
| 362 | Pagán-Ortiz ME, Cortés DE, Rudloff N, Weitzman P, Levkoff S. Use of an Online Community to Provide Support to Caregivers of People With Dementia. Journal of Gerontological Social Work. 2014;57(6-7):694-709. | Non-RCT |
| 363 | Boyd K, Nugent C, Donnelly M, Bond R, Sterritt R, Hartin P, editors. An investigation into the usability of the STAR training and re-skilling website for carers of persons with dementia. 2014 36th Annual International Conference of the IEEE Engineering in Medicine and Biology Society; 2014: IEEE. | Conference abstract (searched for and unable to find FT elsewhere) |
| 364 | Marx K, Werner N, Kales H, Turnwald M, Stanislawski B, Gitlin L, editors. USABILITY TESTING OF A WEB-BASED APPLICATION FOR CAREGIVERS OF PEOPLE WITH DEMENTIA: THE WECARE ADVISOR TOOL. Gerontologist; 2015: OXFORD UNIV PRESS INC JOURNALS DEPT, 2001 EVANS RD, CARY, NC 27513 USA. | Conference abstract (searched for and unable to find FT elsewhere) |
| 365 | Kales HC, Gitlin LN, Lyketsos C. “WECARE ADVISOR”: A CLINICAL TRIAL OF A CAREGIVER FOCUSED, IPAD ADMINISTERED ALGORITHM TO MANAGE BEHAVIORAL SYMPTOMS. Alzheimer's dement. 2016;12(7, Supplement):P217. | Conference abstract (searched for and found FT elsewhere) |
| 366 | Marx KA, Gitlin LN, Lyketsos C, Kales HC, Stanislawski B. O3‐06‐05: Testing a Web‐Based Application to Help Informal Caregivers Manage Behaviors in Persons with Dementia: WecareadvisorTM. 2016. | Conference abstract (searched for and found FT elsewhere) |
| 367 | Lamech N, Lakshminarayanan M, Vaitheswaran S, John S, Rangaswamy T. Support groups for family caregivers of persons with dementia in India. Dementia. 2020;20(3):1172-81. | Duplicate |
| 368 | Perkins L, Fisher E, Felstead C, Rooney C, Wong GHY, Dai R, et al. Delivering Cognitive Stimulation Therapy (CST) Virtually: Developing and Field-Testing a New Framework. Clin Interv Aging. 2022;17:97-116. | Development/acceptability process, no results from intervention |
| 369 | Rong X, Wu F, Tang D, Zhao Y, Guo Q, He F, et al. Development of a self-management support program for caregivers of relatives with dementia in Shanghai. Geriatr Nurs. 2020;41(2):98-104. | Non-RCT |
| 370 | Kales HC, Gitlin LN, Stanislawski B, Myra Kim H, Marx K, Turnwald M, et al. Effect of the WeCareAdvisor™ on family caregiver outcomes in dementia: a pilot randomized controlled trial. BMC Geriatrics. 2018;18(1):113. | Non-LMIC |
| 371 | Gauthier S WC, Servaes S, Morais JA, Rosa-Neto P. World Alzheimer Report 2022: Life after diagnosis: Navigating treatment, care and support. London, England: Alzheimer's Disease International; 2022. | Review of existing literature, relevant for further screening |
| 372 | Acton GJ, Kang J. Interventions to reduce the burden of caregiving for an adult with dementia: a meta-analysis. Research in Nursing and Health. 2001;24(5):349-60. | Review of existing literature, relevant for further screening |
| 373 | Boots LM, de Vugt ME, van Knippenberg RJ, Kempen GI, Verhey FR. A systematic review of Internet-based supportive interventions for caregivers of patients with dementia. International Journal of Geriatric Psychiatry. 2013;29(4):331-44. | Review of existing literature, relevant for further screening |
| 374 | Brodaty H, Green A, Koschera A. Meta-analysis of psychosocial interventions for caregivers of people with dementia. Journal of the American Geriatrics Society. 2003;51(5):657-64. | Review of existing literature, relevant for further screening |
| 375 | Chien LY, Chu H, Guo JL, Liao YM, Chang LI, Chen CH, et al. Caregiver support groups in patients with dementia: a meta-analysis. International Journal of Geriatric Psychiatry. 2011;26(10):1089-98. | Review of existing literature, relevant for further screening |
| 376 | Cooper C, Balamurali TB, Selwood A, Livingston G. A systematic review of intervention studies about anxiety in caregivers of people with dementia. International Journal of Geriatric Psychiatry. 2007;22(3):181-8. | Review of existing literature, relevant for further screening |
| 377 | Corbett A, Stevens J, Aarsland D, Day S, Moniz-Cook E, Woods R, et al. Systematic review of services providing information and/or advice to people with dementia and/or their caregivers. International Journal of Geriatric Psychiatry. 2012;27(6):628-36. | Review of existing literature, relevant for further screening |
| 378 | de Oliveira Lopes L, Cachioni M. [Psychoeducational intervention for caregivers of elderly with dementia: a systematic review]. Jornal Brasileiro de Psiquiatria. 2012;61(4):252-61. | Review of existing literature, relevant for further screening |
| 379 | Elvish R, Lever SJ, Johnstone J, Cawley R, Keady J. Psychological interventions for carers of people with dementia: a systematic review of quantitative and qualitative evidence. British Association for Counselling & Psychotherapy. 2012:01-32. | Review of existing literature, relevant for further screening |
| 380 | Godwin KM, Mills WL, Anderson JA, Kunik ME. Technology-driven interventions for caregivers of persons with dementia: a systematic review. American Journal of Alzheimer's Disease and Other Dementias. 2013;28(3):216-22. | Review of existing literature, relevant for further screening |
| 381 | Hurley RV, Patterson TG, Cooley SJ. Meditation-based interventions for family caregivers of people with dementia: a review of the empirical literature. Aging and Mental Health. 2014;18(3):281-8. | Review of existing literature, relevant for further screening |
| 382 | Li R, Cooper C, Austin A, Livingston G. Do changes in coping style explain the effectiveness of interventions for psychological morbidity in family carers of people with dementia? A systematic review and meta-analysis. International Psychogeriatrics. 2013;25(2):204-14. | Review of existing literature, relevant for further screening |
| 383 | Lins S, Hayder-Beichel D, Rücker G, Motschall E, Antes G, Meyer G, et al. Efficacy and experiences of telephone counselling for informal carers of people with dementia. Cochrane Database of Systematic Reviews: Reviews. 2014;Issue 9. | Review of existing literature, relevant for further screening |
| 384 | Marim CM, Silva V, Taminato M, Barbosa DA. Effectiveness of educational programs on reducing the burden of caregivers of elderly individuals with dementia: a systematic review. Revista Latino-Americana de Enfermagem. 2013;21(Special Issue):267-75. | Review of existing literature, relevant for further screening |
| 385 | McKechnie V, Barker C, Stott J. Effectiveness of computer-mediated interventions for informal carers of people with dementia – a systematic review. International Psychogeriatrics. 2014;26(10):1619-37. | Review of existing literature, relevant for further screening |
| 386 | Nelis S, Quinn C, Clare L. Information and support interventions for informal caregivers of people with dementia. Cochrane Database of Systematic Reviews: Reviews. 2007;Issue 2. | Review of existing literature, relevant for further screening |
| 387 | Peacock SC, Forbes DA. Interventions for caregivers of persons with dementia: a systematic review. Canadian Journal of Nursing Research. 2003;35(4):88-107. | Review of existing literature, relevant for further screening |
| 388 | Pinquart M, Sorensen S. Helping caregivers of persons with dementia: which interventions work and how large are their effects? International Psychogeriatrics. 2006;18(4):577-95. | Review of existing literature, relevant for further screening |
| 389 | Pusey H, Richards D. A systematic review of the effectiveness of psychosocial interventions for carers of people with dementia. Aging and Mental Health. 2001;5(2):107-19. | Review of existing literature, relevant for further screening |
| 390 | Schoenmakers B, Buntinx F, DeLepeleire J. Supporting the dementia family caregiver: the effect of home care intervention on general well-being. Aging and Mental Health. 2010;14(1):44-56. | Review of existing literature, relevant for further screening |
| 391 | Selwood A, Johnston K, Katona C, Lyketsos C, Livingston G. Systematic review of the effect of psychological interventions on family caregivers of people with dementia. Journal of Affective Disorders. 2007;101(1-3):75-89. | Review of existing literature, relevant for further screening |
| 392 | Smits CH, de Lange J, Droes RM, Meiland F, Vernooij-Dassen M, Pot AM. Effects of combined intervention programmes for people with dementia living at home and their caregivers: a systematic review. International Journal of Geriatric Psychiatry. 2007;22(12):1181-93. | Review of existing literature, relevant for further screening |
| 393 | Thompson CA, Spilsbury K, Hall J, Birks Y, Barnes C, Adamson J. Systematic review of information and support interventions for caregivers of people with dementia. BMC Geriatrics. 2007;7:18. | Review of existing literature, relevant for further screening |
| 394 | Van't Leven N, Prick AE, Groenewoud JG, Roelofs PD, de Lange J, Pot AM. Dyadic interventions for community-dwelling people with dementia and their family caregivers: a systematic review. International Psychogeriatrics. 2013;25(10):1581-603. | Review of existing literature, relevant for further screening |
| 395 | Zientz J, Rackley A, Chapman SB, Hopper T, Mahendra N, Kim ES, et al. Evidence-based practice recommendations for dementia: educating caregivers on Alzheimer's disease and training communication strategies. Journal of Medical Speech-Language Pathology. 2007;15(1):liii-lxiv. | Review of existing literature, relevant for further screening |
| 396 | M. JJaP. World Alzheimer Report 2009. London, UK: Alzheimer's Disease International; 2009. | Review of existing literature, relevant for further scan |
| 397 | Amador S, Rapaport P, Lang I, Sommerlad A, Mukadam N, Stringer A, et al. Implementation of START (STrAtegies for RelaTives) for dementia carers in the third sector: Widening access to evidence-based interventions. PLoS ONE. 2021;16(6):e0250410-e. | Non-LMIC |
| 398 | Blom MM, Zarit SH, Groot Zwaaftink RBM, Cuijpers P, Pot AM. Effectiveness of an Internet intervention for family caregivers of people with dementia: results of a randomized controlled trial. PLoS ONE. 2015;10(2):e0116622-e. | Non-LMIC |
| 399 | Joling KJ, van Marwijk HWJ, Smit HFE, van der Horst HE, Scheltens P, van de Ven PM, et al. Does a family meetings intervention prevent depression and anxiety in family caregivers of dementia patients? A randomized trial. PLoS ONE. 2012;7(1):e30936-e. | Non-LMIC |
| 400 | Findings from School of Medicine Update Understanding of Dementia (Implementing the Dementia Carers Support Initiative of the National Institute for Health and Care Excellence in a sub-Saharan African Setting). Mental Health Weekly Digest. 2015:59. | Duplicate – appeared elsewhere in the study (not screened prior to this point due to the way it was listed in JOLIS |
| 401 | Researchers from University of California Davis School of Medicine Report New Studies and Findings in the Area of Alzheimer Disease (Interventions to support family caregivers of people living with dementia in high, middle and low-income ...). Mental Health Weekly Digest. 2019:616. | Duplicate – appeared elsewhere in the study (not screened prior to this point due to the way it was listed in JOLIS |
| 402 | Bourne P, Camic P, Crutch S. Psychosocial outcomes of dyadic arts interventions for people with a dementia and their informal caregivers: A systematic review. Health Soc Care Community. 2021;29:1632–49. | Review of existing literature, relevant for further screening |
| 403 | Adams T. Developing partnerships between people with dementia, their informal family carers and community psychiatric nurses [U - Thesis ; (Thesis (Ph.D.))]: Surrey Univ., Guildford (United Kingdom); 2001. | Unable to obtain full text |
| 404 | Archibald C. Activities and people with dementia Involving family carers. 1999. | Unable to obtain full text |
| 405 | Diebolt J. Assessment of the benefits of an information program for family caregivers of patients affected by dementia/Evaluation des bénéfices d'un programme de formation des aidants familiaux de patients atteints de la maladie d'Alzheimer et maladies apparentées [U - Thesis ; (Thesis)]: Université de Strasbourg, Faculté de médecine, 2009; 2014. | Unable to obtain full text |
| 406 | DUFOUR N. Dedicated interventions for dementia caregivers by general practitionners : a systematic review  Les interventions dédiées aux aidants familiaux de patients déments par le médecin généraliste (Revue de la littérature) [U - Thesis ; (Thesis)]: Université de Rouen; 2013. | Review of existing literature, relevant for further screening |
| 407 | Alcaraz Agüero M, Fong Estrada A, Laborí Ruiz JR, Alayo Blanco M, García Álvarez R. Capacitación a personas cuidadoras de pacientes con demencia. Medisan. 2009;13(2). | English/Vietnamese full text unavailable |
| 408 | Brum AKR, Camacho ACLF, Valente GSC, Sá SPC, Lindolpho MdC, Louredo DdS. Programa para cuidadores de idosos com demência: relato de experiência. Rev bras enferm. 2013;66(4):619-24. | English/Vietnamese full text unavailable |
| 409 | Cerquera Córdoba Am, Pabón Poches DK. Intervención en cuidadores informales de pacientes con demencia en Colombia: una revisión. Psychol av discip. 2014;8(2):73-81. | English/Vietnamese full text unavailable |
| 410 | Mondéjar Barrios MD, Junco Martínez GJ, Hernández Martínez N. Intervención educativa a cuidadores de pacientes con demencia de Alzheimer del área norte de Morón. Mediciego. 2011;17(Supl. 1). | English/Vietnamese full text unavailable |
| 411 | Reis Ed, Novelli MMPC, Guerra RLF. Intervenções realizadas com grupos de cuidadores de idosos com síndrome demencial: revisão sistemática. Cad Bras Ter Ocup. 2018;26(3):646-57. | Review of existing literature, relevant for further screening |
| 412 | Youngran TAK, Junghee S, Haeyoung WOO, Jiyeon AN. Realist Review: Understanding Effectiveness of Intervention Programs for Dementia Caregivers. Asian Nursing Research. 2019:11-9. | Review of existing literature, relevant for further screening |
| 413 | Jiyoung Y, Rayoung YOO, Seonghoon BAE, Yeonwook K, Geon-Ha KIM, Hae-Ri NA, et al. Recent Updates of Therapeutic Intervention Programs for Caregivers of Patient with Dementia: Proposal of Hospital-Based Individual Therapy. Dementia and Neurocognitive Disorders. 2016:29-36. | Non-LMIC |
| 414 | Fugu T. A randomized controlled trial of the positive diary intervention for family caregivers of people with dementia - A randomized controlled trial of the positive diary intervention for family caregivers of people with dementia. 2020. | Non-LMIC |
| 415 | Kajiyama B. Caring Light Project for Caregivers of Individuals With Alzheimer's Disease. 2020. | Non-LMIC |
| 416 | Miyaoka H. A forward search exploratory study on the validity and effectiveness of group therapy program for family caregivers of elderly with dementia. 2019. | Non-LMIC |
| 417 | Shyu YL. Establishment and Effectiveness of a Community-based Long-term Care Model for Elderly Persons With Dementia. 2016. | Non-LMIC |
| 418 | Martins A. The effect of the World Health Organization's iSupport in Brazil (Translation, Cultural Adaptation and the Effects of the World Health Organization's iSupport for the Brazilian Context). 2023. | Protocol/registered trial with no results published yet |
| 419 | Sanprakhon P. Effectiveness of an Integrative Stress Reduction Program for Family Caregivers of People with Advanced Dementia: A Randomized Control Trial. 2023. | Protocol/registered trial with no results published yet |
| 420 | Hinton L. Single Arm Study of Modified REACH-VA Alzheimer's Caregiver Intervention in Vietnam. 2017. | Protocol/registered trial with results found elsewhere in review |
| 421 | F GH, S P, J M, Z G. [Effect of family education program on depression, anxiety and stress of family caregivers of elderly individuals with dementia]. 2014;3(1):12-20. | Unable to obtain full text in English/Vietnamese |
| 422 | Afaf Mahmoud F, Hanaa H AH, Sorayia R AF. Dementia: nursing intervention for family care givers. 2005;33(3):150-62. | Unable to obtain full text |
| 423 | Salamizadeh A, Mirzaei T, Ravari A. The impact of spiritual care education on the self-efficacy of the family caregivers of elderly people with Alzheimer’s disease. 2017;5:231-8. | Non-randomised |
| 424 | Phanida K. Dementia Care System in Suratthani Hospital. 2010. | Unable to obtain full text in English/Vietnamese |
| 425 | Heup S, Jae-Myeong K, Steven BK, Seong-Jin CHO, Seonkyung YUN, Hyejin Y, et al. Comparative Study of the Effects of Supportive Educational Programs : for Family Caregivers of the Elderly with Dementia. Journal of Korean Geriatric Psychiatry. 2017:17-28. | Unable to obtain full text in English/Vietnamese |
| 426 | Jieun J, Heeseung C. The Effects of Emotion-focused Individual Intervention for Family Caregivers of People with Early Stages of Dementia. Journal of Korean Academy of Psychiatric and Mental Health Nursing. 2016:101-12. | Unable to obtain full text in English/Vietnamese |
| 427 | Kye YK, Sang-Joon SON. Caregiver management of psychiatric disorders of the elderly in dementia. Journal of the Korean Medical Association. 2010:993-8. | Unable to obtain full text in English/Vietnamese |
| 428 | Ruifen Z, Lizhen S, Junxiang J. Application effect of family intervention model in early Alzheimer's disease patients' family. Chinese Journal of Practical Nursing. 2013(36):28-31. | Unable to obtain full text in English/Vietnamese |
| 429 | Ilsung NAM. Effects of Social Support on Caregiver Burden and Depressive Symptoms in Family Caregivers of Patients with Alzheimer's Disease. Korean Journal of Health Promotion. 2013:156-63. | Non-LMIC |
| 430 | Kwang-Soo YOO, Eun-Sun SO. The Effectiveness of Dementia Education for the Nursing Method of Family Caregivers of the Demented Elderly. Journal of Korean Academy of Community Health Nursing. 2014:97-108. | Non-LMIC |
| 431 | Suk-Hee C, Sung-Ok C, Gye-Soon K, Mi-Ryeong S. Development and Evaluation of Community-based Respite Program for Family Caregivers of Elders with Dementia. Journal of Korean Academy of Fundamental Nursing. 2011:337-47. | Non-LMIC |
| 432 | Nuraisyah Hani Z, Suriani I, Rosliza Abdul M, Lim Poh Y. Educational Intervention for Informal Caregiver of Person with Dementia: A Systematic Review. Malaysian Journal of Medicine and Health Sciences. 2020:325-31. | Review of existing literature, relevant for further screening |
| 433 | Seyeon P, Myonghwa P. Effects of Family Support Programs for Caregivers of People with Dementia - Caregiving Burden, Depression, and Stress: Systematic Review and Meta-analysis. Journal Korean acad. 2015:627-40. | Review of existing literature, relevant for further screening |
| 434 | Dilmini K. “I WISH YOU WOULD JUST DIE!” MANAGEMENT OF DEMENTIA WITH BPSD, AND CAREGIVER STRESS. The Singapore Family Physician. 2018:61-3. | Case-study |
| 435 | Mushtaq Shahnawaz GRK, Fatima Zeenat. The effectiveness of brief cognitive-behavioral therapy on guilt feelings of dementia caregivers. ASIAN JOURNAL OF MULTIDIMENSIONAL RESEARCH. 2018;7(7):100-10. | Non-randomised |
| 436 | Rao G, Sivakumar P, Srivastava S, Sidana R. Cognitive Therapy and Family Intervention for Patients with Dementia and Psychosis. Indian Journal of Psychiatry. 2020;62(8):183-91. | Overview of theory of therapies, not intervention |
| 437 | Won-Mee Jeong S-HP, Kyoung-Young Park, So-Yeon Park, Jong-Chul Youn. Effects of a functional routine enabling program on behavioral and psychological symptoms of dementia in patients with Alzheimer's disease and on caregiver burden. Indian Journal of Science and Technology. 2016;9(43):1-8. | Non-LMIC |
| 438 | Parswani Manish SM, Bharath Srikala. Home-Based behavioural family intervention in dementia : A case illustration. Indian Journal of Gerontology. 2008;22(1):14-26. | Case study (single case presentation) |
| 439 | Acosta Ortega LF. A propósito del artículo Estimulación cognitiva y apoyo familiar hacia el adulto mayor con deterioro cognitivo. Rev inf cient. 2019;98(2):143-5. | Unable to obtain full text in English/Vietnamese |
| 440 | Andrade LM, Sena ELdS, Carvalho PALd, Matos ALPd, Mercês MCd, Oliveira DS. Suporte familiar ao cuidador da pessoa com doença de Alzheimer. Rev Kairós. 2014;17(4):275-95. | Unable to obtain full text in English/Vietnamese |
| 441 | Camacho ACLF. Metodologia assistencial para a pessoa com Doença de Alzheimer e sua rede de suporte: proposição de um modelo de cuidados de enfermagem. 2010. p. 244-. | Unable to obtain full text in English/Vietnamese |
| 442 | Damé CF, Barea BM, Cataldo Neto A, Zimmermann PR, Gauer GJC. Demência: manejo do paciente e impacto na família. Acta méd (Porto Alegre). 2006;27:367-73. | Unable to obtain full text in English/Vietnamese |
| 443 | Figueiredo D, Guerra S, Marques A, Sousa L. Apoio psicoeducativo a cuidadores familiares e formais de pessoas idosas com demência. Rev Kairós. 2012;15(11,n.esp):31-55. | Unable to obtain full text in English/Vietnamese |
| 444 | Lima CFdM. O cuidado domiciliar ao idoso com doença de Alzheimer: representações de familiares cuidadores, membros de uma associação de apoio, na cidade de Salvador-BA. 2002. p. 126-. | Unable to obtain full text in English/Vietnamese |
| 445 | Lopes LdO, Cachioni M. Cuidadores familiares de idosos com doença de Alzheimer em uma intervenção psicoeducacional. Rev bras geriatr gerontol. 2013;16(3):443-60. | Unable to obtain full text in English/Vietnamese |
| 446 | Gonzalez FJ, Gaona C, Quintero M, Chavez CA, Selga J, Maestre GE. Building capacity for dementia care inLatin America and the Caribbean. Dement neuropsychol. 2014;8(4). | Review of existing literature, relevant for further screening |
| 447 | O'Connor CM, Clemson L, Silva TBLd, Piguet O, Hodges JR, Mioshi E. Enhancement of carer skills and patient function in the non-pharmacological management of frontotemporal dementia - FTD: a call for randomised controlled studies. Dement neuropsychol. 2013;7(2). | Review of existing literature, relevant for further screening |
| 448 | Ruíz Alvarez J, Capó Marrero L, Santamarina Rodríguez SJ, Llanes Torres HM. Intervención educativa en pacientes con demencia e impacto en la calidad de vida de cuidadores. Rev cuba med gen integr. 2018;34(2):1-10. | Non-informal caregiver focused |
| 449 | Abreu WCd. From a syndrome to a social condition: providing palliative care to people with advanced dementia. Rev Rene (Online). 2017;18(1):1-2. | Overview of theory, not intervention |
| 450 | Ponce CC, Ordonez TN, Lima-Silva TB, Santos GDd, Viola LdF, Nunes PV, et al. Effects of a psychoeducational intervention in family caregivers of people with Alzheimer's disease. Dement neuropsychol. 2011;5(3):226-37. | Non-controlled trial |
| 451 | Mattos EBT, Francisco IdC, Pereira GC, Novelli MMPC. Grupo virtual de apoio aos cuidadores familiares de idosos com demência no contexto da COVID-19. Cad Bras Ter Ocup. 2021;29:e2882-e. | Non-RCT |
| 452 | IŞIL OaO, N. Burden of Caregivers of Dementia and Elderly Individuals and Approaches. Turkey Clinics Psychiatric Nursing - Special Topics. 2016;2(1):74-80. | Unable to obtain full text in English/Vietnamese |
| 453 | Varma GSO, N.K.; Ateşci, F.Ç.; Karagöz, N.; and Apa, F. Alzheimer hastasına bakım veren yakınlarında psikodramatik grup terapisi/Psychodramatic group therapy on Alzheimer patients’ caregiver relatives. Anadolu Psikiyatr Derg. 2018;19(6):567-76. | Unable to obtain full text in English/Vietnamese |
| 454 | Yorulmaz ED, G. Dementia Caregivers and Cognitive Behavioral Therapy: A Systematic Review. Current Approaches in Psychiatry. 2021;13(2):170-91. | Review of existing literature, relevant for further screening |
| 455 | Tlapalamatl Toscuento E. Un enfoque Neuro-psicológico en la investigación científica arquitectónica. Arq+ [Internet]. 29 de diciembre de 2017 [citado 25 de marzo de 2024];2(4):13-22. Disponible en: <https://www.lamjol.info/index.php/arquitectura/article/view/9140> | Unable to obtain full text in English/Vietnamese |
| 456 | Codina Velásquez HI, Santos Fuentes SF, Fonseca Pineda EP, Prudot Bardales ZS, Reyes Cabrera I, Mejía Guzmán M. LXIV CONGRESO MÉDICO NACIONAL. Rev Méd Hondur [Internet]. 29 de junio de 2022 [citado 25 de marzo de 2024];90(Supl.1):S1-S93. Disponible en: <https://www.lamjol.info/index.php/RMH/article/view/14511> | Unable to obtain full text in English/Vietnamese |
| 457 | Jahani L, Abolhassani S, Babaee S, Omranifard V. Effects of a compassion-based program on the grief experienced by caregivers of people suffering from dementia: a randomized controlled clinical trial. BMC Nurs. 2022;21(1):198. | Did not use included psychosocial outcome measure |
| 458 | Than, T. N. H., Nguyen, T. T. T., Nguyen, T. C., Vu, L. T. D., Vo, P. T., Truong, K. T., … Nguyen, T. A. (2023). Smartphone app‑based intervention for reducing stress, depression, and anxiety in caregivers of people with dementia in Vietnam: Study protocol for a pilot randomized controlled trial. Digital Health, 9, Article 205520762311637. https://doi.org/10.1177/20552076231163786 | Protocol/registered trial with no results published |
| 459 | Baldassar, L., Doyle, C., Brijnath, B., Goh, A., Cartwright, J., du Toit, S., & Vassilev, I. (2023). Befriending with GENIE: Social intervention to address loneliness and social support for people with dementia and caregivers from Culturally and Linguistically Diverse (CaLD) communities. Edith Cowan University & National Ageing Research Institute. Retrieved from https://www.ecu.edu.au/schools/arts-and-humanities/research-and-creative-activity/migration-diversity-and-care/social-ageing-sage-futures-lab/our-projects/befriending-with-genie | Non-LMIC |
| 460 | Amella Krug, E. J., Qanungo, S., Martin, K. L., Mueller, M., Madisetti, M., & Kelechi, T. J. (2020). A cluster randomized controlled trial to assess the efficacy of a telehealth-based train-the-trainer mealtime intervention delivered by respite care center volunteers to caregivers of persons with dementia to improve nutritional outcomes and quality of life. BMC Nutrition, 6, 24. https://doi.org/10.1186/s40795-020-00350-x | Non-LMIC |
| 461 | Bhardwaj, P., Joshi, N. K., Gupta, M. K., Sharma, S. K., Nebhinani, N., Sinha, A., Tyrrell, M., Konradsen, H., Craftman, Å. G., & Kabir, Z. N. (2025). mHealth‑based intervention by community workers to support family caregivers of persons with dementia living at home: Study protocol for a cluster randomised controlled trial. BMJ Open, 15(1), e087896. <https://doi.org/10.1136/bmjopen-2024-087896> | Protocol/registered trial with no results published |
| 462 | Chen, F., Hu, Z., Li, Q., Li, M., Salcher‑Konrad, M., Comas‑Herrera, A., Knapp, M., Shi, C., & the STRiDE Consortium. (2025). Effectiveness of interventions to support carers of people with dementia in low‑ and middle‑income countries: A systematic review and meta‑analysis. International Journal of Geriatric Psychiatry, 40(3), e70054. https://doi.org/10.1002/gps.70054 | Review of literature relevant for scanning |
| 463 | Gratão, A. C. M., Cardoso, A. M., Ottaviani, A. C., Campos, C. R. F., Oliveira, D. C., Monteiro, D. Q., … Pavarini, S. C. I. (2025, May 29). Effects of iSupport for reducing burden and improving the mental health of informal caregivers of people living with dementia in Brazil: Results of a randomized controlled trial. | Protocol/registered trial with no results published |
| 464 | Evans, I., Patel, R., Stoner, C. R., Melville, M., & Spector, A. (2024). A systematic review of educational interventions for informal caregivers of people living with dementia in low and middle-income countries. Behavioral Sciences, 14(3), 177. https://doi.org/10.3390/bs14030177 | Review of literature relevant for scanning |
| 465 | Huang, H.‑L., Shyu, Y. I. L., Hsu, W.‑C., Liao, Y.‑T., Huang, H. L., & Hsieh, S. H. (2024). Effectiveness of a health education program for people with dementia and their family caregivers: An intervention by nurse practitioners. Archives of Psychiatric Nursing, 50, 147–159. https://doi.org/10.1016/j.apnu.2024.03.018 | Non-LMIC |
| 466 | Kishita, N., Gould, R. L., McCracken, L. M., Khondoker, M., Turner, D. A., Ashford, P.-A., … Farquhar, M. (2024, November). The clinical and cost effectiveness of internet‑delivered self‑help Acceptance and Commitment Therapy for family carers of people with dementia (iACT4CARERS): Study protocol for a randomised controlled trial with ethnically diverse family carers. Contemporary Clinical Trials, 146, 107685. https://doi.org/10.1016/j.cct.2024.107685 | Non-LMIC |
| 467 | Kor, P. P. K., Chou, K. L., Zarit, S. H., Galante, J., Chan, W. C., Tsang, A. P. L., … & Liu, J. Y. W. (2024). Effect of a single-session mindfulness-based intervention for reducing stress in family caregivers of people with dementia: Study protocol for a randomized controlled trial. BMC Psychology, 12(1), Article 582. https://doi.org/10.1186/s40359-024-02027-7 | Protocol/registered trial with no results published |
| 468 | Li, T., Li, J., Leung, A. Y. M., & Schooling, C. M. (2021). Effectiveness of a dyadic technology-enhanced home-based horticultural therapy on psychosocial well-being among people with dementia and their family caregivers: Multimethods pilot study. JMIR Aging, 4(4), e26796. https://doi.org/10.2196/26796 | Non-RCT |
| 469 | Lee, J. A., Kim, J., Rousseau, J., Sabino‑Laughlin, E., Ju, E., Kim, E. A., Rahmani, A., Gibbs, L., & Nyamathi, A. (2024). Community health workers supporting diverse family caregivers of persons with dementia: Preliminary qualitative results from a randomized home-based study. Dementia: The International Journal of Social Research and Practice, 24(2), 249–268. https://doi.org/10.1177/14713012241302367 | Non-LMIC |
| 470 | Lin, H. M., Tung, I. H., Tang, S. H., & Lin, L. J. (2025). The effect of a home-based music care program on caregivers of people living with dementia in Taiwan. Journal of Music Therapy, 62(1), Article thaf006. https://doi.org/10.1093/jmt/thaf006 | Non-LMIC |
| 471 | Liu, Y., Hughes, M. C., Baumbach, A., & Derain, L. (2023). An online intervention to improve the health and well-being of informal caregivers of individuals with Alzheimer’s disease: A pilot study. PEC Innovation, 3, 100229. https://doi.org/10.1016/j.pecinn.2023.100229 | Non-RCT |
| 472 | Oliveira, D., da Mata, F. A. F., & Aubeeluck, A. (2024). Quality of life of family carers of people living with dementia: Review of systematic reviews of observational and intervention studies. British Medical Bulletin, ldad029. https://doi.org/10.1093/bmb/ldad029 | Review of literature relevant for scanning |
| 473 | Osstyn, S. L., Handels, R., Boots, L. M. M., Balvert, S. C. E., Evers, S. M. A. A., & de Vugt, M. E. (2023). The effectiveness and health-economic evaluation of “Partner in Balance,” a blended self-management program for early-stage dementia caregivers: Study protocol for a cluster-randomized controlled trial. Trials, 24, Article 427. https://doi.org/10.1186/s13063-023-07423-9 | Non-LMIC |
| 474 | Perales-Puchalt, J., Ramírez-Mantilla, M., Fracachán-Cabrera, M., Vidoni, E. D., Watts, A., Williams, K., Burns, J. M., & Ellerbeck, E. F. (2024). A text message intervention to support Latino dementia family caregivers (CuidaTEXT): Feasibility study. Clinical Gerontologist, Advance online publication. https://doi.org/10.1080/07317115.2022.2137449 | Non-LMIC |
| 475 | Salcher-Konrad, M., Shi, C., Patel, D., Hu, Z., Vellakkal, S., Wittenberg, R., Comas-Herrera, A., & Knapp, M. (2023). Research evaluating the effectiveness of dementia interventions in low- and middle-income countries: A systematic mapping of 340 randomised controlled trials. International Journal of Geriatric Psychiatry, 38(7), e5965. https://doi.org/10.1002/gps.5965 | Review of literature relevant for scanning |
| 476 | Saragih, I. D., Saragih, I. S., Lee, B. O., Lin, C. J., Tsai, H. T., Chang, Y. S., & Hsu, C. Y. (2023). A meta-analysis of mindfulness-based interventions for improving mental health and burden among caregivers of persons living with dementia. Journal of Advanced Nursing, 79(12), 4034–4048. https://doi.org/10.1111/jan.15888 | Non-RCT |
| 477 | Than, T. N. H., Nguyen, T. T. T., Nguyen, T. C., Vu, L. T. D., Vo, P. T., Truong, K. T., Schofield, P., & Nguyen, T. A. (2023). Smartphone app‑based intervention for reducing stress, depression, and anxiety in caregivers of people with dementia in Vietnam: Study protocol for a pilot randomized controlled trial. Digital Health, 9, Article 20552076231163786. https://doi.org/10.1177/20552076231163786 | Protocol/registered trial with no results published |
| 478 | Wang, K., Miles, T. P., Olsen, J. M., & Poole, L. (2025). Caregiver support program may decrease work-family conflict: An intervention study. Inquiry: The Journal of Health Care Organization, Provision, and Financing, 62, 469580251332766. https://doi.org/10.1177/00469580251332766 | Non-LMIC |
| 479 | Cations, M., Hirakawa, Y., Xu, X., Wang, J., Yin, L., Laver, K., Kuske, S., White, C., Jeon, Y.-H., & Low, L.-F. (2024). The effects of a facilitator-enabled online multicomponent iSupport for dementia programme: A multicentre randomised controlled trial. International Journal of Nursing Studies, 154, 104632. https://doi.org/10.1016/j.ijnurstu.2024.104632 | Non-LMIC |
| 480 | Yuan, S., Zhang, F., Pu, L., Lv, J., Xie, X. F., Lin, L., Qiu, M., Huang, L., Jiang, W., Zhu, J., & Wei, L. (2025). Social support interventions for caregivers of older adults with dementia: A scoping review. BMJ Open, 15(6), e095815. https://doi.org/10.1136/bmjopen-2024-095815 | Non-RCT |
| 481 | Nguyen, T. A., Pham, T., Nguyen, T. H., Nguyen, T. N., Tran, T. N., Phan, T. H., ... Gitlin, L. N. (2021). Advancing family dementia caregiver interventions in low- and middle-income countries: A trial of Resources for Advancing Alzheimer’s Caregiver Health in Vietnam (REACH VN). The American Journal of Geriatric Psychiatry, 29, 1105–1115. https://doi.org/10.1016/j.jagp.2020.026 | Non-RCT |
| 482 | Naylor, R., Spector, A., Fisher, E., Fucci, F., Bertrand, E., Marinho, V., Bomilcar, I., Coutinho, B., Laks, J., & Mograbi, D. C. (2024). Experiences of cognitive stimulation therapy (CST) in Brazil: A qualitative study of people with dementia and their caregivers. Aging & Mental Health, 28(2), 238–243. https://doi.org/10.1080/13607863.2023.2231376 | Non-RCT |

LMIC: low- and middle- income countries; RCT: randomised controlled trial

**Supplementary Table 2:** Table 2: Overview of study characteristics

| **Author** | **Year** | **Country** | **Intervention type** | **Intervention Duration** | **Overall sample size** | **Overall Age**  **(average years**  **1 d.p.)** | **Female**  **(1 d.p.)** | **Risk of Bias (RoB 2·0)** | **Intervention Details** |
| --- | --- | --- | --- | --- | --- | --- | --- | --- | --- |
| Ahmad and colleagues^32^ | 2024 | Malaysia | Telephone Psychoeducational | 12 weeks | 121 | 51.6 | N/A | Some concerns | The intervention was a telephone-delivered psychoeducational programme for dementia caregivers, based on the World Health Organization’s iSupport framework. Its aim was to improve caregiver knowledge, coping strategies, and ability to manage behavioural symptoms in people with dementia. Sixty participants were initially assigned to the intervention group, of whom 49 (81.7%) completed the study. The programme consisted of ten structured sessions delivered one-on-one by registered nurses and occupational therapists trained in the iSupport content. Sessions were conducted by telephone over a 12-week period (3 months), with each lasting approximately 30 minutes. Topics included understanding dementia, addressing caregiving challenges, managing caregiver stress, communication techniques, responding to behavioural symptoms, promoting positive interactions, problem-solving, accessing support services, self-care, and a review session. The content was standardised, with no tailoring to individual participants, and no modifications were made during the study. Intervention fidelity was supported by the use of a standardised session guide. Post-intervention assessments were conducted at week 14. The control group (n = 61, 56 completers; 91.8% retention) received usual care and information available through hospitals or clinics but did not receive any psychoeducational sessions. At baseline, the mean age (SD) of participants was 50.1 (12.4) years in the intervention group and 53.1 (12.9) years in the control group. |
| Arango-Lasprilla and colleagues^23^ | 2014 | Colombia | Group CBT class | 8 weeks | 69 | 57·5 | 81·2% | High | The intervention was the Coping with Frustration class, a cognitive–behavioral intervention developed by Gallagher-Thompson to help family caregivers manage negative emotions, such as anger and frustration, arising in the context of caregiving. The programme was delivered in a structured classroom format in small groups of 6 to 10 participants over 8 weeks, with one 2-hour session each week (total 8 sessions). Each session introduced a specific cognitive–behavioral strategy, including relaxation techniques, identification and challenging of dysfunctional thoughts, the use of positive self-statements, and assertiveness skills. Sessions were designed to present the coping strategy and then provide guided practice of the newly learned skill. Thirty-nine participants (mean age 59.4 years, SD 10.8) were assigned to the intervention group. The control group participated in an educational programme of equal duration (8 weeks) and time commitment (2 hours per week) as the experimental group. This programme included an attentional and educational component but did not provide practical application of cognitive–behavioral stress management skills. Content focused on dementia-related knowledge, including its history, course, and sequelae, and included two sessions viewing motion pictures depicting dementia and its effects. Thirty participants (mean age 55.1 years, SD 11.2) were assigned to the control group. |
| Baruah and colleagues^26^ | 2021 | India | Online skills training program | 12 weeks | 151 | 44·3 | 46·4% | Low | The intervention was the iSupport online training programme for caregivers of people with dementia, consisting of 23 lessons organised into five themes: (1) What is dementia? (one lesson), (2) Being a caregiver (four lessons), (3) Caring for me (three lessons), (4) Providing everyday care (five lessons), and (5) Dealing with behaviour changes (ten lessons). While an accompanying education-only (EO) e-book provided background information, the iSupport programme focused on interactive skill training for caregivers. Content was delivered online over a 3-month period, and participants were encouraged to complete all lessons within this timeframe. Seventy-four participants were assigned to the iSupport group, with 29 completing the study (mean age 46.5 years, SD 14.41). The control group received only the EO e-book programme, which was based on a brochure developed for caregivers of persons with dementia by Alzheimer’s Disease International (ADI) in collaboration with the World Health Organization. The e-book, presented in a website format, included information on basic dementia knowledge, living with and caring for a person with dementia, practical tips for managing dementia, personal and emotional stress of caring, caring for oneself, sources of help for the caregiver, and available support tools through ADI. It did not include interactive components or exercises for caregivers, as were present in iSupport. Seventy-seven participants were assigned to the control condition, with 26 completing the study (mean age 42.2 years, SD 11.9). |
| Chen and colleagues^33^ | 2024 | China | Group based programme: Music therapy, touch therapy, creative storytelling therapy, reminiscience therapy, cognitive training, mindfulness meditation, fine motor and visual attention exercises. | 8 weeks | 52 | 72.2 (Intervention);  74.58 (Control) | 35% (Intervention); 47% (Control) | High | The intervention was a multicomponent nonpharmacological intervention approach (MCNIA) that combined several therapeutic activities, including music therapy, touch therapy, and storytelling. The programme was delivered over a 2-month period, though exact session frequency and duration were not reported. The aim was to provide sensory, emotional, and cognitive stimulation to participants through structured non-drug activities. Twenty-seven participants were initially assigned to the MCNIA group, with 20 completing the study (mean age 72.2 years, SD 6.23). The control group received standard medical care without any nonpharmacological intervention. Twenty-five participants were initially assigned to the control group, with 19 completing the study (mean age 74.58 years, SD 7.88). |
| Danucalov and colleagues^42*^ | 2017 | Brazil | Yoga and compassion meditation | 8 weeks | 46 | 46·0 | 89·1% | Some concerns | The intervention was a yoga and compassion meditation programme delivered over an 8-week period. Participants attended three sessions per week, each lasting 1 hour and 15 minutes. The yoga practices were of low intensity. One of the three weekly sessions was conducted in person, while the remaining two sessions were completed at home using a provided instructional DVD to guide practice. The programme aimed to promote physical relaxation, mindfulness, and compassionate awareness. Twenty-five participants were assigned to the intervention group (mean age 55.5 years, SD 8.1). The control group was a non-treatment group; for ethical reasons, participants in this group were offered the intervention after the conclusion of the study. Twenty-one participants were assigned to the control group (mean age 53.4 years, SD 8.2). |
| Danucalov and colleagues^46*^ | 2013 | Brazil | Yoga and compassion meditation | 8 weeks | 46 | 46·0 | 89·1% | Some concerns | The intervention was a yoga and compassion meditation stress reduction programme delivered over 8 weeks (2 months). Participants engaged in three sessions per week, each lasting 1 hour and 15 minutes, for a total of 24 sessions. One session per week (8 total) was conducted live in person, while the remaining two weekly sessions (16 total) were performed at home with the aid of an instructional DVD. The programme combined low-intensity yoga postures, breathing exercises, meditational practices, and compassion meditation techniques. Twenty-five participants were assigned to the intervention group (mean age 55.5 years, SD 8.1). The control group received no treatment during the study period; however, for ethical reasons, participants in this group were offered the same intervention after study completion. Twenty-one participants were assigned to the control group (mean age 53.4 years, SD 8.2). |
| de Oliveira and colleagues^32^ | 2019 | Brazil | Tailored Activity Program | 12 weeks | 21 | 58·7 | 76·2% | Some concerns | The intervention was the Tailored Activity Program – Outpatient Version (TAP-O), adapted from the in-home TAP model developed by Gitlin et al., and designed for outpatients with dementia and their caregivers. Delivered over 3 months, TAP-O comprised eight sessions with an occupational therapist who assessed the patient’s abilities and interests, prescribed individually tailored activities, and educated caregivers about dementia and the integration of meaningful activities into the daily routine. The programme aimed to promote engagement, reduce behavioural symptoms, and support caregiver competence in activity implementation. Eleven participants were assigned to the intervention group (mean age 56.8 years, SD 15.0). The control group received regular care and participated in eight psychoeducation group sessions; ten participants were assigned to the control group (mean age 60.7 years, SD 16.4). |
| Dias and colleagues^27^ | 2008 | India | Home care education and support program | 24 weeks | 81 | 53·5 | 87·7% | Some concerns | The intervention was a flexible, stepped-care Home Care Program delivered at the participant’s home by a community care team. The programme included several core components carried out by a home care assistant (HCA): basic education about dementia (including its nature, course, and features); education on common behavioural problems and their management; caregiver support, such as assistance with activities of daily living for elderly caregivers living alone with the patient; referral to psychiatrists or the family doctor when behavioural problems were severe and warranted medication; facilitation of networking among families to form support groups; and advice on existing government schemes for older adults. The intervention duration was a minimum of 6 months. Forty-one participants were assigned to the intervention group (mean age 53.2 years, SD 14.0). The control group was a waitlist condition: participants received only education and information regarding dementia and were placed on a waiting list to receive the intervention after 6 months. They were free to access existing health services during the waiting period. Forty participants were assigned to the control group (mean age 53.8 years, SD 16.0). |
| Duru Aşiret, Kütmeç Yılmaz & Sayın Kasar^44^ | 2021 | Turkey | Education and Training | 16 weeks | 29 | 46·8 | 96·6% | Some concerns | The Dementia Care and Support Program (DCSP) was a 16-week multicomponent psychosocial intervention designed for family and informal caregivers of people with dementia. Its aims were to increase caregiver knowledge, improve skills and coping strategies, and provide emotional and practical support. The programme combined both individual and group elements, including home visits, telephone interviews, structured group training, face-to-face group meetings, and WhatsApp group communication. Caregivers who were unable to attend in-person group training sessions were supported through the WhatsApp group and additional home visits. The WhatsApp group facilitated ongoing communication between caregivers, peers, and researchers. During the 16-week period, caregivers were interviewed monthly, with four face-to-face interviews conducted with each patient–caregiver dyad. The structured training package, developed by the researchers, included videos, oral presentations, and question–answer sessions. Written study plans were provided to all participants, and in some sessions, a psychologist from the dementia clinic contributed by teaching stress management and problem-solving methods. The intervention also offered support for individual caregiver problems as they arose. Thirty participants were assigned to the intervention group (>53 years: 40%; ≤53 years: 58.1%). The control group received routine hospital care during the study period and was offered the DCSP after 16 weeks; 31 participants were assigned to the control group (>53 years: 60%; ≤53 years: 41.9%). |
| Gavrilova and colleagues^24^ | 2009 | Russia | Education and Training | 5 weeks | 53 | 59·3 | 75·5% | Low | The 10/66 Dementia Research Group Brief Caregiver Intervention was a five-week, home-delivered programme developed for low- and middle-income country settings with limited health and social care resources. Originally designed in India with input from the wider 10/66 group, the intervention was tailored to be deliverable using existing local resources and adaptable to cultural contexts. It aimed to provide basic dementia education and specific training for managing problem behaviours. The programme comprised three manualised modules: (i) assessment (one session), (ii) basic education about dementia (two sessions), and (iii) training regarding specific problem behaviours (two sessions). While it was intended to be delivered by Multi-Purpose Health Workers (MPHWs), in Moscow this was not feasible, and the intervention was instead delivered by newly qualified doctors with no prior experience working with people with dementia or their families. Thirty participants were allocated to and received the intervention; 25 were analysed (mean age 61.5 years for 30 randomised, 59.6 years for 25 analysed; mean years of education 17.6 and 18.0, respectively). The control group received usual medical care only and was placed on a waiting list for the intervention. Thirty participants were allocated to the control group, with 28 analysed (mean age 59.3 years for 30 randomised, 59.1 years for 28 analysed; mean years of education 12.8 and 13.3, respectively). |
| Ghaffari and colleagues^52^ | 2019 | Iran | Educational intervention | 8 weeks | 50 | 43·0 | 80·0% | Some concerns | The Group-Based Resilience Training Program was an eight-week, group-delivered educational intervention designed to promote resilience among caregivers. The programme was based on the resilience education projects of Henderson-Milstein and Kravets and aimed to enhance both personal and external supportive factors, as well as coping and problem-solving skills. Sessions were conducted once per week for eight weeks, each lasting 45 minutes, and were delivered in a hospital setting in Mazandaran Province, Iran, by one of the study researchers. The format included lectures supported by PowerPoint presentations and educational pamphlets. Educational content was structured into eight sequential sessions: (1) introductions and explanation of Alzheimer’s disease, resilience, and individual characteristics; (2) in-person supportive factors such as self-esteem and optimism; (3) external supportive factors and personal responsibility; (4) resilience promotion through commitment; (5) resilience promotion through control; (6) resilience promotion through challenge; (7) resilience promotion through coping; and (8) review and problem-solving. Twenty-five participants were assigned to the intervention group (mean age 42.6 years, SD 6.2). The control group received routine care, which included education on medicinal and non-medicinal care for patients but no structured resilience training during the study period; an educational resilience programme was offered to this group after study completion. Twenty-five participants were assigned to the control group (mean age 43.4 years, SD 6.3). |
| Guerra and colleagues^33^ | 2011 | Peru | Educational intervention | 5 weeks | 58 | 50·5 | 86·2% | Some concerns | The 10/66 Caregiver Intervention: Helping Carers to Care was a five-week, home-delivered programme developed in India with input from the wider 10/66 group, including experts from high-income countries. It was designed for low- and middle-income country settings with limited health and social care resources and aimed to provide basic dementia education and specific training on managing problem behaviours. The manualised programme comprised three modules delivered in five weekly sessions of approximately 30 minutes each. Module 1 (one session) focused on assessment, covering cognitive and functional impairments, the caregiver’s knowledge and understanding of dementia, care arrangements, and identification of behavioural problems that cause the greatest difficulty, along with perceived caregiver burden. Module 2 (two sessions) provided basic education, including a general introduction to dementia, expectations for the future, clarification of causes and myths, and locally available care and treatment options. Module 3 (two sessions) offered training on up to eight problem behaviours identified in the assessment, such as personal hygiene, dressing, incontinence, repeated questioning, clinging, aggression, wandering, and apathy. Twenty-nine participants were assigned to the intervention group (mean age 53.3 years, SD 15.9). The control group received care as usual at the local memory clinic and was placed on a waiting list to receive the intervention after six months. Twenty-nine participants were assigned to the control group (mean age 47.6 years, SD 15.0). Two participants in the intervention group died after randomisation, reducing the number completing the intervention assessment, although baseline characteristics were unaffected. |
| Heydari and colleagues^40^ | 2017 | Iran | Problem oriented coping strategies training | 8 weeks | 72 | 49·8 | 65·3% | Some concerns | The Problem-Oriented Coping Strategies Training was an eight-week educational intervention designed to enhance coping skills among caregivers. The programme was delivered in eight weekly sessions, each lasting 45 minutes, conducted one day per week. Session content included: primary evaluation, clinical interview, problem-solving, anger management, stress and adaptive coping strategies, coping with negative emotions and cognitive restructuring, strategies for building effective relationships, and a concluding session to review and consolidate learning. Thirty-six participants were assigned to the intervention group (mean age 52.44 years, SD 8.53). The control group received three educational sessions focused on Alzheimer’s disease. Thirty-six participants were assigned to the control group (mean age 47.13 years, SD 9.62). |
| Hinton and colleagues^29†^ | 2021 | Vietnam | Training sessions | 12 weeks | 51 | 58·8 | 86·3% | High | The REACH VN intervention was a culturally adapted version of the REACH VA programme, itself based on REACH II. It was delivered over a 3-month period to family caregivers of people with dementia. The intervention consisted of four “core” training sessions covering problem solving, mood management and cognitive restructuring, stress management techniques (e.g., signal breath, pleasant event scheduling), and communication skills. Up to two additional sessions were provided based on caregiver needs and clinical judgment. The adaptation process involved substantial modifications to the intervention manual and caregiver notebook to ensure cultural and linguistic appropriateness, including substituting culturally relevant examples, simplifying language, and expanding caregiver education about Alzheimer’s disease within sessions. Contextual changes included involving multiple family members when appropriate, engaging the male head of household during the initial session to facilitate participation and retention, and typically delivering sessions weekly rather than biweekly to maintain momentum. Training adaptations included supplementing standard REACH VA training with principles of Buddhism to enhance interventionist skills, and conducting a small case series for hands-on practice. All interventionists were female healthcare or allied professionals (nurses, physicians, and social workers). Home visits were conducted by pairs of interventionists, with one in the lead role and the other in a supportive role. Twenty-five participants were assigned to the intervention group (mean age 59.0 years, SD 10.4).  The control group received an enhanced control condition consisting of a single face-to-face home visit (or at another location of the caregiver’s choice) at enrolment, which focused on education about the nature of dementia and provision of written educational materials. Any safety issues identified during the visit were addressed by research staff. Twenty-six participants were assigned to the control group (mean age 58.7 years, SD 13.9). |
| Kamkhagi and colleagues^34^ | 2015 | Brazil | Psychodynamic group therapy | 14 weeks | 37 | 59·2 | 73·0% | High | The Psychodynamic Group Psychotherapy (PGT) intervention was delivered once weekly for 14 weeks, with each of the 14 sessions lasting 90 minutes. Groups were facilitated by two experienced psychologists. Discussion topics most frequently raised by participants—spontaneously or prompted by facilitators—included feelings of loneliness and helplessness associated with caregiving, family conflicts, and changes in family roles. Facilitators encouraged participants to explore and articulate feelings of sadness, frustration, anger, and guilt, and to reflect on their relationship with the care recipient before dementia onset and in its current form. This process aimed to help caregivers develop new ways of maintaining an affectionate relationship with the person they care for. Subsequent sessions explored caregivers’ prior roles in the family and the adaptations required due to the illness, including taking on unwanted roles or reducing involvement in other activities. Facilitators worked to establish empathy with the caregivers’ challenges and discussed coping strategies for sources of burden. Towards programme completion, participants discussed the lack of recognition for their efforts from other family members and were encouraged to articulate a “caregiver declaration of rights” to affirm their value and needs. Twenty participants were assigned to the PGT group (mean age 62.1 years, SD 9.9).  The Psychodynamic Group Psychotherapy (PGT) intervention was delivered once weekly for 14 weeks, with each of the 14 sessions lasting 90 minutes. Groups were facilitated by two experienced psychologists. Discussion topics most frequently raised by participants—spontaneously or prompted by facilitators—included feelings of loneliness and helplessness associated with caregiving, family conflicts, and changes in family roles. Facilitators encouraged participants to explore and articulate feelings of sadness, frustration, anger, and guilt, and to reflect on their relationship with the care recipient before dementia onset and in its current form. This process aimed to help caregivers develop new ways of maintaining an affectionate relationship with the person they care for. Subsequent sessions explored caregivers’ prior roles in the family and the adaptations required due to the illness, including taking on unwanted roles or reducing involvement in other activities. Facilitators worked to establish empathy with the caregivers’ challenges and discussed coping strategies for sources of burden. Towards programme completion, participants discussed the lack of recognition for their efforts from other family members and were encouraged to articulate a “caregiver declaration of rights” to affirm their value and needs. Twenty participants were assigned to the PGT group (mean age 62.1 years, SD 9.9). |
| Lök and Bademli^35^ | 2017 | Turkey | Educational and supportive program | 7 weeks | 40 | 52·4 | 67·5% | High | The “First You Should Get Stronger” caregiving programme was a structured seven-week intervention aimed at strengthening caregivers, reducing caregiving burden, and promoting healthy lifestyle behaviours. The programme consisted of seven weekly sessions, each lasting 45 minutes. While specific content details were not reported, the sessions were designed to provide education, skills training, and motivational support to enhance caregiver resilience and wellbeing. Twenty participants were assigned to the intervention group (mean age 52.97 years, SD 8.19). The control group’s activities were not reported; however, 20 participants were assigned to this group (mean age 51.76 years, SD 6.46). |
| Mahdavi and colleagues^81^ | 2017 | Iran | Spiritual therapy (group) | 5 weeks | 89 | 53·0 | NA | Some concerns | The Group Spiritual Therapy programme was delivered over five consecutive weeks, with one session per week. Each session lasted between 45 and 60 minutes. Session content was structured as follows: Session 1 – introduction and formation of group solidarity; Session 2 – the role of reciting the Quran in achieving personal peace; Session 3 – the role of recommended recitals and their repetition in promoting personal peace; Session 4 – the role of reciting prayers in fostering personal peace; and Session 5 – sharing experiences of spiritual care, reflecting on its effects, and preparing members to leave the group while applying the group’s benefits in daily life. Thirty-three participants were assigned to the intervention group, with data available for 30 participants.  Control Group 1 met weekly for five sessions of 45–60 minutes each, in which caregivers gathered in groups to discuss daily issues but no specific therapeutic intervention was delivered. Thirty-two participants were assigned to Control Group 1, with data available for 28 participants. Homogeneity tests indicated that groups were statistically matched on predisposing and confounding variables, with the majority of participants female and a mean age across groups of 52.96 years (SD 13.02). |
| Nguyen and colleagues^34^ | 2025 | Vietnam | Training sessions | 6 months | 350 | 61.7 | 70.6% | Some concerns | The REACH VN programme was a multicomponent caregiver education and support intervention delivered over one to three months, consisting of up to six weekly or biweekly sessions followed by maintenance calls. The intervention included a needs assessment, caregiver skills training, and stress management strategies, with the goal of enhancing caregiver competence and reducing burden. Content covered problem solving, behavioural management, communication, and self-care techniques. Delivery took place in the home setting, with a flexible schedule adapted to the caregiver’s circumstances. One hundred seventy-five participants were assigned to the intervention group (mean age 61.9 years, SD 10.9).  The enhanced control group received a single in-home session of dementia education, which included information about the disease, its progression, and general caregiving principles. One hundred seventy-five participants were assigned to the control group (mean age 61.4 years, SD 11.3). Follow-up assessments for both groups were conducted at three and six months. |
| Novelli and colleagues^28^ | 2018 | Brazil | In-home activity dyad intervention | 16 weeks | 30 | 66·2 | 83·3% | Some concerns | The Tailored Activity Program – Portuguese Version for Brazil (TAP-BR) was an in-home activity intervention adapted from the original TAP model shown effective in reducing behavioural and psychological symptoms of dementia (BPSD) in the United States. The programme consisted of eight sessions delivered by occupational therapists over a 3–4 month period. Intervention delivery began with a systematic assessment of the interests and abilities of the person with dementia, the caregiver’s willingness and capacity to use activities, and the physical environment. Based on this assessment, three activities were developed to match the person’s preserved abilities rather than introduce new learning, with the aim of maximising participation and engagement. Caregivers received training on setting up and using the tailored activities in daily care, along with education about dementia and the nature of behavioural symptoms (e.g., behaviours are not intentional). Fifteen participants were assigned to the TAP-BR group (mean age 64.33 years, SD 6.76).  The control group was a waitlist condition in which participants received usual care during the study period and were offered the TAP-BR programme afterwards. Fifteen participants were assigned to the control group (mean age 68.16 years, SD 12.61). |
| Pahlavanzadeh and colleagues^31^ | 2010 | Iran | Group family education program | 5 weeks | 50 | 44·7 | 76·0% | High | The Family Education Program was a five-week caregiver education intervention delivered through weekly group sessions. Each session lasted 90 minutes and combined lecturing, group discussions (10–15 participants per group), and question-and-answer segments. Participants also received CDs containing recordings of all sessions for review. The programme content was based on an educational pamphlet prepared and edited by the researchers after reviewing relevant books and articles. The final content was validated and approved by a panel of 10 experts, including psychiatrists, a neurologist, and faculty members from the School of Nursing and Midwifery at Isfahan University of Medical Sciences. Twenty-five participants were assigned to the intervention group (mean age 42.88 years, SD 15.13).  The control group did not participate in the family education programme and received no additional intervention beyond usual circumstances. Twenty-five participants were assigned to the control group (mean age 46.56 years, SD 14.51). |
| Pakong and colleagues^53^ | 2018 | Thailand | Education and training program (group and individual) | 7 weeks | 72 | 55·4 | 77·8% | Some concerns | The Enhancing the Positive Aspects of Caregiving Program (EPACP) was delivered alongside usual care and was designed to increase caregiving self-efficacy, spirituality, and social support, while maintaining positive caregiving experiences for family caregivers of people with dementia. Developed based on Kramer’s two-factor adaptation model, the programme incorporated strategies such as information provision and skills training, exploration of spirituality, identifying positive events in caregivers’ lives, mindfulness practice, positive reappraisal activities, and goal setting. Group discussions and training were supplemented by individual counselling. Supporting materials included a dementia caregiving booklet, role model examples, and a video resource. The intervention consisted of six group sessions and one individual session. Three groups of 15 family members met once weekly for 2 hours over six weeks, followed in week 7 by an individual session delivered by the principal investigator. Thirty-six participants were assigned to the intervention group (mean age 55.11 years, SD 13.15).  The control group received only usual care, which included health education or suggestions from nurses and other healthcare professionals either at psychiatric clinics, in hospitals, or at home. Thirty-six participants were assigned to the control group (mean age 55.66 years, SD 10.89). |
| Pan and Chen^50^ | 2019 | China | CBT | 20 weeks | 112 | 62·7 | 62·5% | High | The Cognitive Behavioural Intervention was a five-month programme consisting of five monthly, 60-minute, face-to-face individual sessions, each followed by a 20–30 minute telephone consultation. The face-to-face sessions were delivered early in the month by nurse interventionists and were based on cognitive behavioural therapy (CBT) strategies recommended by Aboulafia Brakha et al. (2014), Cheng et al. (2017), and Schinköthe and Wilz (2014). The five-module programme was culturally tailored for family caregivers of people with dementia, with content flexible to individual needs. Telephone consultations were used to obtain participant feedback, reinforce strategies taught in the sessions, and answer questions. Fifty-six participants were assigned to the intervention group (mean age 63.3 years, SD 11.2).  The control group received general, unstructured conversations with nurse interventionists of 5–10 minutes in length, once monthly for five months. Conversations took place in participants’ homes, in hospital units during medical visits, or by telephone, and focused casually on daily life and health. Interventionists responded naturally and with concern but did not provide any structured instruction or strategies. Fifty-six participants were assigned to the control group (mean age 62.1 years, SD 10.6). |
| Pandya^25^ | 2019 | India and Nepal | Meditation program | 5 years | 185 | 52·6 | 83·8% | High | The Meditation Program Intervention was delivered over a five-year period and consisted of a 45-minute guided lesson once per week, conducted by trained instructors. Participants were also expected to practice at home once per week prior to the next class, with instructors maintaining a record of home practice. Each session followed a structured sequence of nine components: (1) centering in silence (5 minutes); (2) instant relaxation through isometric contraction of muscles in the supine position (5 minutes); (3) tree posture or standing still and centering in tree posture (5 minutes); (4) half-wheel posture and centering in half-wheel posture (5 minutes); (5) forward-bending posture and centering in forward-bending posture (5 minutes); (6) backward-bending posture and centering in backward-bending posture (5 minutes); (7) deep relaxation and slow breathing in a comfortable sitting position with silence, concentration, and centering (5 minutes); (8) deep relaxation and slow breathing in the supine position with silence, concentration, and centering (5 minutes); and (9) closing moments of silence (5 minutes). Ninety-six participants were assigned to the intervention group (mean age 52.68 years, SD 11.03).  The control group received no intervention during the study period and functioned as a wait-list comparison, with no alternative treatment or structured activities provided. Eighty-nine participants were assigned to the control group (mean age 52.50 years, SD 10.67). |
| Salehinejad and colleagues^37^ | 2022 | Iran | Education and training, psychoeducation, relaxation techniques (group and individual) | 5 weeks | 50 | ≤40 n=16 (32%)  41–50 n=19 (38%)  ≥51 n=15 (30%)^‡^ | 62·0% | Some concerns | The Compassion-Based Programme was a five-week, online intervention for family caregivers, delivered via weekly sessions presented by a nurse under the supervision of a psychiatrist. Each week, the session content was sent to participants. The programme comprised five structured modules: (1) establishing initial communication with caregivers, introducing the principles of a compassion-based programme, and exploring the concept of compassion; (2) mindfulness training, body scanning, and breathing exercises; (3) familiarisation with the principles of compassion; (4) training in compassion skills; and (5) training to accept problems and future challenges, cope with difficulties, and write reflective letters. Thirty-five participants were assigned to the intervention group (mean age 45.48 years, SD 17.36).  The control group was described only as a control condition during the study period. After follow-up assessments, participants in the control group were provided with the programme content along with videos and educational booklets. Thirty-five participants were assigned to the control group (mean age 43.35 years, SD 8.56). |
| Sanchez and colleagues^38^ | 2020 | Brazil | Psychoeducation and mindfulness (individual) | 8 weeks | 29 | 54·6 | 93·1% | High | The Web-Based Information Intervention was a fully automated, password-protected educational website designed for dementia caregivers and accessible on mobile devices, tablets, or desktop computers. At baseline, participants received a 20-minute training session on website use, along with a username and password. Educational content, developed by an expert group, was organised into 12 interactive sessions delivered over two months, with one or two sessions to be validated each week before unlocking the next. The content was divided into four sections: (1) text and image-based information on dementia, behavioural disorders, self-care for caregivers, and guidance for providing better care; (2) videos on relaxation training, practical care training, and experiences of other caregivers; (3) updates on important events and news; and (4) an online forum for information sharing, peer interaction, and questions, available without time restriction. The platform also featured a chat room for caregiver communication and weekly one or two educational sessions of 15–30 minutes. Twenty-five participants were assigned to the intervention group (≤40 years: n=7, 28%; 41–50 years: n=10, 40%; ≥51 years: n=8, 32%).  The control group received usual care, which consisted of illness information provided during routine semi-annual follow-up appointments with a neurologist. Twenty-five participants were assigned to the control group (≤40 years: n=9, 36%; 41–50 years: n=9, 36%; ≥51 years: n=7, 28%). In both groups, most caregivers were female, aged 41–50 years, married, unemployed, and held a postgraduate degree (51%). The lowest education level was primary school (12%). More than half of participants earned between 10 and 30 million RLS per month (minimum monthly wage at recruitment: 8,500,000 RLS), 62% of patients required nearly 24-hour care, and 32% had been receiving family care for more than three years. Only 29% of caregivers had internet access, with 70% connecting via mobile phones. |
| Shata and colleagues^30^ | 2017 | Egypt | Psychosocial group intervention (psychoeducation, brief group CBT, and group support sessions) | 8 weeks | 114 | 48·6 | 65·8% | Some concerns | The Group Psychosocial Intervention was delivered over an eight-week period and comprised three components: (1) two sessions of group psychoeducation, (2) six sessions of brief group cognitive behavioural therapy (CBT), and (3) ongoing group support sessions covering a variety of topics throughout the programme duration. Each session lasted between 45 and 60 minutes. The intervention aimed to provide education, psychological skills, and peer support to caregivers. Sixty participants were allocated to the intervention group, with data available for 55 participants (mean age 49.35 years, SD 11.89).  The control group received no intervention during the study period. Sixty participants were allocated to the control group, with data available for 59 participants (mean age 47.97 years, SD 12.76). |
| Söylemez and colleagues^41^ | 2016 | Turkey | In-home activity dyad intervention | 12 weeks | 70 | 61·7 | 72·9% | Some concerns | The Progressively Lowered Stress Threshold (PLST) Model – Psychoeducational Intervention was a home-based programme delivered to individuals with dementia and their caregivers over a three-month intervention phase, with follow-up three months later. Participants received four home visits over a six-month period, each lasting at least one hour. The first home visit focused on understanding the behaviours of the person with dementia, how caregivers coped with these behaviours, and the challenges they experienced. Two weeks later, during the second visit, the PLST-based intervention was provided and demonstrated. At the third visit, held two months later, the care plan was reviewed, additional techniques were taught, and caregivers received written materials summarising the care plan. Following this visit, follow-up telephone calls were made every two weeks. Caregivers also received a booklet prepared by the authors containing educational and support materials, including general information about dementia, common problems such as sleep disturbances, guidance on administering medications, bathing, toileting, physical activities, nutrition, and communication, as well as recommended readings for family members. Thirty-five participants were assigned to the intervention group (mean age 61.2 years, SD 13.14).  The control group received a routine care programme, in which caregivers were informed about the disease by physicians following diagnosis and given leaflets. Caregivers accompanying patients to outpatient visits could ask physicians questions, with visit frequency ranging from every six months to once per year. Participants in this group also received four home visits over a six-month period, each lasting about one hour. The first visit reviewed study aims and protocols, obtained informed consent, and collected baseline data. Two weeks later, the second visit provided general dementia care information and updated leaflets. Data were collected at the third home visit two months later and again at the final home visit three months later. After the study concluded, participants in the control group were provided with the PLST intervention materials. Thirty-five participants were assigned to the control group (mean age 62.28 years, SD 12.76). |
| Taati and colleagues^48^ | 2016 | Iran | Support group | 8 weeks | 62 | 55·0 | 90·3% | High | The Support Group Intervention was an eight-week programme for family caregivers of people with Alzheimer’s disease, preceded by a one-week information session. Sessions were held once per week in a building separate from the Alzheimer’s Association of Iran, where caregivers typically attended for services and patient visits. The intervention aimed to reduce depression, anxiety, and stress by providing a structured group environment for emotional support, sharing experiences, and discussing strategies for coping with caregiving challenges. Group meetings were facilitated in person, though the specific session format and content were not described in the source. Caregivers were recruited via convenience sampling from those attending the Alzheimer’s Association. |
| Tran and colleagues^54†^ | 2022 | Vietnam | Training sessions | 12 weeks | 51 | 58·8 | 86·3% | High | The Mindfulness Intervention Group (MIG) was an eight-session, home-based mindfulness programme delivered once per week over two months. Each session focused on a specific mindfulness practice: (1) body and breathing meditation to stabilise the mind; (2) body exploration meditation to distinguish between thinking about a sensation and directly experiencing it without judgment; (3) reflection on prior experiences combined with attentive yoga movement to explore physical and mental limits; (4) sounds and thoughts meditation to recognise thoughts as transient mental events; (5) exploring difficulties meditation to address obstacles as they arise; (6) friendship meditation to cultivate tenderness and compassion, dispelling negative thoughts; (7) critical reflection on the relationship between routine, activities, behaviour, and mood to encourage beneficial choices; and (8) integration of full attention (mindfulness) into daily life as a lasting resource. Eleven participants were assigned to the MIG (mean age 51.6 years, SD 11.9).  The Active Control Group (ACG) received a two-hour home visit by a trained nurse researcher, during which they were provided with an educational guideline booklet on dementia, covering topics such as risk factors, diagnosis, care measures, emotional impacts, stress, daily life changes, and treatment. The visit also included answering any caregiver questions. Participants were instructed to maintain their usual activities and were placed on a waiting list to participate in the mindfulness programme after the trial. In week 8, the researcher contacted control group participants by phone to ensure adherence to the instructions. Eighteen participants were assigned to the ACG (mean age 56.4 years, SD 18.8). Across both groups, most participants were female and co-resided with the person receiving care, with no statistically significant sociodemographic differences between groups. |
| Uyar and colleagues^36^ | 2019 | Turkey | Individual and group psychosocial support program | 8 weeks | 61 | 53·6 | 78·7% | Some concerns | The Support Group intervention was delivered over eight weeks in weekly sessions lasting 1.5–2 hours. In addition to active peer support among caregivers, participants received structured input from a multidisciplinary team consisting of three geriatric nurses, a psychologist, a resident in geriatrics, a general practitioner, and a social worker. The psychologist provided guidance on communication with people with Alzheimer’s disease and strategies for managing and controlling behavioural problems. The nurses gave general information about Alzheimer’s disease, caregiving skills, and caregiver self-care, and facilitated group discussions to address and evaluate relevant issues. The social worker informed caregivers about available support services through the Alzheimer’s Association (e.g., pressure-relief mattresses, wheelchairs) and connected caregivers to appropriate resources when needed. The geriatric resident and general practitioner answered questions about disease management, medications, potential side effects, and strategies to minimise adverse effects. All members of the expert team participated in every session, engaging in discussions and responding to questions within their areas of expertise. Each session began with a review of previous topics, followed by discussion of the current theme with caregiver participation and the provision of targeted training. Thirty-two participants were assigned to the intervention group (mean age 52.50 years, SD 8.57).  The control group received routine services and no structured intervention. Thirty participants were assigned to the control group (mean age 57.67 years, SD 9.41). |
| Wang, Chien and Lee^43^ | 2012 | Mainland China | Mutual support group – education and skills training | 24 months | 78 | 18–30 = 27  31–50= 33  51–70= 18  39·40^§^ | 61·5% | Some concerns | The Mutual Support Group intervention was designed for Chinese family caregivers of a relative with dementia and delivered over a 24-months period between January 2010 and March 2011. Participants met in small groups at dementia resource and respite care centres in Guangzhou, Guangdong Province. The sessions provided a structured opportunity for caregivers to share experiences, exchange practical strategies, and offer mutual emotional support, while also receiving targeted information on dementia care. Group discussions were facilitated to address common caregiving challenges, coping methods, and available community resources, with the aim of improving caregiver wellbeing and competence. Seventy-eight family caregivers meeting DSM-IV dementia caregiving criteria were recruited and randomised from a pool of 350 eligible individuals.  The control group received standard family care available in the community, without participation in structured support groups. This included routine family-based caregiving and access to any publicly available services, but no additional organised programme. |
| Xie and colleagues^35^ | 2024 | China | Internet based support | 6 months | 72 | 57.7 | 86% | Some concerns | The Web-Based Caregiver Training programme was a six-month online intervention providing dementia caregivers with training and resources across multiple domains. Delivered via a dedicated web platform, the content covered dementia knowledge, care skills, safety, language training, memory stimulation, and music therapy. The platform was accessible to participants throughout the intervention period, allowing flexible, self-paced learning. Thirty-three participants were assigned to the intervention group (mean age 58.79 years, SD 4.08).  The control group received routine care, which included a dementia education booklet and face-to-face support every three months. They were placed on a waitlist to receive access to the web-based training after the six-month follow-up period. Thirty-three participants were assigned to the control group (mean age 56.70 years, SD 3.47). |
| Yang and colleagues^36^ | 2024 | China | Online group logotherapy | 7 months | 13 | 63.7 | 61.6% | High | The Online Group Logotherapy programme was delivered over a two-month period (September–October 2022) and consisted of eight sessions conducted in an online group format. The intervention was grounded in logotherapy principles, focusing on experiential, creative, and attitudinal pathways to meaning. Sessions were designed to help participants explore personal values, discover purpose, and develop adaptive attitudes toward life challenges, including those associated with caregiving. Thirteen participants took part in the intervention (mean age 63.7 years, SD 13.0). This was a pre-experimental study with no control group. |
| Yazdanmanesh and colleagues^39^ | 2023 | Iran | Education and psychoeducation – group in person | 5 weeks | 72 | 46·6 | 73·6% | High | The Mutual Support Group intervention was delivered over 24 weeks and consisted of 12 bi-weekly, 1.5-hour peer-led group sessions facilitated by an advanced practice psychiatric nurse experienced in dementia care and group facilitation. The intervention was structured around a group protocol developed for the study, drawing on evidence from previous mutual support interventions. Seven core themes guided the sessions: (1) information about the client’s condition; (2) development of the group as a support system; (3) emotional impact of caregiving; (4) learning about self-care; (5) improvement of interpersonal relationships; (6) establishing support outside the group; and (7) improvement of home care skills. A structured problem-solving strategy was incorporated into each session, whereby at least one caregiver shared a personal caregiving problem with the group, which was addressed using a six-step model (problem definition, generation of alternatives, evaluation of alternatives, cognitive rehearsal of the action plan, homework execution, and evaluation of outcomes).  Thirty-nine participants were allocated to the intervention group (mean age 39.19 years, SD 13.9), while 39 participants were allocated to the control group (mean age 39.62 years, SD 13.68).  The control group received routine family services provided by the dementia centers, which included monthly medical consultation and advice on the client’s condition, treatment plans, and medication effects from visiting doctors; financial aid and social welfare referrals from a social worker; dementia care educational talks from registered nurses; and general referrals to medical and social services by center staff. In rural or underserved areas, available services were limited to medical consultations and hospital referrals. After post-test assessments, the control group was offered the opportunity to participate in the mutual support program. |
| Zarepour and colleagues^47^ | 2020 | Iran | Educational programme: training sessions and book | 4 weeks | 70 | 46·8 | 70·0% | Some concerns | The Educational Intervention and Educational Book program was delivered over a 4-week period and consisted of seven 1.5-hour sessions focused on caring for an older person with dementia and caregiver self-care. Sessions were delivered via lectures and educational aids such as PowerPoint, video projection, video players, and educational imagery. Specialist content was taught by senior lecturers, neurologists, and researchers in their respective fields. To reinforce learning, the researcher maintained ongoing contact with participants throughout the 4-week program, making themselves available for advisory services via telephone, SMS, video calls, or virtual messaging programs, three hours daily (18:00–21:00). Caregivers could seek advice on routine care issues and were advised to consult a specialist if the patient required advanced or specialized care.  Thirty-five participants were allocated to the intervention group.  The control group (n = 35) received conventional care with no formal education on dementia care during the study period. Following study completion, the educational sessions were delivered to the control group, and each participant received an educational book. |

^*^Two reports from same study; ^†^Two reports from same study; ^‡^ Calculation of average age not possible; ^§^Calculation based on frequency table

CBT = Cognitive Behavioural Therapy; d.p decimal place; NA = Not applicable; RoB = Cochrane Collaboration's Risk of Bias 2·0

**Supplementary Material 1: Database Search Strategies**

Search terms were grouped according to the PICO framework: ‘supportive interventions’ (phenomena of interest) for ‘informal caregivers’ of people affected by ‘dementia’ in LMICs. For the LMICs field of search terms, both countries’ endonyms and exonyms were used, alongside former names, regional terms, and the names of LMICs not universally recognised e.g., Palestine (West Bank and Gaza appear on the World Bank’s list of lower-middle income economies). This was in accordance with an evaluation of the Cochrane Group’s LMIC Filters 2020.^43^ Taiwan originally featured as a search term but papers from Taiwan were later excluded since the World Bank lists it separately from China as a high-income economy. The search strategy was adapted to account for database differences, as not all databases accepted wildcards and truncations or were limited in the number of these, or by search terms they could accept within a search string. In databases or journals which did not support advanced searches, or multiple search terms and the use of Boolean operators, the most pertinent search terms, such as ‘dementia’ were used. Where available, limits were placed to include human studies only and no limitations were placed on date or language (if full texts were unavailable in English, they were later screened out at this stage). Medical Subject Headings were used where available.

**Search summary 1**

**MEDLINE**

| 1 | Cognitive Dysfunction/ | 34887 |
| --- | --- | --- |
| 2 | Neurodegenerative Diseases/ | 25318 |
| 3 | exp Frontotemporal Dementia/ or "Mental Status and Dementia Tests"/ or dementia, multi-infarct/ or exp dementia/ or aids dementia complex/ or alzheimer disease/ or exp aphasia, primary progressive/ or creutzfeldt-jakob syndrome/ or exp dementia, vascular/ or diffuse neurofibrillary tangles with calcification/ or exp frontotemporal lobar degeneration/ or huntington disease/ or kluver-bucy syndrome/ or lewy body disease/ or "Pick Disease of the Brain"/ or Cerebral Amyloid Angiopathy/ or cognition disorders/ or cognitive dysfunction/ | 279666 |
| 4 | (senil* or dementia* or "lewy bod*" or presenil* or alzheimer* or "cogniti* dis*" or "neurocogniti* dis*" or "cogniti* impair*" or "cogniti* defect*" or neurodegenerative or "delirium, dementia, amnestic, cognitive disorders" or "presenile dementia").m_titl. | 183742 |
| 5 | 1 or 2 or 3 or 4 | 335816 |
| 6 | Caregivers/ or family/ or adult children/ or family relations/ or Friends/ | 143641 |
| 7 | ("famil* caregiver*" or caregiv* or famil* or relative* or "famil* care*" or "famil* support" or "famil* member*" or neighbo$r* or carer* or friend* or "informal caregiv*" or care* or caring or family or "adult children" or "child adult*" or "adult child*" or "sandwich carer*" or "sandwich caregiv*" or "family relations" or parent* or father* or spouse* or partner* or husband* or wife or wives or son* or daughter* or offspring* or sibling* or brother* or sister*).m_titl. | 1207740 |
| 8 | 6 or 7 | 1265291 |
| 9 | Psychosocial Intervention/ or psychosocial support systems/ or internet-based intervention/ or social media/ or exp Cognitive Behavioral Therapy/ or social support/ or counseling/ or directive counseling/ or pastoral care/ or complementary therapies/ or holistic health/ or mind-body therapies/ or sensory art therapies/ or spiritual therapies/ or aromatherapy/ or art therapy/ or color therapy/ or dance therapy/ or music therapy/ or play therapy/ | 208090 |
| 10 | ("support* intervention*" or intervention* or therap* or engage* or therap* or program* or manage* or strateg* or polic* or support* or "non pharmacological" or "non-pharmacological" or "nonpharmacological" or "cognitive therapy" or "cognitive behavioural therapy" or CBT or "cognit* psychotherap*" or psychosocial or pyschoeducation* or psycho-education* or psycho* or social or "social support*" or befriend* or counsel* or counsel$ing or "directive counsel$ing" or "distance counsel$ing" or "alternative therap*" or "traditional therap*" or "doll therap*" or "pet* therap*" or "sensory therap*" or massage or "advance* care plan*" or "advance* plan*" or "decision* making aid*" or "decision* aid*" or "decision* support*" or "person* cent* care" or "patient* cent* care" or "dementia care" or "train*" or inform* or educat* or teach* or advis* or advice* or "education* intervention*" or resource* or "dementia training" or "dementia aware*" or "caregiver* education" or "caregiver* training" or "health education" or "peer-support" or "peer support network*" or "internet-based intervention*" or "social media" or pastoral or spiritual or religio* or "sensory art therap*" or "spiritual therap*" or "mind-body therap*" or "music therap*" or "art therap*" or aromatherap* or "dance therap*" or self-help or selfhelp).m_titl. | 3100379 |
| 11 | 9 or 10 | 3195407 |
| 12 | Developing Countries/ | 80754 |
| 13 | (LMIC or LMICS or "lower middle-income countr*" or Lower-MIC or "low income countr*" or "low-income countr*" or LICs or "middle income countr*" or "upper middle-income countr*" or Upper-MIC or "developing countr*" or "developing nation*" or underdeveloped or under-developed or underserved or under-served or "emergent nation*" or "transitional countr*" or LAMI or "LAMI countr*" or "low and middle-income countr*" or "South Pacific" or "South Asia*" or "SE Asia*" or Angola or Algeria or Numidia or al-Jaza'ir or Bangladesh or Benin or Bhutan or "Druk Yul" or Bolivia or Buliwya or Wuliwya or Volivia or Cambodia or Kampuchea or Cameroon or Cameroun or Cameroon or "Cape Verde" or "Cabo Verde" or Comoros or Komori or "Juzur al-Qamar" or Comores or "Congo, Rep." or "Democratic Republic of Congo" or Zaire or "Republique du Congo" or "Repubilika ya Kongo" or "Republiki ya Kongo" or "Cote d'Ivoire" or "Ivory Coast" or Djibouti or Jibuti or Djibouti or Jabuuti or Gabuuti or "Egypt, Arab Rep." or Misr or Masr or "El Salvador" or Eswatini or Swaziland or eSwatini or Ghana or Gaana or Gana or Honduras or India or Bharot or Bharat or Bharata or Bharatam or Bharatadesam or Kenya or Kiribati or "Kyrgyz Republic" or Kyrgyzstan or Kirgizija or "Lao PDR" or Lao or Laos or Lesotho or Mauritania or Muritan or Agawec or Muritanya or "Micronesia, Fed. Sts.Tunisia" or "Federated States of Micronesia" or Moldova or Moldavia or Mongolia or "Mongol Uls" or Morocco or Amerruk or Elmeyrib or Al-mayreb or Myanmar or Burma or Myanma or Nepal or Nicaragua or Nigeria or Nijeriya or Naijiriya or Naijiria or Pakistan or "Papua New Guinea" or "Papua Niugini" or "Papua Niu Gini" or Philippines or Pilipinas or "Sao Tome and Principe" or "Sao Tome e Principe" or Senegal or Senegaal or "Solomon Islands" or "Solomon Aelan" or "Sri Lanka" or Tanzania or "Tanganyika and Zanzibar, German East Africa" or Timor-Leste or "East Timor" or "Timor Lorosa'e" or Ukraine or Ukrajina or Uzbekistan or O'zbekiston or Vanuatu or Vietnam or "Viet Nam" or "South Vietnam" or "Cochin-China (south)" or "Cochin-China" or "Annam (central)" or Annam or "Tonkin (north)" or Tonkin or "West Bank and Gaza" or "The State of Palestine" or Palestine or Zambia or "Northern Rhodesia" or Zimbabwe or "Southern Rhodesia" or "East Pakistan" or Rhodesia or Yugoslavia or Himalayan or Himalyan or Sahel or Yucatan or Palestine or Palestinians or Rohingya or Michoacan* or Mixtec* or Zapotec* or Afghanistan or Afghanestan or "Burkina Faso" or "Upper Volta" or Burundi or Uburundi or "Central African Republic" or "Central Africa Republic, Chad" or Chad or "French Equatorial Africa" or Centrafrique or Beafrika or "Congo, Dem. Republic" or "Republique democratique du Congo" or "Republiki ya Kongo Demokratiki" or "Repubilika ya Kongo ya Dimokalasi" or "Jamhuri ya Kidemokrasia ya Kongo" or Eritrea or Iritriya or Ertra or Ethiopia or Abyssinia or Ityop'ia or "Gambia, The" or "The Gambia" or Guinea or Guinee or Gine or Guinea-Bissau or Guine-Bissau or Haiti or Ayiti or "Korea, Dem. People's Rep." or "Korea (North and South)" or North Korea or Choson or Bukchoson or Liberia or Madagascar or Madagasikara or Malawi or Mali or "Sudanese Republic" or Mozambique or Mocambique or Niger or Rwanda or "Rwanda and Burundi" or "German East Africa" or "Sierra Leone" or Somalia or Soomaaliya or as-Sumal or "South Sudan" or "Sudan Kusini" or "Paguot Thudan" or Sudan or As-Sudan or "Syrian Arab Republic" or Syria or Suriyah or Tajikistan or Tojikistan or Togo or Uganda or "Yemen, Rep." or Al-Yaman or "Republic of Yemen" or Albania or Shqiperi or Shqiperia or "American Samoa" or "Amerika Samoa" or Argentina or Armenia or Hayastan or Azerbaijan or Azerbaycan or Belarus or Bielarus or Belize or "Bosnia and Herzegovina" or "Bosna i Hercegovina" or Botswana or Brazil or Brasil or Bulgaria or Balgarija or Bulgariya or China or "China (north)" or Cathay or "China (south)" or Mangi or Zhongguo or Zhongguo or Colombia or "Costa Rica" or Cuba or Dominica or "Dominican Republic" or "Republica Dominicana" or Ecuador or "Equatorial Guinea" or "Guinea Ecuatorial" or "Guinee equatoriale" or "Guine Equatorial" or Fiji or Viti or Gabon or "Republique gabonaise" or Georgia or Sakartvelo or Sak'art'velo or Grenada or Guatemala or Guyana or Indonesia or "Iran, Islamic Rep." or Persia or Iran or Iraq or Mesopotamia or Al-'Iraq or Eraq or Jamaica or Jordan or "Al Urdun" or Al-'Urdun or Kazakhstan or Qazaqstan or Kazakhstan or Kazahstan or Kosovo or "The Republic of Kosovo" or Kosova or Kocobo or Lebanon or Lubnan or Liban or Libya or "Tripolitania and Cyrenaica" or Libiya or Malaysia or Maldives or "Dhivehi Raajje" or "Marshall Islands" or "Aorokin Majel" or Mexico or Mexico or Mexihco or Montenegro or "Crna Gora" or Namibia or "South-West Africa" or Namibie or "North Macedonia" or "Severna Makedonija" or "Maqedonia e Veriut" or Paraguay or Paraguai or Peru or Peru or Piruw or Russia or "Russian Federation" or Rossiya or Rossia or Rossija or Samoa or Serbia or Srbija or "South Africa" or Suid-Afrika or "Sewula Afrika" or Afrika-Borwa or "Ningizimu Afrika" or "Aforika Borwa" or "Afrika Dzonga" or "Afurika Tshipembe" or "uMzantsi Afrika" or "Ningizimu Afrika" or "St. Lucia" or "Saint Lucia" or "St. Vincent and the Grenadines" or "Saint Vincent and the Grenadines" or Suriname or Thailand or Siam or "Prathet Thai" or Thai or "Ratcha-anachak Thai" or Tonga or Turkey or Turkiye or Turkmenistan or Tuvalu or "Venezuela, RB" or Venezuela).mp. [mp=title, book title, abstract, original title, name of substance word, subject heading word, floating sub-heading word, keyword heading word, organism supplementary concept word, protocol supplementary concept word, rare disease supplementary concept word, unique identifier, synonyms, population supplementary concept word, anatomy supplementary concept word] | 1952697 |
| 14 | 12 or 13 | 1952697 |
| 15 | 5 and 8 and 11 and 14 | 382 |
| 16 | limit 15 to (humans and yr="2021 -Current") | 95 |

**Search summary 2**

**EMBASE SEARCH STRATEGY**

1. exp Frontotemporal Dementia/ or "Mental Status and Dementia Tests"/ or dementia, multi-infarct/ or exp dementia/ or aids dementia complex/ or alzheimer disease/ or exp aphasia, primary progressive/ or creutzfeldt-jakob syndrome/ or exp dementia, vascular/ or diffuse neurofibrillary tangles with calcification/ or exp frontotemporal lobar degeneration/ or huntington disease/ or kluver-bucy syndrome/ or lewy body disease/ or "Pick Disease of the Brain"/ or Cerebral Amyloid Angiopathy/ or cognition disorders/ or cognitive dysfunction/

2. (senil* or "lewy bod*" or presenil* or alzheimer* or "cogniti* dis*" or "neurocogniti* dis*" or "cogniti* impair*" or "cogniti* defect*" or neurodegenerative or "delirium, dementia, amnestic, cognitive disorders" or "presenile dementia").m_titl.

3. 1 or 2

4. Caregivers/ or family/ or adult children/ or family relations/ or Friends/

5. ("famil* caregiver*" or caregiv* or famil* or relative* or "famil* care*" or "famil* support" or "famil* member*" or neighbo$r* or carer* or friend* or "informal caregiv*" or care* or caring or family or "adult children" or "child adult*" or "adult child*" or "sandwich carer*" or "sandwich caregiv*" or "family relations" or parent* or father* or spouse* or partner* or husband* or wife or wives or son* or daughter* or offspring* or sibling* or brother* or sister*).m_titl.

6. 4 or 5

7. Psychosocial Intervention/ or psychosocial support systems/ or internet-based intervention/ or social media/ or exp Cognitive Behavioral Therapy/ or social support/ or counseling/ or directive counseling/ or pastoral care/ or complementary therapies/ or holistic health/ or mind-body therapies/ or sensory art therapies/ or spiritual therapies/ or aromatherapy/ or art therapy/ or color therapy/ or dance therapy/ or music therapy/ or play therapy/

8. ("support* intervention*" or intervention* or therap* or engage* or therap* or program* or manage* or strateg* or polic* or support* or "non pharmacological" or "non-pharmacological" or "nonpharmacological" or "cognitive therapy" or "cognitive behavioural therapy" or CBT or "cognit* psychotherap*" or psychosocial or pyschoeducation* or psycho-education* or psycho* or social or "social support*" or befriend* or counsel* or counsel$ing or "directive counsel$ing" or "distance counsel$ing" or "alternative therap*" or "traditional therap*" or "doll therap*" or "pet* therap*" or "sensory therap*" or massage or "advance* care plan*" or "advance* plan*" or "decision* making aid*" or "decision* aid*" or "decision* support*" or "person* cent* care" or "patient* cent* care" or "dementia care" or "train*" or inform* or educat* or teach* or advis* or advice* or "education* intervention*" or resource* or "dementia training" or "dementia aware?" or "caregiver* education" or "caregiver* training" or "health education" or "peer-support" or "peer support network*" or "internet-based intervention*" or "social media" or pastoral or spiritual or religio* or "sensory art therap*" or "spiritual therap*" or "mind-body therap*" or "music therap*" or "art therap*" or aromatherap* or "dance therap*" or self-help or selfhelp).m_titl.

9. 7 or 8

10. Developing Countries/

11. (LMICS or "lower middle-income countries" or "Lower-MIC" or "low income countr*" or "low-income countr*" or "LICs" or "middle income countr*" or "upper middle-income countr*" or Upper-MIC or "developing countr*" or "developing nation*" or underdeveloped or under-developed or underserved or under-served or "emergent nation*" or "transitional countr*" or LAMI or "LAMI countr*" or "low and middle-income countr*" or "South Pacific" or "South Asia*" or "SE Asia*" or Angola or Algeria or Numidia or al-Jaza'ir or Bangladesh or Benin or Bhutan or "Druk Yul" or Bolivia or Buliwya or Wuliwya or Volivia or Cambodia or Kampuchea or Cameroon or Cameroun or Cameroon or "Cape Verde" or "Cabo Verde" or Comoros or Komori or "Juzur al-Qamar" or Comores or "Congo, Rep." or "Democratic Republic of Congo" or Zaire or "Republique du Congo" or "Repubilika ya Kongo" or "Republiki ya Kongo" or "Cote d'Ivoire" or "Ivory Coast" or Djibouti or Jibuti or Djibouti or Jabuuti or Gabuuti or "Egypt, Arab Rep." or Misr or Masr or "El Salvador" or Eswatini or Swaziland or eSwatini or Ghana or Gaana or Gana or Honduras or India or Bharot or Bharat or Bharata or Bharatam or Bharatadesam or Kenya or Kiribati or "Kyrgyz Republic" or Kyrgyzstan or Kirgizija or "Lao PDR" or Lao or Laos or Lesotho or Mauritania or Muritan or Agawec or Muritanya or "Micronesia, Fed. Sts. Tunisia" or "Federated States of Micronesia" or Moldova or Moldavia or Mongolia or "Mongol Uls" or Morocco or Amerruk or Elmeyrib or Al-mayreb or Myanmar or Burma or Myanma or Nepal or Nepal or Nicaragua or Nigeria or Nijeriya or Naijiriya or Naijiria or Pakistan or "Papua New Guinea" or "Papua Niugini" or "Papua Niu Gini" or Philippines or Pilipinas or "Sao Tome and Principe" or "Sao Tome e Principe" or Senegal or Senegaal or "Solomon Islands" or "Solomon Aelan" or "Sri Lanka" or Tanzania or "Tanganyika and Zanzibar, German East Africa" or Timor-Leste or "East Timor" or "Timor Lorosa'e" or Ukraine or Ukrajina or Uzbekistan or O'zbekiston or Vanuatu or Vietnam or "Viet Nam" or "South Vietnam" or "Cochin-China (south" or "Cochin-China" or "Annam (central)" or Annam or "Tonkin (north)" or Tonkin or "West Bank and Gaza" or "The State of Palestine" or Palestine or Zambia or "Northern Rhodesia" or Zimbabwe or "Southern Rhodesia" or "East Pakistan" or Rhodesia or Yugoslavia or Himalayan or Himalyan or Sahel or Yucatan or Palestine or Taiwan or Formosa or Palestinians or Rohingya or Michoacan* or Mixtec* or Zapotec* or Afghanistan or Afghanestan or "Burkina Faso" or "Upper Volta" or Burundi or Uburundi or "Central African Republic" or "Central Africa Republic, Chad" or Chad or "French Equatorial Africa" or Centrafrique or Beafrika or "Congo, Dem. Republic" or "Republique democratique du Congo" or "Republiki ya Kongo Demokratiki" or "Repubilika ya Kongo ya Dimokalasi" or "Jamhuri ya Kidemokrasia ya Kongo" or Eritrea or Iritriya or Ertra or Ethiopia or Abyssinia or Ityop'ia or "Gambia, The" or "The Gambia" or Guinea or Guinee or Gine or Guinea-Bissau or Guine-Bissau or Haiti or Ayiti or "Korea, Dem. People's Rep." or "Korea (North and South)" or Korea or Choson or Bukchoson or Liberia or Madagascar or Madagasikara or Malawi or Mali or "Sudanese Republic" or Mozambique or Mocambique or Niger or Rwanda or "Rwanda and Burundi" or "German East Africa" or "Sierra Leone" or Somalia or Soomaaliya or as-Sumal or "South Sudan" or "Sudan Kusini" or "Paguot Thudan" or Sudan or As-Sudan or "Syrian Arab Republic" or Syria or Suriyah or Tajikistan or Tojikistan or Togo or Uganda or "Yemen, Rep." or Al-Yaman or "Republic of Yemen").ti,ab,kw,cp.

12. 10 or 11

13. 3 and 6 and 9 and 12

14. limit 13 to human

15. limit 14 to embase

**Search summary 3**

**PsycINFO**

1. exp Frontotemporal Dementia/ or "Mental Status and Dementia Tests"/ or dementia, multi-infarct/ or exp dementia/ or aids dementia complex/ or alzheimer disease/ or exp aphasia, primary progressive/ or creutzfeldt-jakob syndrome/ or exp dementia, vascular/ or diffuse neurofibrillary tangles with calcification/ or exp frontotemporal lobar degeneration/ or huntington disease/ or kluver-bucy syndrome/ or lewy body disease/ or "Pick Disease of the Brain"/ or Cerebral Amyloid Angiopathy/ or cognition disorders/ or cognitive dysfunction/

2. (senil* or "lewy bod*" or presenil* or alzheimer* or "cogniti* dis*" or "neurocogniti* dis*" or "cogniti* impair*" or "cogniti* defect*" or neurodegenerative or "delirium, dementia, amnestic, cognitive disorders" or "presenile dementia").m_titl.

3. 1 or 2

4. Caregivers/ or family/ or adult children/ or family relations/ or Friends/

5. ("famil* caregiver*" or caregiv* or famil* or relative* or "famil* care*" or "famil* support" or "famil* member*" or neighbo$r* or carer* or friend* or "informal caregiv*" or care* or caring or family or "adult children" or "child adult*" or "adult child*" or "sandwich carer*" or "sandwich caregiv*" or "family relations" or parent* or father* or spouse* or partner* or husband* or wife or wives or son* or daughter* or offspring* or sibling* or brother* or sister*).m_titl.

6. 4 or 5

7. 3 and 6

8. Psychosocial Intervention/ or psychosocial support systems/ or internet-based intervention/ or social media/ or exp Cognitive Behavioral Therapy/ or social support/ or counseling/ or directive counseling/ or pastoral care/ or complementary therapies/ or holistic health/ or mind-body therapies/ or sensory art therapies/ or spiritual therapies/ or aromatherapy/ or art therapy/ or color therapy/ or dance therapy/ or music therapy/ or play therapy/

9. ("support* intervention*" or intervention* or therap* or engage* or therap* or program* or manage* or strateg* or polic* or support* or "non pharmacological" or "non-pharmacological" or "nonpharmacological" or "cognitive therapy" or "cognitive behavioural therapy" or CBT or "cognit* psychotherap*" or psychosocial or pyschoeducation* or psycho-education* or psycho* or social or "social support*" or befriend* or counsel* or counsel$ing or "directive counsel$ing" or "distance counsel$ing" or "alternative therap*" or "traditional therap*" or "doll therap*" or "pet* therap*" or "sensory therap*" or massage or "advance* care plan*" or "advance* plan*" or "decision* making aid*" or "decision* aid*" or "decision* support*" or "person* cent* care" or "patient* cent* care" or "dementia care" or "train*" or inform* or educat* or teach* or advis* or advice* or "education* intervention*" or resource* or "dementia training" or "dementia aware?" or "caregiver* education" or "caregiver* training" or "health education" or "peer-support" or "peer support network*" or "internet-based intervention*" or "social media" or pastoral or spiritual or religio* or "sensory art therap*" or "spiritual therap*" or "mind-body therap*" or "music therap*" or "art therap*" or aromatherap* or "dance therap*" or self-help or selfhelp).m_titl.

10. 8 or 9

11. 7 and 10

12. Developing Countries/

13. (LMICS or "lower middle-income countries" or "Lower-MIC" or "low income countr*" or "low-income countr*" or "LICs" or "middle income countr*" or "upper middle-income countr*" or Upper-MIC or "developing countr*" or "developing nation*" or underdeveloped or under-developed or underserved or under-served or "emergent nation*" or "transitional countr*" or LAMI or "LAMI countr*" or "low and middle-income countr*" or "South Pacific" or "South Asia*" or "SE Asia*" or Angola or Algeria or Numidia or al-Jaza'ir or Bangladesh or Benin or Bhutan or "Druk Yul" or Bolivia or Buliwya or Wuliwya or Volivia or Cambodia or Kampuchea or Cameroon or Cameroun or Cameroon or "Cape Verde" or "Cabo Verde" or Comoros or Komori or "Juzur al-Qamar" or Comores or "Congo, Rep." or "Democratic Republic of Congo" or Zaire or "Republique du Congo" or "Repubilika ya Kongo" or "Republiki ya Kongo" or "Cote d'Ivoire" or "Ivory Coast" or Djibouti or Jibuti or Djibouti or Jabuuti or Gabuuti or "Egypt, Arab Rep." or Misr or Masr or "El Salvador" or Eswatini or Swaziland or eSwatini or Ghana or Gaana or Gana or Honduras or India or Bharot or Bharat or Bharata or Bharatam or Bharatadesam or Kenya or Kiribati or "Kyrgyz Republic" or Kyrgyzstan or Kirgizija or "Lao PDR" or Lao or Laos or Lesotho or Mauritania or Muritan or Agawec or Muritanya or "Micronesia, Fed. Sts. Tunisia" or "Federated States of Micronesia" or Moldova or Moldavia or Mongolia or "Mongol Uls" or Morocco or Amerruk or Elmeyrib or Al-mayreb or Myanmar or Burma or Myanma or Nepal or Nepal or Nicaragua or Nigeria or Nijeriya or Naijiriya or Naijiria or Pakistan or "Papua New Guinea" or "Papua Niugini" or "Papua Niu Gini" or Philippines or Pilipinas or "Sao Tome and Principe" or "Sao Tome e Principe" or Senegal or Senegaal or "Solomon Islands" or "Solomon Aelan" or "Sri Lanka" or Tanzania or "Tanganyika and Zanzibar, German East Africa" or Timor-Leste or "East Timor" or "Timor Lorosa'e" or Ukraine or Ukrajina or Uzbekistan or O'zbekiston or Vanuatu or Vietnam or "Viet Nam" or "South Vietnam" or "Cochin-China (south" or "Cochin-China" or "Annam (central)" or Annam or "Tonkin (north)" or Tonkin or "West Bank and Gaza" or "The State of Palestine" or Palestine or Zambia or "Northern Rhodesia" or Zimbabwe or "Southern Rhodesia" or "East Pakistan" or Rhodesia or Yugoslavia or Himalayan or Himalyan or Sahel or Yucatan or Palestine or Taiwan or Formosa or Palestinians or Rohingya or Michoacan* or Mixtec* or Zapotec* or Afghanistan or Afghanestan or "Burkina Faso" or "Upper Volta" or Burundi or Uburundi or "Central African Republic" or "Central Africa Republic, Chad" or Chad or "French Equatorial Africa" or Centrafrique or Beafrika or "Congo, Dem. Republic" or "Republique democratique du Congo" or "Republiki ya Kongo Demokratiki" or "Repubilika ya Kongo ya Dimokalasi" or "Jamhuri ya Kidemokrasia ya Kongo" or Eritrea or Iritriya or Ertra or Ethiopia or Abyssinia or Ityop'ia or "Gambia, The" or "The Gambia" or Guinea or Guinee or Gine or Guinea-Bissau or Guine-Bissau or Haiti or Ayiti or "Korea, Dem. People's Rep." or "Korea (North and South)" or Korea or Choson or Bukchoson or Liberia or Madagascar or Madagasikara or Malawi or Mali or "Sudanese Republic" or Mozambique or Mocambique or Niger or Rwanda or "Rwanda and Burundi" or "German East Africa" or "Sierra Leone" or Somalia or Soomaaliya or as-Sumal or "South Sudan" or "Sudan Kusini" or "Paguot Thudan" or Sudan or As-Sudan or "Syrian Arab Republic" or Syria or Suriyah or Tajikistan or Tojikistan or Togo or Uganda or "Yemen, Rep." or Al-Yaman or "Republic of Yemen").ti,ab,lo,id.

14. 12 or 13

15. 11 and 14

16. limit 15 to human

**Search summary 4**

**PubMed**

PubMed Search: ("dementia"[Title] OR "frontotemporal dementia"[Title] OR ("mental status"[Title] OR (("dementia"[MeSH Terms] OR "dementia"[All Fields] OR "dementias"[All Fields] OR "dementia s"[All Fields]) AND "tests"[Title])) OR "multi infarct dementia"[Title] OR "aids dementia complex"[Title] OR "alzheimer disease"[Title] OR (("aphasia"[MeSH Terms] OR "aphasia"[All Fields] OR "aphasias"[All Fields] AND "aphasia s"[All Fields]) AND "primary progressive"[Title]) OR "creutzfeldt jakob syndrome"[Title] OR "vascular dementia"[Title] OR "diffuse neurofibrillary tangles with calcification"[Title] OR "frontotemporal lobar degeneration"[Title] OR "huntington disease"[Title] OR "kluver bucy syndrome"[Title] OR "lewy body disease"[Title] OR "cerebral amyloid angiopathy"[Title] OR "senile"[Title] OR "lewy bodies"[Title] OR "presenile"[Title] OR "alzheimer"[Title] OR "cognitive disorder"[Title] OR ("cognition disorders"[Title] OR "cognition disorder"[Title]) OR "cognitive disorders"[Title] OR "cognitive dysfunction"[Title] OR ("neurocognitive disorder"[Title] OR "neurocognitive disorders"[Title]) OR "cognitive impairment"[Title] OR ("cognition impairment"[Title] OR "cognition impairments"[Title]) OR ("cognitive defect"[Title] OR "cognitive defects"[Title]) OR "cognition defects"[Title] OR "neurodegenerative"[Title] OR ((("delirium"[MeSH Terms] OR "delirium"[All Fields] OR "deliriums"[All Fields]) OR ("dementia"[MeSH Terms] OR "dementia"[All Fields] OR "dementias"[All Fields] OR "dementia s"[All Fields]) OR ("amnestic"[All Fields] OR "amnestics"[All Fields])) OR "cognitive disorders"[Title]) OR "pick s dementia"[Title] OR (("pick disease of the brain"[MeSH Terms] OR ("pick"[All Fields] AND "disease"[All Fields] AND "brain"[All Fields]) OR "pick disease of the brain"[All Fields] OR ("pick"[All Fields] AND "disease"[All Fields]) OR "pick disease"[All Fields]) AND "of the brain"[Title]) OR "presenile dementia"[Title]) AND ("informal caregiver"[Title] OR "informal caregiver spouses"[Title] OR "informal caregivers"[Title] OR "informal caretakers"[Title] OR "informal caregiving"[Title] OR "informal carers"[Title] OR "informal carer"[Title] OR "informal caretaker"[Title] OR "family caregiver"[Title] OR "family caregiver s"[Title] OR "family caregivers"[Title] OR "family caregiving"[Title] OR "family carer"[Title] OR "family carers"[Title] OR "caregiver"[Title] OR "caregivers"[Title] OR "caregiving"[Title] OR "care giver"[Title] OR "care givers"[Title] OR "care giving"[Title] OR "carer"[Title] OR "carers"[Title] OR "caring"[Title] OR "family"[Title] OR "family members"[Title] OR "adult children"[Title] OR "adult children caregivers"[Title] OR "adult child"[Title] OR "adult child caregiver"[Title] OR "adult child caregivers"[Title] OR "adult child family caregivers"[Title] OR "adult child primary caregivers"[Title] OR "child adult"[Title] OR sandwich carer[Title] OR sandwich caregiver[Title] OR sandwich carers[Title] OR sandwich caregivers[Title] OR "family relations"[Title] OR "relative"[Title] OR "relatives"[Title] OR "family carer"[Title] OR "family carers"[Title] OR "family caregiver"[Title] OR "family caregiver s"[Title] OR "family caregivers"[Title] OR "family support"[Title] OR "neighbour"[Title] OR "neighbour s"[Title] OR "neighbours"[Title] OR "neighbor"[Title] OR "neighbors"[Title] OR "friend"[Title] OR "friend caregivers"[Title] OR "friends"[Title]) AND ((((((((((((((((((((((((((((((((((((((((((((((((((((((((((("supportive intervention"[Title] OR "supportive interventions"[Title]) OR ("supportive interventions"[Title])) OR ("support intervention"[Title])) OR ("support interventions"[Title])) OR ("psychosocial intervention"[Title] OR "psychosocial interventions"[Title])) OR ("internet based intervention"[Title] OR "internet based interventions"[Title])) OR ("social media"[Title])) OR ("intervention"[Title])) OR ("interventions"[Title])) OR ("therapy"[Title])) OR ("therapies"[Title])) OR ("program"[Title])) OR ("programs"[Title])) OR ("programme"[Title])) OR ("programmes"[Title])) OR ("management"[Title])) OR ("strategy"[Title])) OR ("strategies"[Title])) OR ("policy"[Title])) OR ("policies"[Title])) OR ("support"[Title])) OR ("supports"[Title])) OR ("supportive"[Title])) OR ("nonpharmacological"[Title])) OR ("non pharmacological"[Title])) OR ("non pharmacological"[Title])) OR ("cognitive therapy"[Title] OR "cognitive therapy group intervention"[Title])) OR ("cognitive behavioural therapy interventions"[Title])) OR ("cbt"[Title])) OR ("cognitive psychotherapy"[Title])) OR ("psychosocial support therapy"[Title] OR "psychosocial supportive"[Title] OR "psychosocial supportive care"[Title])) OR (psychosocial support systems[Title])) OR ("psychosocial intervention"[Title] OR "psychosocial interventions"[Title])) OR ("psychosocial"[Title])) OR ("psychoeducation"[Title] OR "psychoeducational"[Title])) OR ("psycho social"[Title])) OR ("social support"[Title])) OR ("befriend"[Title] OR "befriender"[Title] OR "befrienders"[Title] OR "befriending"[Title])) OR ("peer support"[Title] OR "peer support group"[Title] OR "peer support program"[Title] OR "peer support programme"[Title] OR "peer support programmes"[Title] OR "peer support programs"[Title])) OR ("peer support network"[Title] OR "peer support networks"[Title])) OR ("counseling"[Title])) OR ("counselling"[Title])) OR ("directive counseling"[Title] OR "directive counselling"[Title])) OR ("distance counseling"[Title] OR "distance counselling"[Title])) OR ("counsel"[Title])) OR ("therapy"[Title])) OR ("therapist"[Title])) OR ("therapists"[Title])) OR ("pastoral care"[Title])) OR ("pastoral"[Title])) OR ("spiritual"[Title])) OR ("religion"[Title])) OR ("religious"[Title])) OR ("complementary therapy"[Title])) OR ("complementary therapies"[Title]))) OR ("art therapies"[Title] OR "art therapists"[Title] OR "art therapy"[Title] OR "art therapist"[Title] OR "art therapy group"[Title])) OR ("spiritual therapy"[Title])) OR ("spiritual therapies"[Title])) AND ((((((((((((((((((((((((((((((((((((((((((((((((((((((((((((((((((((((((((((((((((((((((((((((((((((((((((((((((((((((((((((((((((((((((((((((((((((((((((((((((((((((((((((((((((((((((((((((((((((((((((((((((((((((((((((((((((((((((((((((((((((((((((((((((((((((((((((((((((((((((((((((((((((((((((((((((((((((((((((((((((((((((((((((((((((((((((((((((((((((((((((((((((("lmic"[All Fields] OR "lmic countries"[All Fields] OR "lmic country"[All Fields]) OR ("lmics"[All Fields] OR "lmics low"[All Fields] OR "lmics low and"[All Fields] OR "lmics low and middle"[All Fields] OR "lmics low and middle income"[All Fields] OR "lmics low and middle income countries"[All Fields])) OR ("lower middle income country"[All Fields] OR "lower middle income economies"[All Fields])) OR ("low and middle income countries"[All Fields])) OR ("lower middle income countries"[All Fields])) OR ("low and middle income"[All Fields] OR "low and middle income countries lmic"[All Fields])) OR (lower-MIC)) OR ("low income country"[All Fields] OR "low income developing countries"[All Fields])) OR ("low income countries"[All Fields])) OR ("lics"[All Fields])) OR ("middle income country"[All Fields] OR "middle income developing countries"[All Fields] OR "middle income developing country"[All Fields])) OR ("middle income developing country"[All Fields])) OR ("upper middle income country"[All Fields] OR "upper middle income economies"[All Fields])) OR ("upper middle income countries"[All Fields])) OR (upper MIC)) OR ("developing countries"[All Fields])) OR ("developing country"[All Fields])) OR ("developing nation"[All Fields] OR "developing nations"[All Fields])) OR ("underdeveloped"[All Fields] OR "underdeveloped countries"[All Fields] OR "underdeveloped country"[All Fields] OR "underdeveloped developing"[All Fields])) OR ("under developed"[All Fields] OR "under developed countries"[All Fields] OR "under developing countries"[All Fields])) OR ("underserved"[All Fields])) OR ("under served"[All Fields])) OR ("emergent nation"[All Fields] OR "emergent nations"[All Fields])) OR ("transitional countries"[All Fields] OR "transitional country"[All Fields])) OR ("lami"[All Fields] OR "lami countries"[All Fields] OR "lami country"[All Fields])) OR ("low middle income countries"[All Fields] OR "low middle income countries lmic"[All Fields] OR "low middle income countries lmics"[All Fields] OR "low middle income country"[All Fields] OR "low middle income country lmic"[All Fields])) OR ("south pacific"[All Fields])) OR ("south asia"[All Fields])) OR ("se asia"[All Fields])) OR ("angola"[All Fields])) OR ("algeria"[All Fields])) OR ("numidia"[All Fields])) OR (al-Jaza'ir)) OR ("bangladesh"[All Fields])) OR ("benin"[All Fields])) OR ("bhutan"[All Fields])) OR (druk yul)) OR ("bolivia"[All Fields])) OR (Buliwya)) OR (Wuliwya)) OR (Volivia)) OR ("cambodia"[All Fields])) OR ("kampuchea"[All Fields])) OR ("cameroon"[All Fields])) OR ("cameroun"[All Fields])) OR ("cape verde"[All Fields])) OR ("cabo verde"[All Fields])) OR ("comoros"[All Fields])) OR ("komori"[All Fields])) OR (Juzur al-Qamar)) OR ("comores"[All Fields])) OR (Congo, Rep.)) OR ("democratic republic of congo"[All Fields])) OR ("zaire"[All Fields])) OR ("republique du congo"[All Fields])) OR (Repubilika ya Kongo)) OR (Republiki ya Kongo)) OR ("cote d ivoire"[All Fields])) OR ("ivory coast"[All Fields])) OR ("djibouti"[All Fields])) OR ("jibuti"[All Fields])) OR ("djibouti"[All Fields])) OR (Jabuuti)) OR (Gabuuti)) OR (Egypt, Arab Rep.)) OR ("eqypt"[All Fields])) OR ("misr"[All Fields])) OR ("masr"[All Fields])) OR ("el salvador"[All Fields])) OR ("eswatini"[All Fields])) OR ("swaziland"[All Fields])) OR ("ghana"[All Fields])) OR (Gaana)) OR ("gana"[All Fields])) OR ("honduras"[All Fields])) OR ("india"[All Fields])) OR (Bharot)) OR ("bharat"[All Fields])) OR ("bharata"[All Fields])) OR ("bharatam"[All Fields])) OR (Bharatadesam)) OR ("kenya"[All Fields])) OR ("kiribati"[All Fields])) OR ("kyrgyz republic"[All Fields])) OR ("kyrgyzstan"[All Fields])) OR (Kirgizija)) OR ("lao pdr"[All Fields])) OR ("lao"[All Fields])) OR ("laos"[All Fields])) OR ("lesotho"[All Fields])) OR ("mauritania"[All Fields])) OR (Muritan)) OR (Agawec)) OR (Muritanya)) OR (Micronesia, Fed. Sts. Tunisia)) OR ("federated states of micronesia"[All Fields])) OR ("moldova"[All Fields])) OR ("moldavia"[All Fields])) OR ("mongolia"[All Fields])) OR (Mongol Uls)) OR ("morocco"[All Fields])) OR (Amerruk)) OR (Elmeyrib)) OR (Al-mayreb)) OR ("myanmar"[All Fields])) OR ("burma"[All Fields])) OR ("myanma"[All Fields])) OR ("nepal"[All Fields])) OR ("nicaragua"[All Fields])) OR ("nigeria"[All Fields])) OR (Nijeriya)) OR (Naijiriya)) OR (Naijiria)) OR ("pakistan"[All Fields])) OR ("papua new guinea"[All Fields])) OR ("papua niugini"[All Fields])) OR (Papua Niu Gini)) OR ("philippines"[All Fields])) OR ("pilipinas"[All Fields])) OR ("sao tome and principe"[All Fields])) OR ("sao tome e principe"[All Fields])) OR ("senegal"[All Fields])) OR (senegaal)) OR ("solomon islands"[All Fields])) OR (Solomon Aelan)) OR ("sri lanka"[All Fields])) OR ("tanzania"[All Fields])) OR (Tanganyika and Zanzibar, German East Africa)) OR (Timor-Leste)) OR ("east timor"[All Fields])) OR ("timor lorosa e"[All Fields])) OR ("ukraine"[All Fields])) OR (Ukrajina)) OR ("uzbekistan"[All Fields])) OR (O‘zbekiston)) OR ("vanuatu"[All Fields])) OR ("vietnam"[All Fields])) OR ("viet nam"[All Fields])) OR ("south vietnam"[All Fields])) OR (Cochin-China (south))) OR ("cochin china"[All Fields])) OR (Annam (central))) OR ("annam"[All Fields])) OR (Tonkin (north))) OR ("tonkin"[All Fields])) OR ("west bank and gaza"[All Fields])) OR (The State of Palestine)) OR ("palestine"[All Fields])) OR ("zambia"[All Fields])) OR ("northern rhodesia"[All Fields])) OR ("zimbabwe"[All Fields])) OR ("southern rhodesia"[All Fields])) OR ("east pakistan"[All Fields])) OR ("rhodesia"[All Fields])) OR ("yugoslavia"[All Fields])) OR ("himalayan"[All Fields])) OR ("himalyan"[All Fields])) OR ("sahel"[All Fields])) OR ("yucatan"[All Fields])) OR ("palestinians"[All Fields])) OR ("rohingya"[All Fields])) OR ("michoacan"[All Fields])) OR ("mixtec"[All Fields])) OR ("zapotec"[All Fields])) OR ("afghanistan"[All Fields])) OR ("afghanestan"[All Fields])) OR ("burkina faso"[All Fields])) OR ("upper volta"[All Fields])) OR ("burundi"[All Fields])) OR (Uburundi)) OR ("central african republic"[All Fields])) OR (Central Africa Republic, Chad)) OR ("chad"[All Fields])) OR ("french equatorial africa"[All Fields])) OR ("centrafrique"[All Fields])) OR (Beafrika)) OR (Congo, Dem. Republic)) OR ("republique democratique du congo"[All Fields])) OR (Republiki ya Kongo Demokratiki)) OR (Repubilika ya Kongo ya Dimokalasi)) OR (Jamhuri ya Kidemokrasia ya Kongo)) OR ("eritrea"[All Fields])) OR (Iritriya)) OR (Ertra)) OR ("ethiopia"[All Fields])) OR ("abyssinia"[All Fields])) OR (Ityop'ia)) OR (Gambia, The)) OR ("the gambia"[All Fields])) OR ("guinea"[All Fields])) OR ("guinee"[All Fields])) OR ("gine"[All Fields])) OR (Guinea-Bissau)) OR ("guinea bissau"[All Fields])) OR ("guine bissau"[All Fields])) OR ("timor leste"[All Fields])) OR (al mayreb)) OR (juzur al quamar)) OR (al jaza ir)) OR (lower MIC)) OR ("haiti"[All Fields])) OR ("ayiti"[All Fields])) OR (Korea, Dem. People's Rep.)) OR ("korea north"[All Fields])) OR ("north korea"[All Fields])) OR ("choson"[All Fields])) OR (Bukchoson)) OR ("liberia"[All Fields])) OR ("madagascar"[All Fields])) OR ("madagasikara"[All Fields])) OR ("malawi"[All Fields])) OR ("mali"[All Fields])) OR (Sudanese Republic)) OR ("mozambique"[All Fields])) OR ("mocambique"[All Fields])) OR ("niger"[All Fields])) OR ("rwanda"[All Fields])) OR (Rwanda and Burundi)) OR ("german east africa"[All Fields])) OR ("sierra leone"[All Fields])) OR ("somalia"[All Fields])) OR (Soomaaliya)) OR (as Sumal)) OR ("south sudan"[All Fields])) OR (Sudan Kusini)) OR (Paguot Thudan)) OR ("sudan"[All Fields])) OR (As Sudan)) OR ("syrian arab republic"[All Fields])) OR ("syria"[All Fields])) OR ("suriyah"[All Fields])) OR ("tajikistan"[All Fields])) OR (Tojikistan)) OR ("togo"[All Fields])) OR ("uganda"[All Fields])) OR (Yemen, Rep.)) OR ("yemen"[All Fields])) OR (Al-Yaman)) OR ("republic of yemen"[All Fields])) OR ("albania"[All Fields])) OR ("shqiperi"[All Fields])) OR ("shqiperia"[All Fields])) OR ("american samoa"[All Fields])) OR ("amerika samoa"[All Fields])) OR ("argentina"[All Fields])) OR ("armenia"[All Fields])) OR (Hayastan)) OR ("azerbaijan"[All Fields])) OR ("azerbaycan"[All Fields])) OR ("belarus"[All Fields])) OR ("bielarus"[All Fields])) OR ("belize"[All Fields])) OR ("bosnia and herzegovina"[All Fields])) OR ("bosna i hercegovina"[All Fields])) OR ("botswana"[All Fields])) OR ("brazil"[All Fields])) OR ("brasil"[All Fields])) OR ("bulgaria"[All Fields])) OR (Balgarija)) OR (Bulgariya)) OR ("china"[All Fields])) OR ("china north"[All Fields])) OR ("cathay"[All Fields])) OR ("china south"[All Fields])) OR ("mangi"[All Fields])) OR ("zhongguo"[All Fields])) OR ("colombia"[All Fields])) OR ("costa rica"[All Fields])) OR ("cuba"[All Fields])) OR ("dominica"[All Fields])) OR ("dominican republic"[All Fields])) OR ("republica dominicana"[All Fields])) OR ("ecuador"[All Fields])) OR ("equatorial guinea"[All Fields])) OR ("guinea ecuatorial"[All Fields])) OR ("guinee equatoriale"[All Fields])) OR (Guine Equatorial)) OR ("fiji"[All Fields])) OR ("viti"[All Fields])) OR ("gabon"[All Fields])) OR ("republique gabonaise"[All Fields])) OR ("georgia"[All Fields])) OR ("sakartvelo"[All Fields])) OR (Sak'art'velo)) OR ("grenada"[All Fields])) OR ("guatemala"[All Fields])) OR ("guyana"[All Fields])) OR ("indonesia"[All Fields])) OR (Iran, Islamic Rep.)) OR ("iran"[All Fields])) OR ("persia"[All Fields])) OR ("iraq"[All Fields])) OR ("mesopotamia"[All Fields])) OR (Al-'Iraq)) OR (Al Iraq)) OR ("eraq"[All Fields])) OR ("jamaica"[All Fields])) OR ("jordan"[All Fields])) OR (Al Urdun)) OR (Al-’Urdun)) OR ("kazakhstan"[All Fields])) OR (Qazaqstan))) OR ("kazahstan"[All Fields])) OR ("kosovo"[All Fields])) OR ("the republic of kosovo"[All Fields])) OR ("kosova"[All Fields])) OR (Кocobo)) OR ("lebanon"[All Fields])) OR ("lubnan"[All Fields])) OR ("liban"[All Fields])) OR ("libya"[All Fields])) OR (Tripolitania and Cyrenaica)) OR (Libiya)) OR ("malaysia"[All Fields])) OR ("maldives"[All Fields])) OR (Dhivehi Raajje)) OR ("marshall islands"[All Fields])) OR (Aorokin Majel)) OR ("mexico"[All Fields])) OR (Mexihco)) OR ("montenegro"[All Fields])) OR ("crna gora"[All Fields])) OR ("namibia"[All Fields])) OR ("south west africa"[All Fields])) OR ("namibie"[All Fields])) OR ("north macedonia"[All Fields])) OR (Severna Makedonija)) OR (Maqedonia e Veriut)) OR ("paraguay"[All Fields])) OR ("paraguai"[All Fields])) OR ("peru"[All Fields])) OR (Piruw)) OR ("russia"[All Fields])) OR ("russian federation"[All Fields])) OR ("rossiya"[All Fields])) OR ("rossia"[All Fields])) OR ("rossija"[All Fields])) OR ("samoa"[All Fields])) OR ("serbia"[All Fields])) OR ("srbija"[All Fields])) OR ("south africa"[All Fields])) OR ("suid afrika"[All Fields])) OR (Suid-Afrika)) OR (Sewula Afrika)) OR (Afrika Borwa)) OR (Afrika-Borwa)) OR (Ningizimu Afrika)) OR (Aforika Borwa)) OR (Afrika Dzonga)) OR (Afurika Tshipembe)) OR (uMzantsi Afrika)) OR (Ningizimu Afrika)) OR (St. Lucia)) OR ("saint lucia"[All Fields])) OR (St. Vincent and the Grenadines)) OR ("saint vincent and the grenadines"[All Fields])) OR ("suriname"[All Fields])) OR ("thailand"[All Fields])) OR ("siam"[All Fields])) OR (Prathet Thai)) OR ("thai"[All Fields])) OR (Ratcha anachak Thai)) OR (Ratcha-anachak Thai)) OR ("tonga"[All Fields])) OR ("turkey"[All Fields])) OR ("turkiye"[All Fields])) OR ("turkmenistan"[All Fields])) OR ("tuvalu"[All Fields])) OR (Venezuela, RB)) OR ("venezuela"[All Fields]))

**Supplementary Material 2: Meta-analysis of the effectiveness of non-pharmacological interventions, supplementary results**

**Supplementary result 2**

**NPI-Q Distress Scale meta-analysis results**

A total of six studies (361 participants)^44-49^ contributed data to caregiver distress using the NPI-Q Distress Scale. Meta-analysis indicated that NPIs in LMICs have a significant improvement on caregiver distress immediately or to first follow-up post-intervention (SMD 4·3, 95% CI 0·5 – 8·2; I^2^ = 66·5%); although there was substantial heterogeneity between studies there was consistency in direction of effects with five (83.3%) suggesting an improvement in the NPI-Q Distress Scale (Supplementary Figure 4). All studies were determined to be either at low risk or at some concern of bias.

**Supplementary Figure 4: Forest plot of NPI-Q main result**


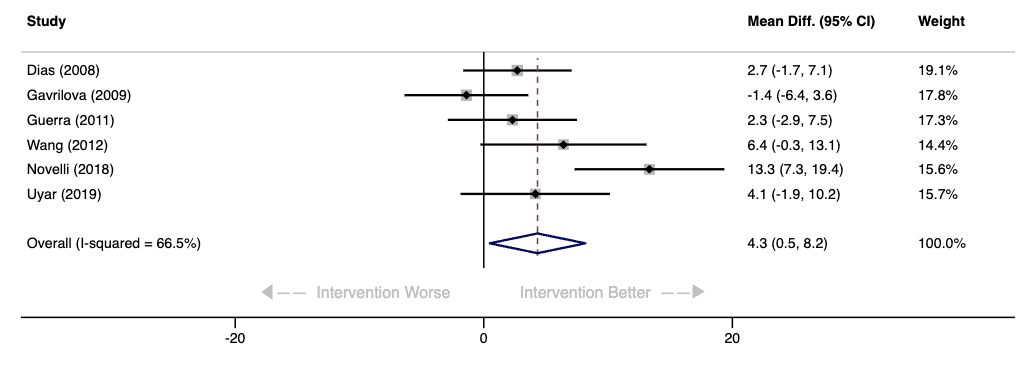


In total, five of these studies were delivered in person (SMD 4·4, 95% CI -0·2–9·0; I^2^ = 73·2%), and one a combination of in-person and remote (SMD 4·1, 95% CI -1·9–10.2; Supplementary Figure 5).

**Supplementary Figure 5: Forest plot of NPI-Q and intervention delivery modes**


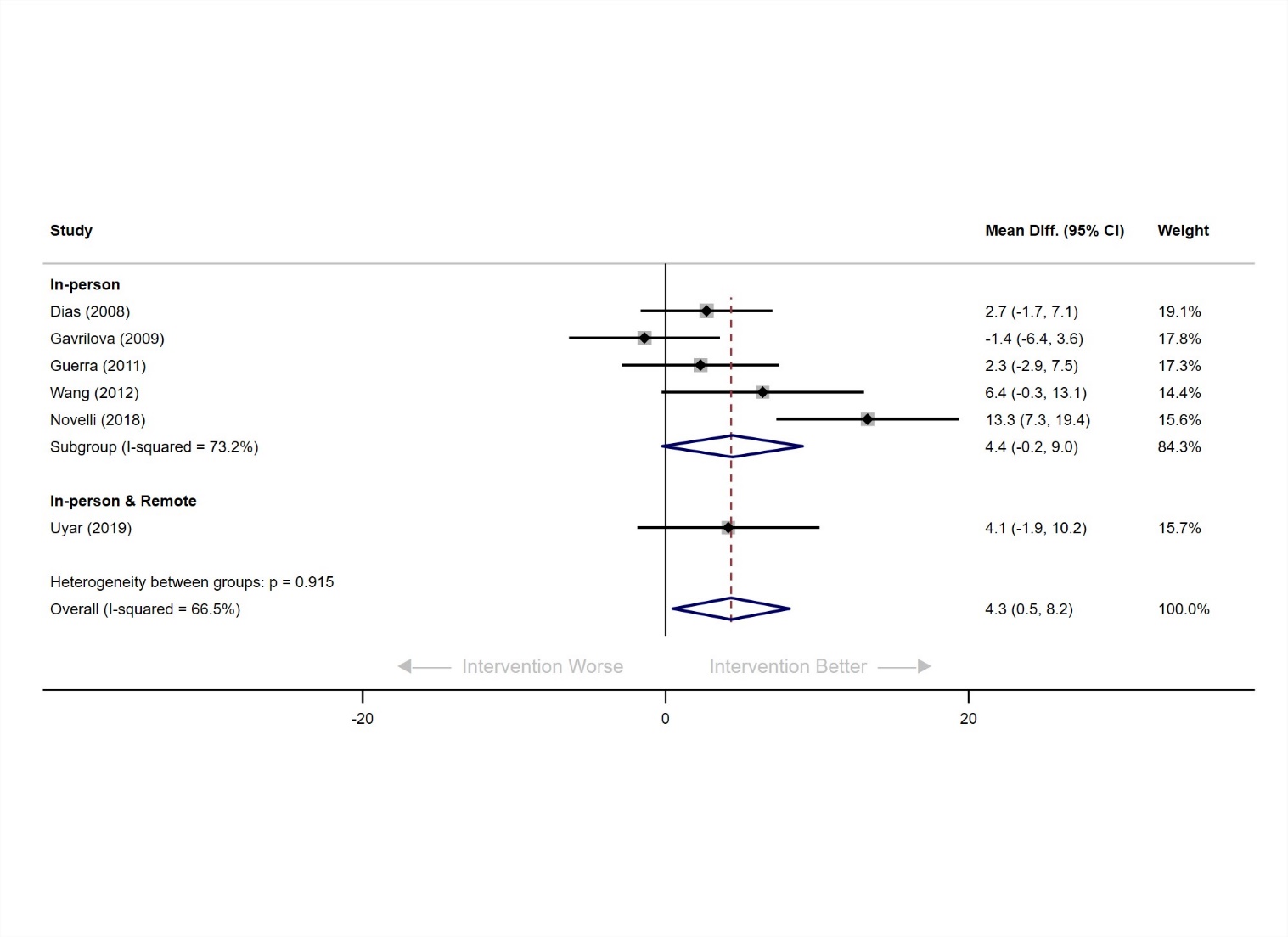


**Supplementary result 3**

**Beck Depression Inventory (BDI) meta-analysis results**

A total of four studies (214 participants)^47,50-52^ contributed data to address the effectiveness of NPIs on caregiver depression using the Beck Depression Inventory (BDI). Meta-analysis indicated that NPIs in LMICs have a significant improvement on caregiver depression immediately or to first follow-up post-intervention (SMD 5·9, 95% CI 1·7 – 10·1); although there was substantial heterogeneity between estimates (I^2^ = 67·0%) there was consistency in direction of effects with all suggesting an improvement in the BDI (Supplementary Figure 6). Overall, three studies were determined to be low risk or some concerns of bias (SMD 6·4, 95% CI 0·5 -12·4; I^2^ = 78·0%), and one study by Kamkhagi and colleagues,^51^ was considered to have high risk bias (SMD 4·9, 95% CI -0·2 -10·0).

**Supplementary Figure 6: Forest plot of BDI main results**
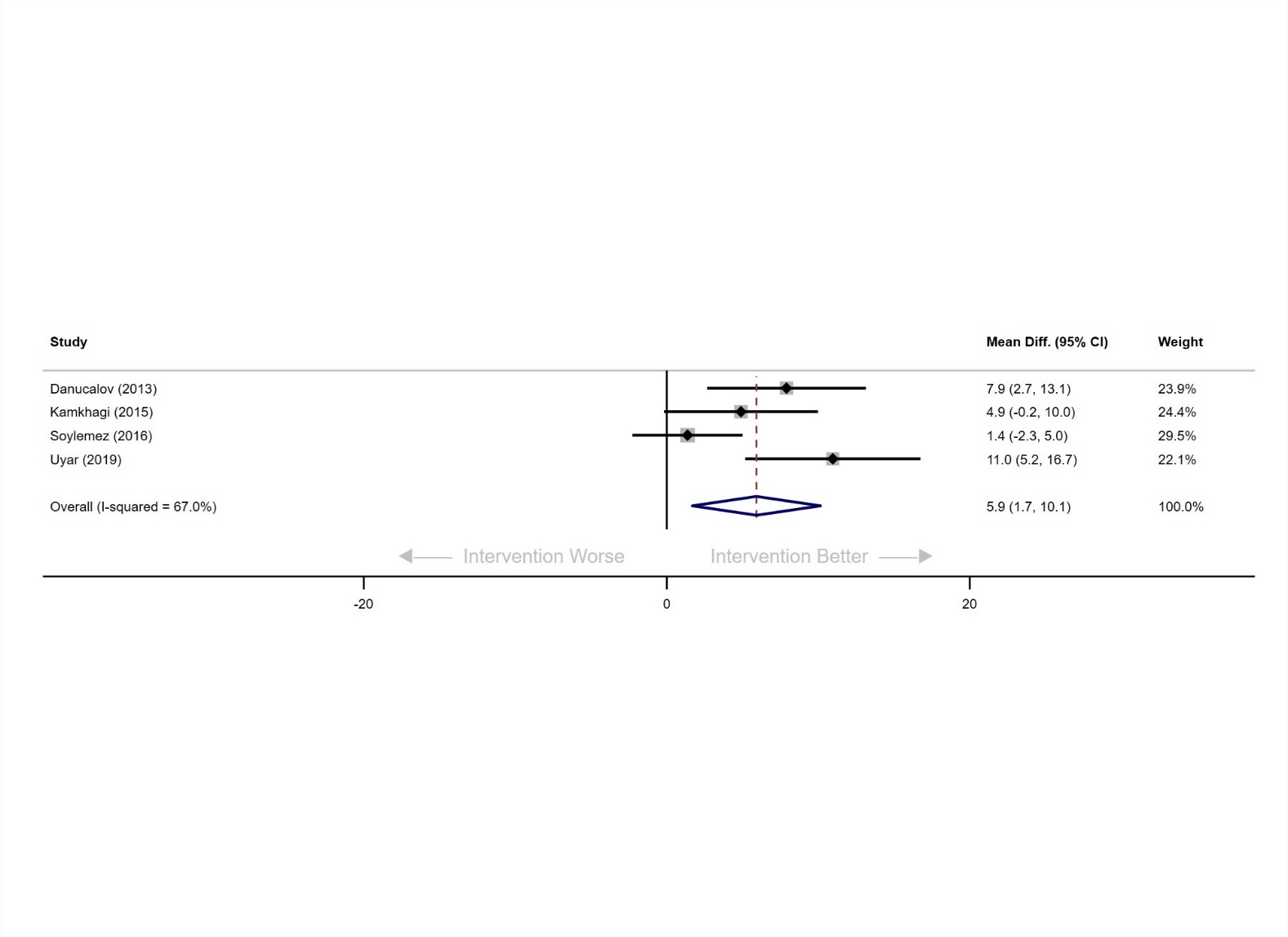


In total, three of these studies were delivered in a combination of in-person and remote (SMD 6·4, 95% CI 0·5 – 12·4; I^2^ = 78·0%), and the remaining study in-person (SMD 4·9, 95% CI -0·2–10·0). All demonstrated improvement in caregiver depression.

**Supplementary Figure 7: Forest plot of BDI and intervention delivery modes**


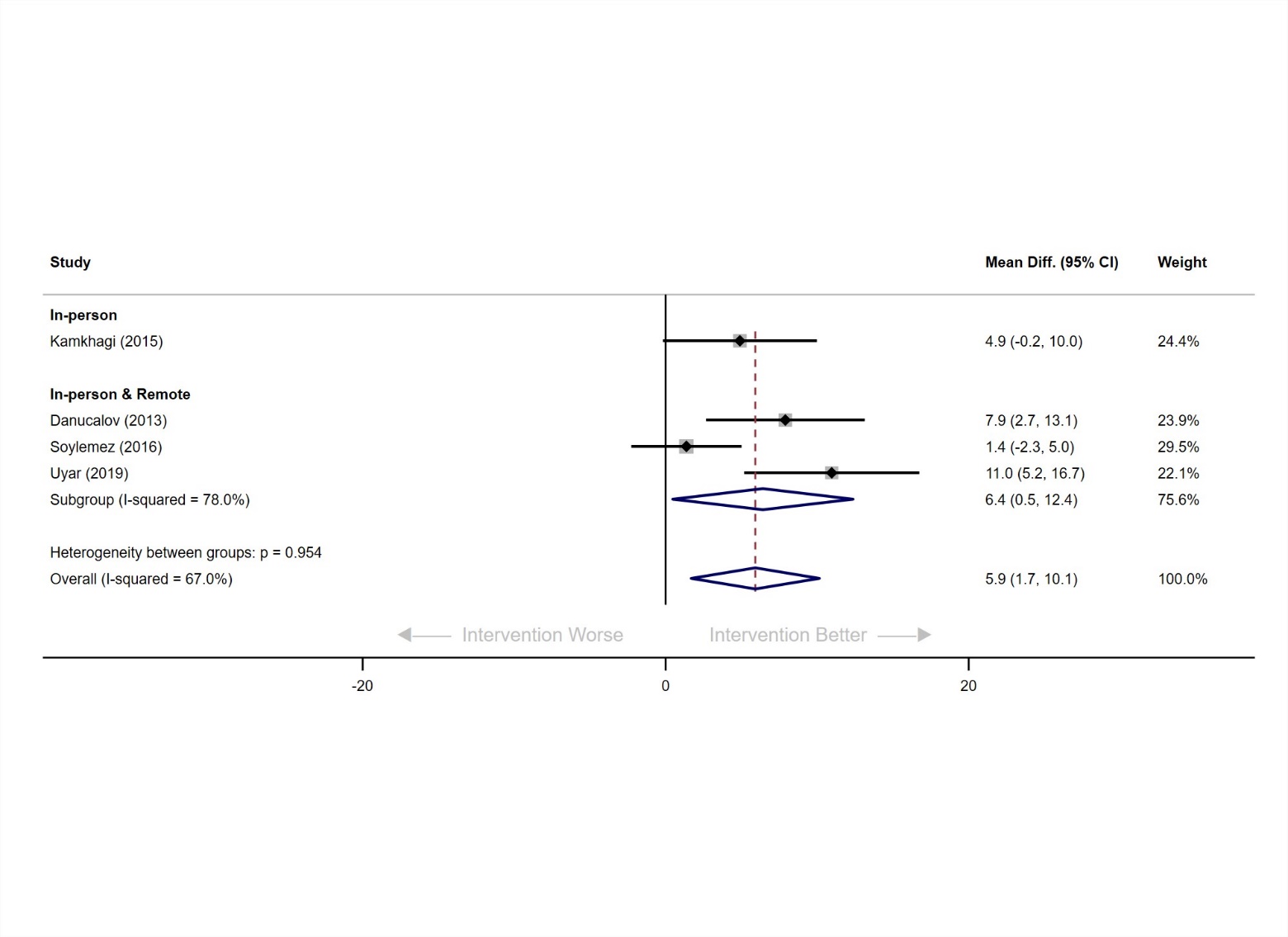


**Supplementary result 4**

**WHO Quality of Life BREF meta-analysis results**

A total of three studies (157 participants)^46,49,53^ contributed data to address the effectiveness of NPIs on caregiver wellbeing using the WHO Quality of Life BREF subdomains. Meta-analysis of results indicated there was no evidence that NPIs in LMICs have a significant improvement on the satisfaction of a caregiver to their surrounding environment (SMD 2·9, 95% CI -0·1 – 6·0; I^2^ = 17·5%), their physical health (SMD 2·0, 95% CI -0·8 – 4·8; I^2^ = 0·0%), their psychological health (SMD 2·2, 95% CI -0·4 – 4·7, I^2^ = 0·0%), and social health (SMD 0·8, 95% CI -3·6 – 5·3; I^2^ = 38·5%), immediately or to first follow-up post-intervention (Supplementary Figure 8). There was low heterogeneity between studies in each subdomain.

**Supplementary Figure 8: Forest plots of WHO-QOL Subdomain main results**

**Environmental**


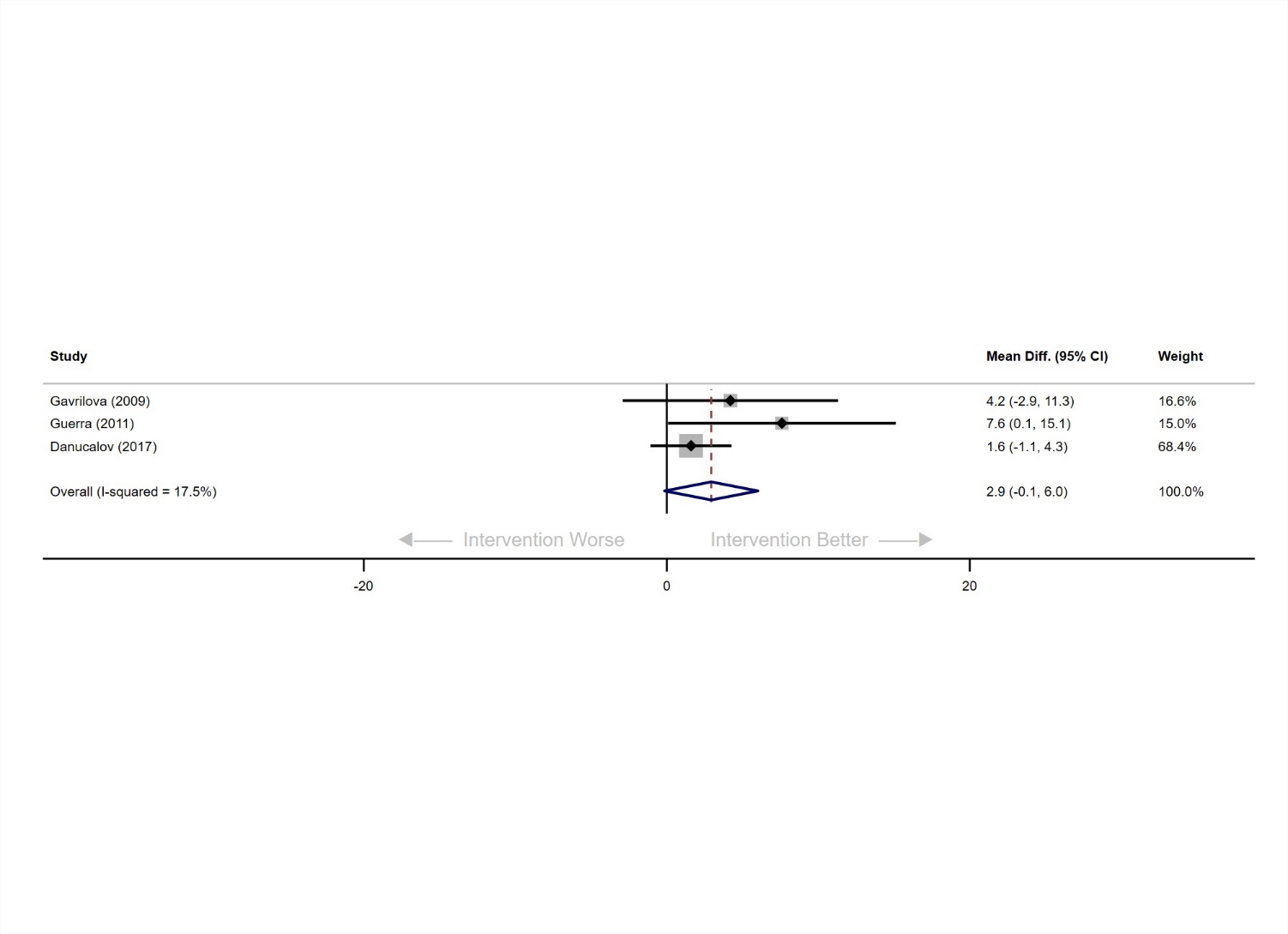


**Physical Health**


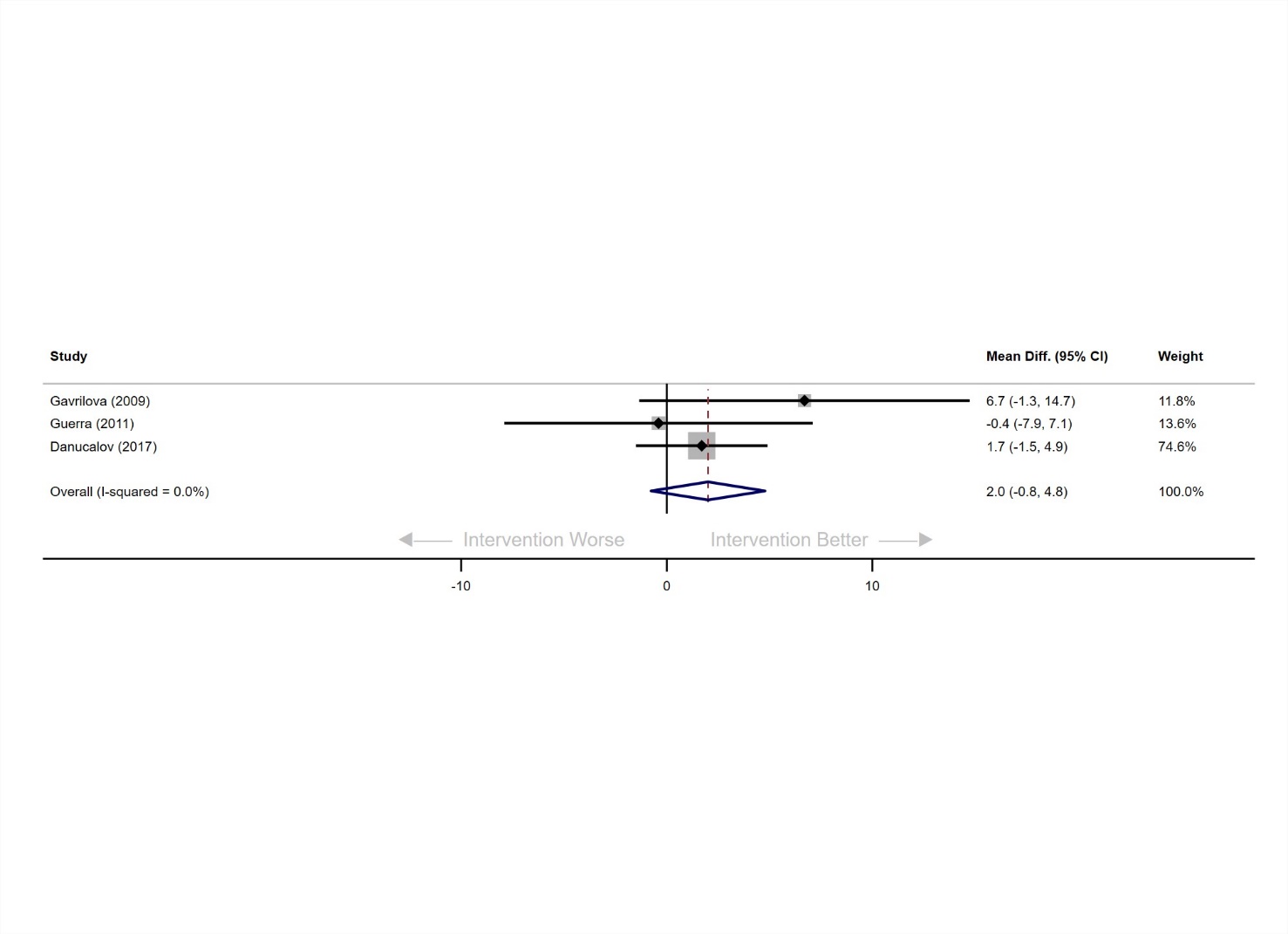


**Psychological Health**

**
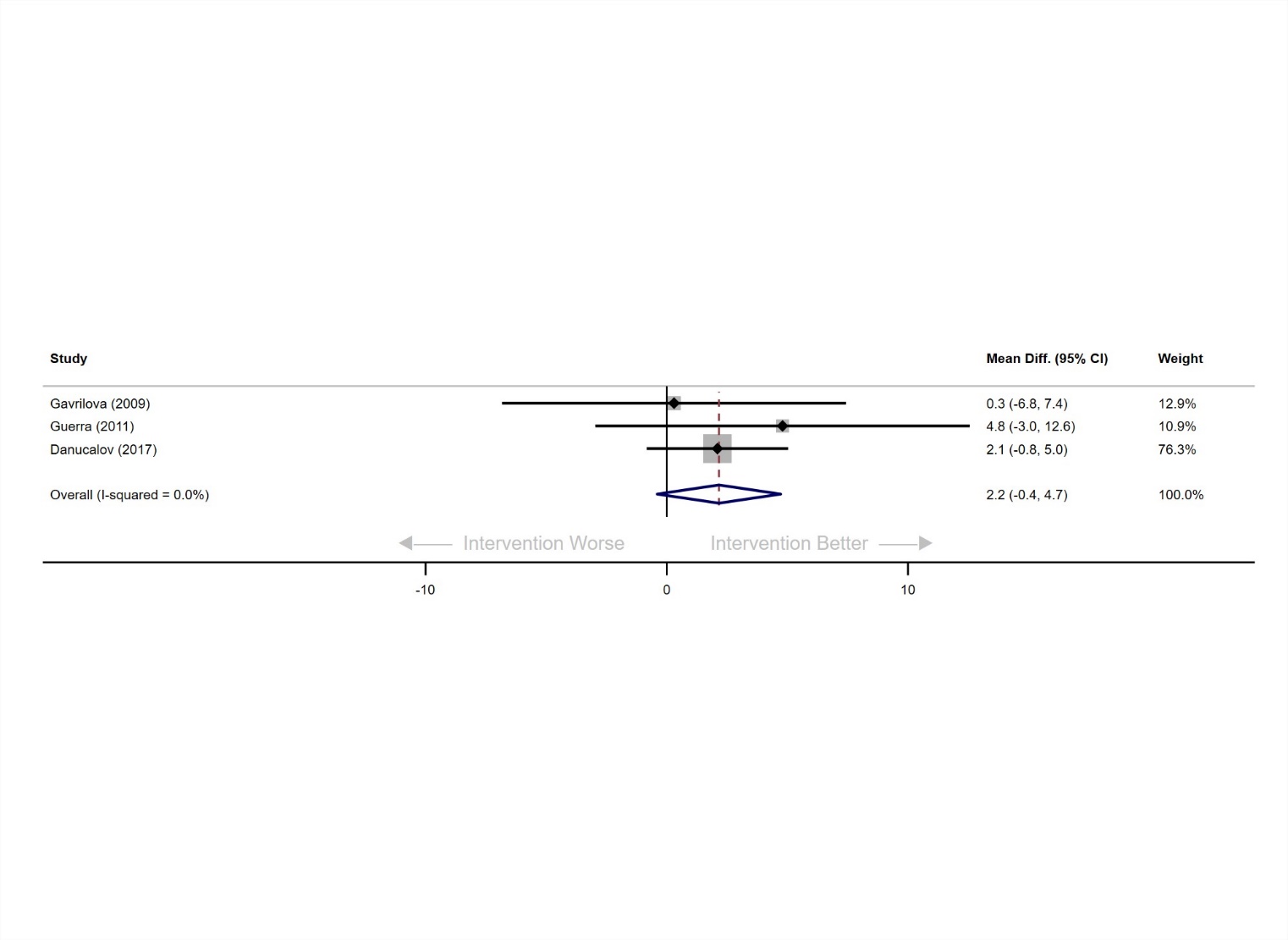
**

**Social Health**


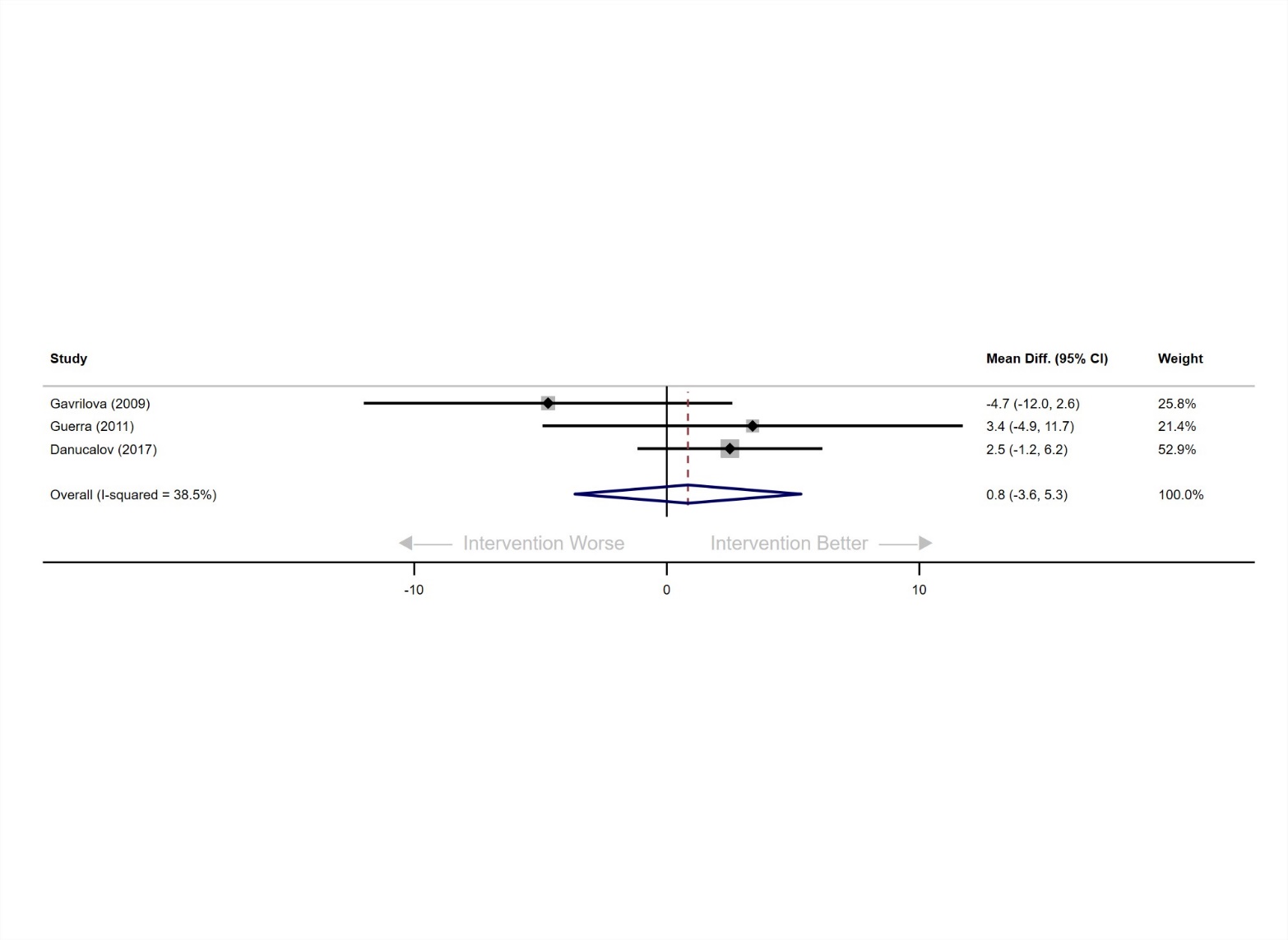


In total two of these studies were delivered in person and one in person and remote (Supplementary Figure 9).

**Supplementary Figure 9: Forest plot of WHO-QOL Domains and intervention delivery modes**

**Environmental**


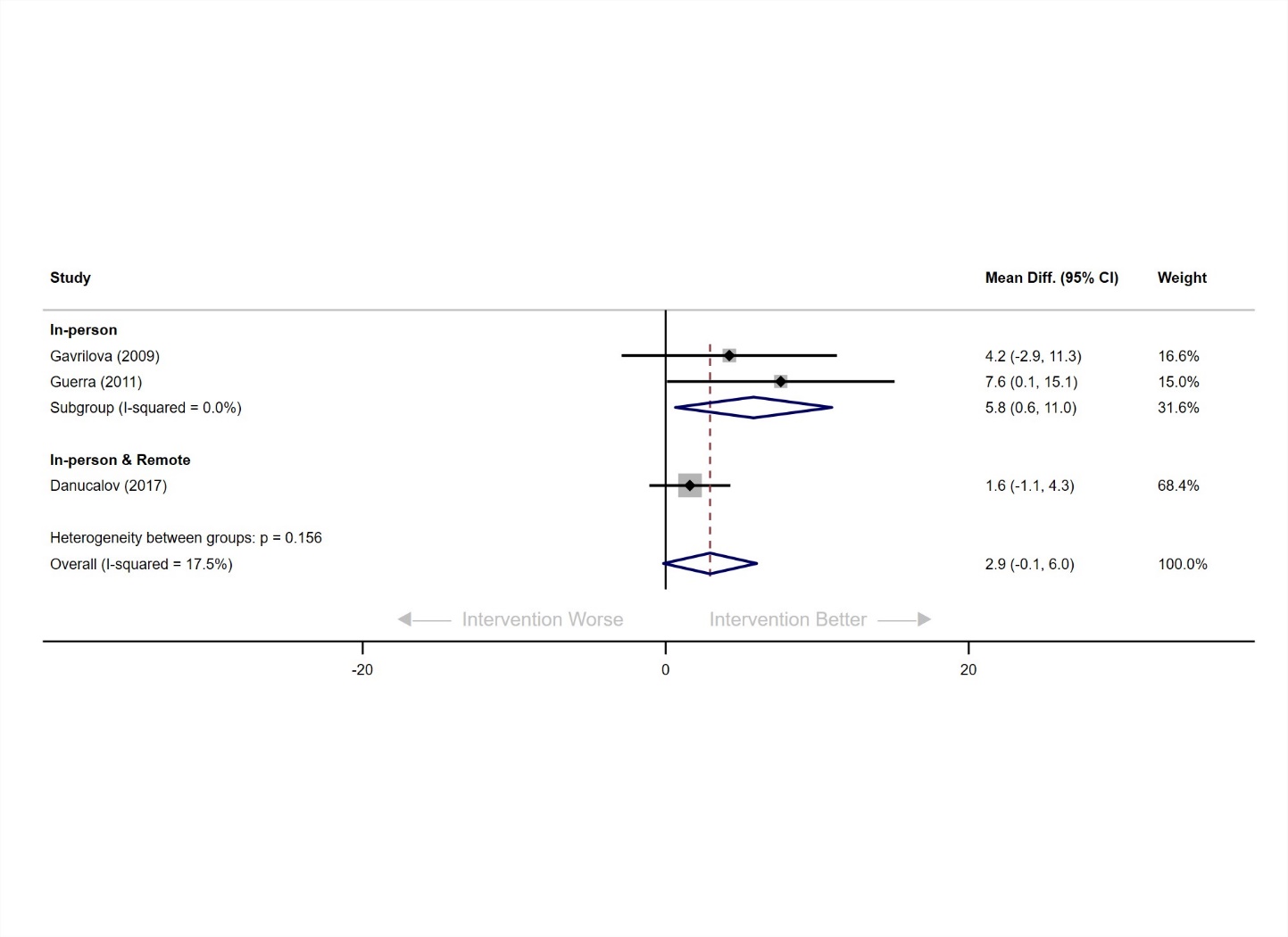


**Physical Health**


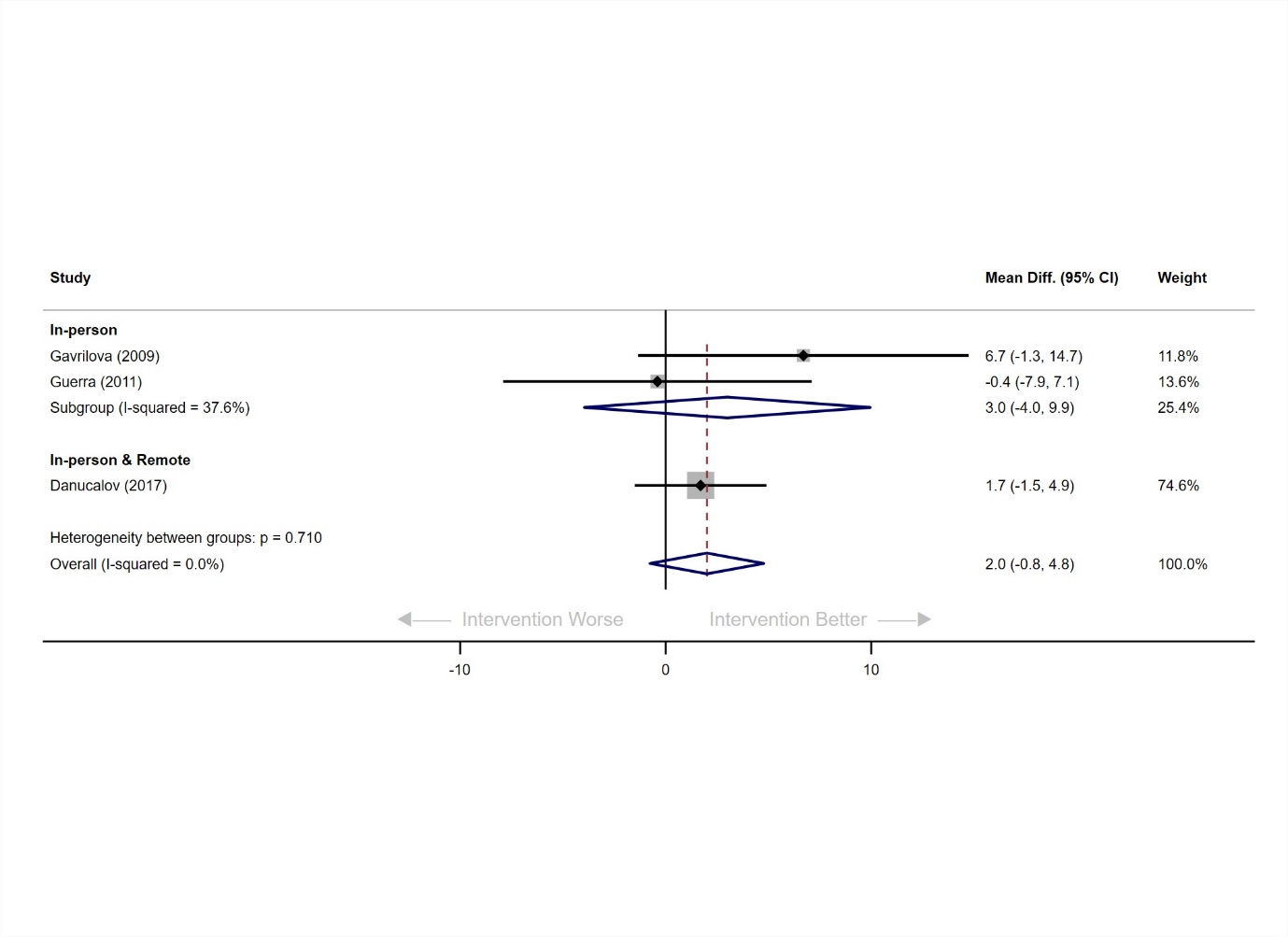


**Psychological Health**


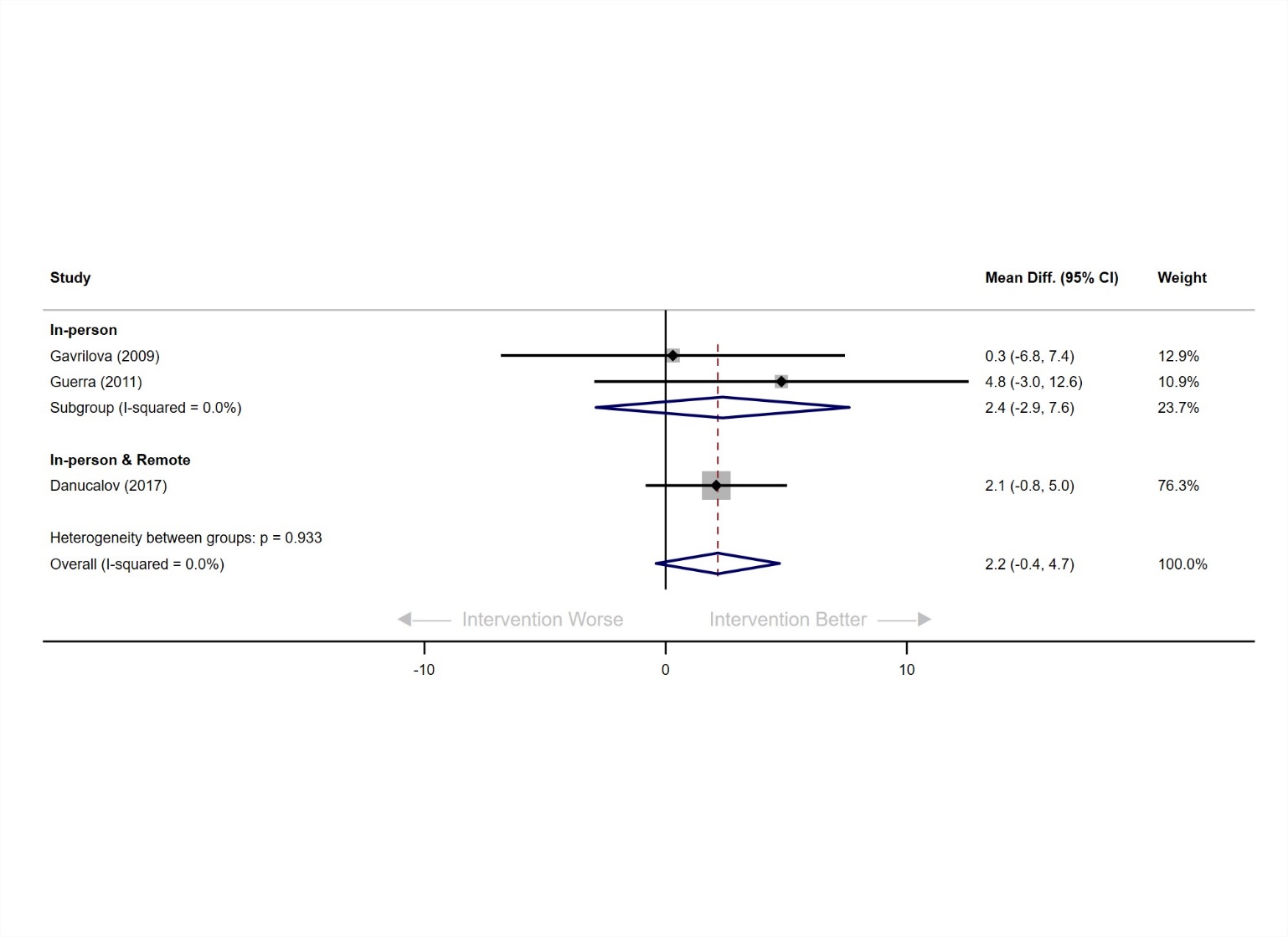


**Social Health**


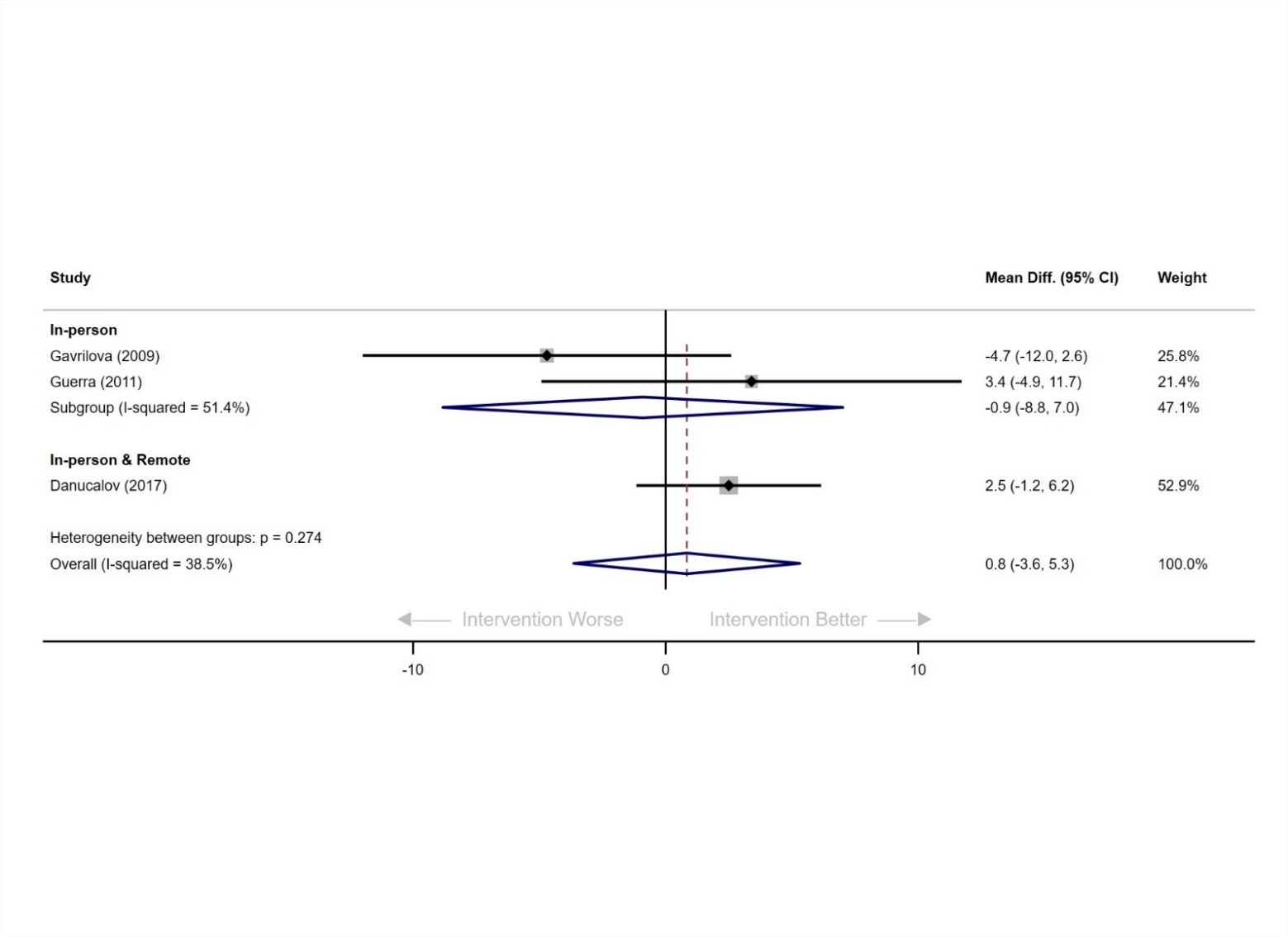


It was not possible to determine a meta-analysis of the WHO QOL Overall score as only two papers provided this data.^48,52^ The remaining authors were contacted but no additional data were accessible.

**Supplementary Material 3: Risk of Bias figures**

**Supplementary Figure 1: Risk of bias graph presented as percentages across all included parallel trials (n=27)**


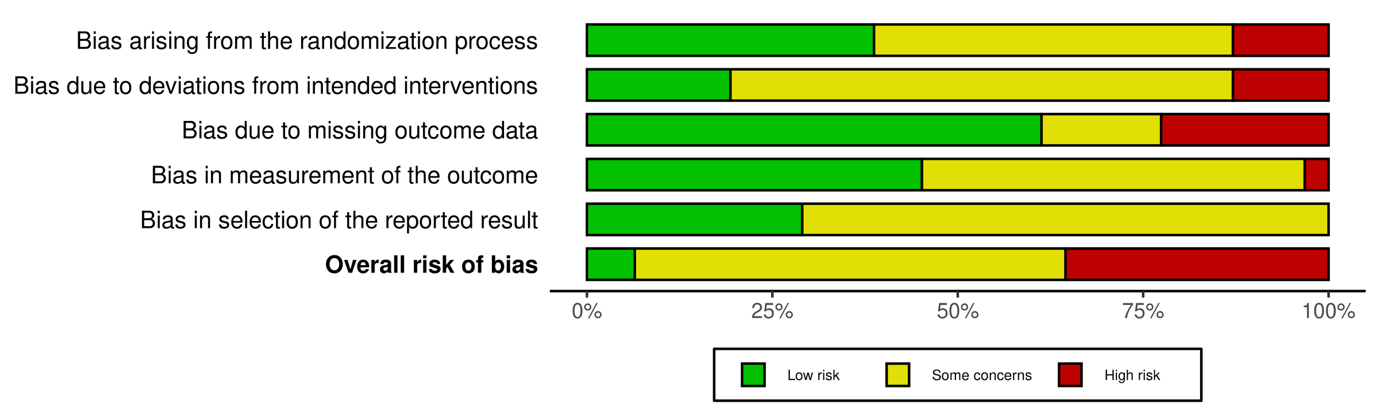


**Supplementary Figure 2: Risk of bias summary with domains for included parallel trials (n=31) and cluster RCT (n=2)**


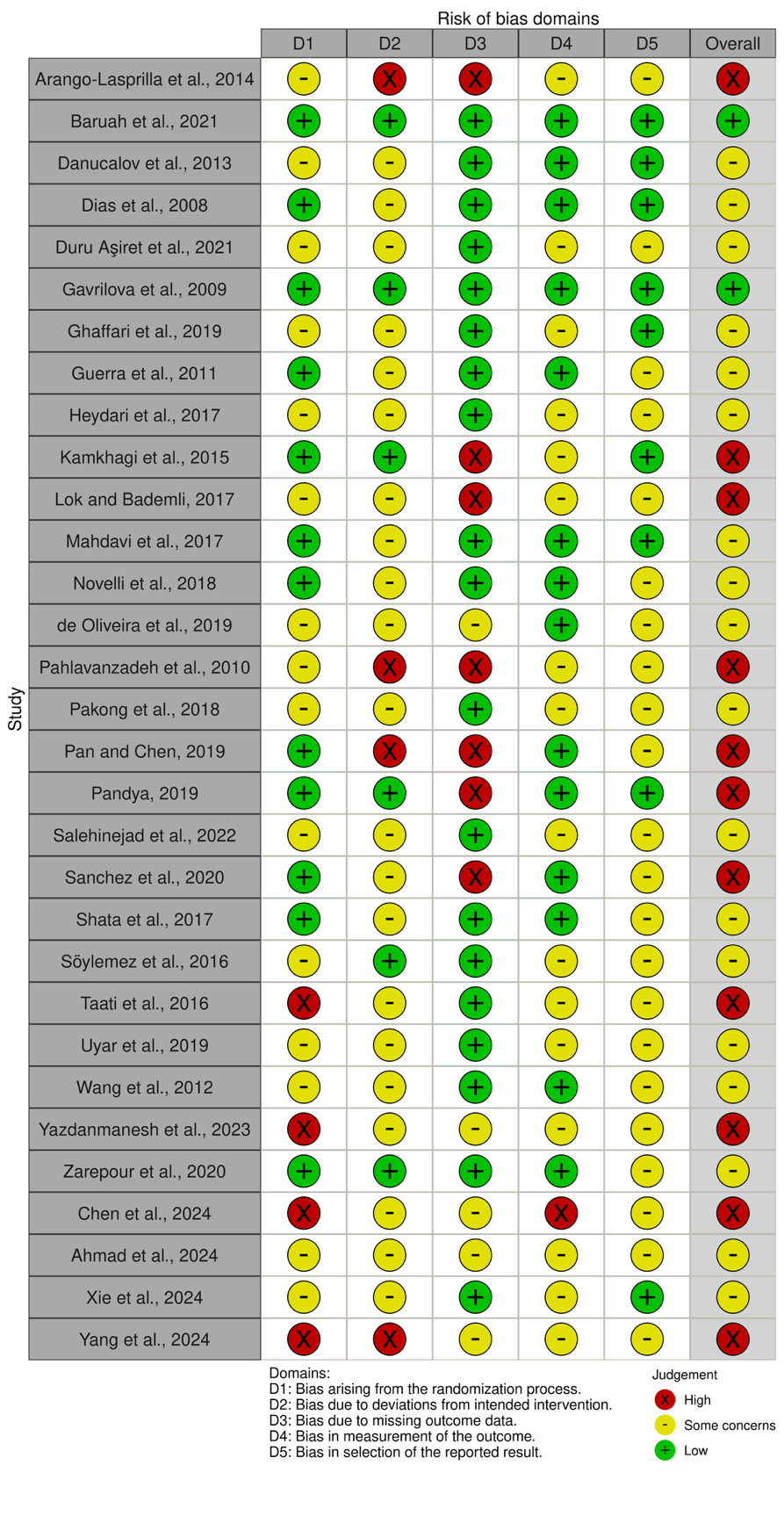


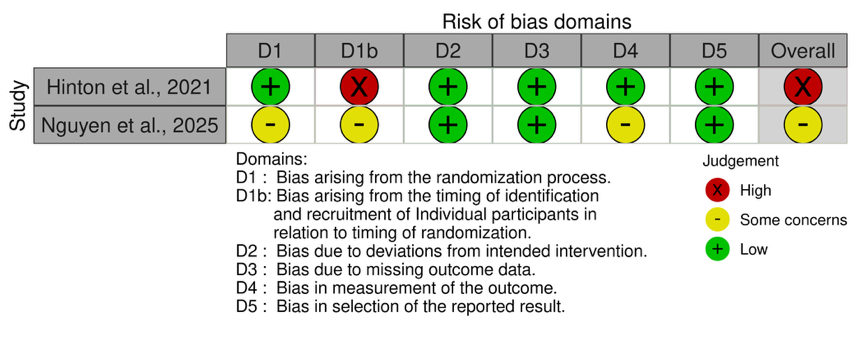


**Supplementary material 4: Relevant reviews screened n=137**

1. Abrahams R, Liu KPY, Bissett M, Fahey P, Cheung KSL, Bye R, et al. Effectiveness of interventions for co-residing family caregivers of people with dementia: Systematic review and meta-analysis. Australian Occupational Therapy Journal. 2018;65(3):208-24.

2. Acton GJ, Kang J. Interventions to reduce the burden of caregiving for an adult with dementia: a meta-analysis. Research in Nursing and Health. 2001;24(5):349-60.

3. Aravena JM, Gajardo J, Saguez R, Hinton L, Gitlin LN. Nonpharmacologic Interventions for Family Caregivers of People Living With Dementia in Latin-America: A Scoping Review. American Journal of Geriatric Psychiatry. 2022;30(8):859-77.

4. Boots LM, de Vugt ME, van Knippenberg RJ, Kempen GI, Verhey FR. A systematic review of Internet-based supportive interventions for caregivers of patients with dementia. International Journal of Geriatric Psychiatry. 2013;29(4):331-44.

5. Bourne P, Camic P, Crutch S. Psychosocial outcomes of dyadic arts interventions for people with a dementia and their informal caregivers: A systematic review. Health Soc Care Community. 2021;29:1632–49.

6. Brodaty H, Green A, Koschera A. Meta-analysis of psychosocial interventions for caregivers of people with dementia. Journal of the American Geriatrics Society. 2003;51(5):657-64.

7. Cheng ST, Zhang F. A comprehensive meta-review of systematic reviews and meta-analyses on nonpharmacological interventions for informal dementia caregivers. Bmc Geriatrics. 2020;20(1):24.

8. Chien LY, Chu H, Guo JL, Liao YM, Chang LI, Chen CH, et al. Caregiver support groups in patients with dementia: a meta-analysis. International Journal of Geriatric Psychiatry. 2011;26(10):1089-98.

9. Clarkson P, Hughes J, Roe B, Giebel CM, Jolley D, Poland F, et al. Systematic review: Effective home support in dementia care, components and impacts – Stage 2, effectiveness of home support interventions. J Adv Nurs. 2018;74(3):507-27.

10. Collins RN, Kishita N. The Effectiveness of Mindfulness- and Acceptance-Based Interventions for Informal Caregivers of People With Dementia: A Meta-Analysis. Gerontologist. 2019;59(4):e363-e79.

11. Cooper C, Balamurali TB, Selwood A, Livingston G. A systematic review of intervention studies about anxiety in caregivers of people with dementia. International Journal of Geriatric Psychiatry. 2007;22(3):181-8.

12. Corbett A, Stevens J, Aarsland D, Day S, Moniz-Cook E, Woods R, et al. Systematic review of services providing information and/or advice to people with dementia and/or their caregivers. International Journal of Geriatric Psychiatry. 2012;27(6):628-36.

13. Dam AEH, de Vugt ME, Klinkenberg IPM, Verhey FRJ, van Boxtel MPJ. A systematic review of social support interventions for caregivers of people with dementia: Are they doing what they promise? Maturitas. 2016;85:117-30.

14. de Oliveira Lopes L, Cachioni M. [Psychoeducational intervention for caregivers of elderly with dementia: a systematic review]. Jornal Brasileiro de Psiquiatria. 2012;61(4):252-61.

15. Deeken F, Rezo A, Hinz M, Discher R, Rapp MA. Evaluation of Technology-Based Interventions for Informal Caregivers of Patients With Dementia—A Meta-Analysis of Randomized Controlled Trials. The American Journal of Geriatric Psychiatry. 2019;27(4):426-45.

16. Egan KJ, Pinto-Bruno ÁC, Bighelli I, Berg-Weger M, van Straten A, Albanese E, et al. Online Training and Support Programs Designed to Improve Mental Health and Reduce Burden Among Caregivers of People With Dementia: A Systematic Review. Journal of the American Medical Directors Association. 2018;19(3):200-6.e1.

17. Elvish R, Lever SJ, Johnstone J, Cawley R, Keady J. Psychological interventions for carers of people with dementia: a systematic review of quantitative and qualitative evidence. British Association for Counselling & Psychotherapy. 2012:01-32.

18. Gallagher-Thompson D, Coon DW. Evidence-based psychological treatments for distress in family caregivers of older adults. Psychol Aging. 2007;22(1):37-51.

19. Godwin KM, Mills WL, Anderson JA, Kunik ME. Technology-driven interventions for caregivers of persons with dementia: a systematic review. American Journal of Alzheimer's Disease and Other Dementias. 2013;28(3):216-22.

20. Gonzalez FJ, Gaona C, Quintero M, Chavez CA, Selga J, Maestre GE. Building capacity for dementia care inLatin America and the Caribbean. Dement neuropsychol. 2014;8(4).

21. Henry Brodaty, D.Sc. , and, Caroline Arasaratnam, B.Psych. Meta-Analysis of Nonpharmacological Interventions for Neuropsychiatric Symptoms of Dementia. American Journal of Psychiatry. 2012;169(9):946-53.

22. Hinton L, Tran D, Nguyen T-N, Ho J, Gitlin L. Interventions to support family caregivers of people living with dementia in high, middle and low-income countries in Asia: a scoping review. 2019.

23. Hopkinson MD, Reavell J, Lane DA, Mallikarjun P. Cognitive Behavioral Therapy for Depression, Anxiety, and Stress in Caregivers of Dementia Patients: A Systematic Review and Meta-Analysis. The Gerontologist. 2018;59(4):e343-e62.

24. Hurley RV, Patterson TG, Cooley SJ. Meditation-based interventions for family caregivers of people with dementia: a review of the empirical literature. Aging and Mental Health. 2014;18(3):281-8.

25. Jensen M, Agbata IN, Canavan M, McCarthy G. Effectiveness of educational interventions for informal caregivers of individuals with dementia residing in the community: Systematic review and meta-analysis of randomised controlled trials. International Journal of Geriatric Psychiatry. 2015;30(2):130-43.

26. Kaddour L, Kishita N, Schaller A. A meta-analysis of low-intensity cognitive behavioral therapy-based interventions for dementia caregivers. International Psychogeriatrics. 2019;31(7):961-76.

27. Kishita N, Hammond L, Dietrich CM, Mioshi E. Which interventions work for dementia family carers?: an updated systematic review of randomized controlled trials of carer interventions. International Psychogeriatrics. 2018;30(11):1679-96.

28. Kor PPK, Chien WT, Liu JYW, Lai CKY. Mindfulness-Based Intervention for Stress Reduction of Family Caregivers of People with Dementia: A Systematic Review and Meta-Analysis. Mindfulness. 2018;9(1):7-22.

29. Lamotte G, Shah RC, Lazarov O, Corcos DM. Exercise Training for Persons with Alzheimer's Disease and Caregivers: A Review of Dyadic Exercise Interventions. J Mot Behav. 2017;49(4):365-77.

30. Lasrado R, Baker S, Zubair M, Kaiser P, Lasrado VJ, Rizzo M, et al. Exploring Dementia Care Systems Across the African Caribbean Diaspora: A Scoping Review and Consultation Exercise. Gerontologist. 2021;61(5):e209-e27.

31. Laver K, Milte R, Dyer S, Crotty M. A Systematic Review and Meta-Analysis Comparing Carer Focused and Dyadic Multicomponent Interventions for Carers of People With Dementia. Journal of aging and health. 2017;29(8):1308-49.

32. Leng M, Zhao Y, Xiao H, Li C, Wang Z. Internet-Based Supportive Interventions for Family Caregivers of People With Dementia: Systematic Review and Meta-Analysis. J Med Internet Res. 2020;22(9):e19468.

33. Li R, Cooper C, Austin A, Livingston G. Do changes in coping style explain the effectiveness of interventions for psychological morbidity in family carers of people with dementia? A systematic review and meta-analysis. International Psychogeriatrics. 2013;25(2):204-14.

34. Lins S, Hayder-Beichel D, Rücker G, Motschall E, Antes G, Meyer G, et al. Efficacy and experiences of telephone counselling for informal carers of people with dementia. Cochrane Database of Systematic Reviews: Reviews. 2014;Issue 9.

35. Liu Z, Chen Q-L, Sun Y-Y. Mindfulness training for psychological stress in family caregivers of persons with dementia: a systematic review and meta-analysis of randomized controlled trials. Clin Interv Aging. 2017;12:1521-9.

36. Ma KPK, Saw A. An international systematic review of dementia caregiving interventions for Chinese families. Int J Geriatr Psychiatry. 2020;35(11):1263-84.

37. Marim CM, Silva V, Taminato M, Barbosa DA. Effectiveness of educational programs on reducing the burden of caregivers of elderly individuals with dementia: a systematic review. Revista Latino-Americana de Enfermagem. 2013;21(Special Issue):267-75.

38. McKechnie V, Barker C, Stott J. Effectiveness of computer-mediated interventions for informal carers of people with dementia – a systematic review. International Psychogeriatrics. 2014;26(10):1619-37.

39. Morris L, Horne M, McEvoy P, Williamson T. Communication training interventions for family and professional carers of people living with dementia: a systematic review of effectiveness, acceptability and conceptual basis. Aging & Mental Health. 2018;22(7):863-80.

40. Nelis S, Quinn C, Clare L. Information and support interventions for informal caregivers of people with dementia. Cochrane Database of Systematic Reviews: Reviews. 2007;Issue 2.

41. Nguyen H, Terry D, Phan H, Vickers J, McInerney F. Communication training and its effects on carer and care-receiver outcomes in dementia settings: A systematic review. J Clin Nurs. 2019;28(7-8):1050-69.

42. Nuraisyah Hani Z, Suriani I, Rosliza Abdul M, Lim Poh Y. Educational Intervention for Informal Caregiver of Person with Dementia: A Systematic Review. Malaysian Journal of Medicine and Health Sciences. 2020:325-31.

43. O'Connor CM, Clemson L, Silva TBLd, Piguet O, Hodges JR, Mioshi E. Enhancement of carer skills and patient function in the non-pharmacological management of frontotemporal dementia - FTD: a call for randomised controlled studies. Dement neuropsychol. 2013;7(2).

44. Ogunniyi A, Hall KS, Baiyewu O, Gureje O, Unverzagt FW, Gao S, et al. Caring for individuals with dementia. The Nigerian experience. West African Journal of Medicine. 2005;24(3):259-62.

45. Parker D, Mills S, Abbey J. Effectiveness of interventions that assist caregivers to support people with dementia living in the community: a systematic review. Int J Evid Based Healthc. 2008;6(2):137-72.

46. Peacock SC, Forbes DA. Interventions for caregivers of persons with dementia: a systematic review. Canadian Journal of Nursing Research. 2003;35(4):88-107.

47. Pinquart M, Sorensen S. Helping caregivers of persons with dementia: which interventions work and how large are their effects? International Psychogeriatrics. 2006;18(4):577-95.

48. Prince MJ, Acosta D, Castro-Costa E, Jackson J, Shaji KS. Packages of Care for Dementia in Low- and MiddleIncome Countries

<Eldis Update 28 January 2023 "dementia" (n=33).pdf>. PLoS Medicine / Public Library of Science. 2009;6(11).

49. Pusey H, Richards D. A systematic review of the effectiveness of psychosocial interventions for carers of people with dementia. Aging and Mental Health. 2001;5(2):107-19.

50. Qiu D, Hu M, Yu Y, Tang B, Xiao S. Acceptability of psychosocial interventions for dementia caregivers: a systematic review. BMC Psychiatry. 2019;19(1):23.

51. Rathnayake S, Moyle W, Jones C, Calleja P. mHealth applications as an educational and supportive resource for family carers of people with dementia: An integrative review. Dementia. 2019;18(7-8):3091-112.

52. Reis Ed, Novelli MMPC, Guerra RLF. Intervenções realizadas com grupos de cuidadores de idosos com síndrome demencial: revisão sistemática. Cad Bras Ter Ocup. 2018;26(3):646-57.

53. Resciniti NV, Tang W, Tabassum M, Pearson JL, Spencer SM, Lohman MC, et al. Knowledge evaluation instruments for dementia caregiver education programs: A scoping review. Geriatrics & Gerontology International. 2020;20(5):397-413.

54. Schoenmakers B, Buntinx F, DeLepeleire J. Supporting the dementia family caregiver: the effect of home care intervention on general well-being. Aging and Mental Health. 2010;14(1):44-56.

55. Selwood A, Johnston K, Katona C, Lyketsos C, Livingston G. Systematic review of the effect of psychological interventions on family caregivers of people with dementia. Journal of Affective Disorders. 2007;101(1-3):75-89.

56. Seyeon P, Myonghwa P. Effects of Family Support Programs for Caregivers of People with Dementia - Caregiving Burden, Depression, and Stress: Systematic Review and Meta-analysis. Journal Korean acad. 2015:627-40.

57. Shi H, Mao C, Tang J, Liang H. Research on the health of and interventions for family caregivers of people with dementia: a bibliometric analysis of research output during 1988-2018. BMC Geriatrics. 2020;20(1):1-9.

58. Smits CH, de Lange J, Droes RM, Meiland F, Vernooij-Dassen M, Pot AM. Effects of combined intervention programmes for people with dementia living at home and their caregivers: a systematic review. International Journal of Geriatric Psychiatry. 2007;22(12):1181-93.

59. Sun Y, Ji M, Leng M, Wang Z. Which cognitive behavioral therapy delivery formats work for depressive symptoms in dementia caregivers? - A systematic review and network meta-analysis of randomized controlled trials. Journal of Affective Disorders. 2022;308:181-7.

60. Tak Y, Song J, Woo H, An J. Realist Review: Understanding Effectiveness of Intervention Programs for Dementia Caregivers. Asian Nursing Research. 2019;13(1):11-9.

61. Thompson CA, Spilsbury K, Hall J, Birks Y, Barnes C, Adamson J. Systematic review of information and support interventions for caregivers of people with dementia. BMC Geriatrics. 2007;7:18.

62. Van't Leven N, Prick AE, Groenewoud JG, Roelofs PD, de Lange J, Pot AM. Dyadic interventions for community-dwelling people with dementia and their family caregivers: a systematic review. International Psychogeriatrics. 2013;25(10):1581-603.

63. Vandepitte S, Van Den Noortgate N, Putman K, Verhaeghe S, Faes K, Annemans L. Effectiveness of Supporting Informal Caregivers of People with Dementia: A Systematic Review of Randomized and Non-Randomized Controlled Trials. J Alzheimer's Dis. 2016;52(3):929-65.

64. Verreault P, Turcotte V, Ouellet MC, Robichaud LA, Hudon C. Efficacy of cognitive-behavioural therapy interventions on reducing burden for caregivers of older adults with a neurocognitive disorder: a systematic review and meta-analysis. Cogn Behav Ther. 2021;50(1):19-46.

65. Wiegelmann H, Speller S, Verhaert LM, Schirra-Weirich L, Wolf-Ostermann K. Psychosocial interventions to support the mental health of informal caregivers of persons living with dementia – a systematic literature review. BMC Geriatrics. 2021;21(1).

66. Williams F, Moghaddam N, Ramsden S, De Boos D. Interventions for reducing levels of burden amongst informal carers of persons with dementia in the community. A systematic review and meta-analysis of randomised controlled trials. Aging and Mental Health. 2019;23(12):1629-42.

67. Wu B, Petrovsky DV, Wang J, Xu H, Zhu Z, McConnell ES, et al. Dementia caregiver interventions in Chinese people: A systematic review. J Adv Nurs. 2019;75(3):528-42.

68. Yorulmaz ED, G. Dementia Caregivers and Cognitive Behavioral Therapy: A Systematic Review. Current Approaches in Psychiatry. 2021;13(2):170-91.

69. Youngran TAK, Junghee S, Haeyoung WOO, Jiyeon AN. Realist Review: Understanding Effectiveness of Intervention Programs for Dementia Caregivers. Asian Nursing Research. 2019:11-9.

70. Zhao Y, Feng H, Hu M, Hu H, Li H, Ning H, et al. Web-Based Interventions to Improve Mental Health in Home Caregivers of People With Dementia: Meta-Analysis. J Med Internet Res. 2019;21(5):e13415.

71. Zientz J, Rackley A, Chapman SB, Hopper T, Mahendra N, Kim ES, et al. Evidence-based practice recommendations for dementia: educating caregivers on Alzheimer's disease and training communication strategies. Journal of Medical Speech-Language Pathology. 2007;15(1):liii-lxiv.

72. M. JJaP. World Alzheimer Report 2009. London, UK: Alzheimer's Disease International; 2009.

73. Cheng ST, Li KK, Losada A, Zhang F, Au A, Thompson LW, et al. The effectiveness of nonpharmacological interventions for informal dementia caregivers: An updated systematic review and meta-analysis. Psychol Aging. 2020;35(1):55-77.

74. González-Fraile E, Ballesteros J, Rueda JR, Santos-Zorrozúa B, Solà I, McCleery J. Remotely delivered information, training and support for informal caregivers of people with dementia. Cochrane Database of Systematic Reviews. 2021;2021(1).

75. Ayoub MF, de Souza YLP, de Almeida T, Falcão D. Synchronous psychological interventions by videoconferencing for caregivers of people with dementia: an integrative review. Dement Neuropsychol. 2022;16(1):1-7.

76. Sun Y, Ji M, Leng M, Li X, Zhang X, Wang Z. Comparative efficacy of 11 non-pharmacological interventions on depression, anxiety, quality of life, and caregiver burden for informal caregivers of people with dementia: A systematic review and network meta-analysis. International Journal of Nursing Studies. 2022;129.

77. Wu KC, Su Y, Chu F, Chen AT, Zaslavsky O. Behavioral Change Factors and Retention in Web-Based Interventions for Informal Caregivers of People Living With Dementia: Scoping Review. Journal of Medical Internet Research. 2022;24(7).

78. DUFOUR N. Dedicated interventions for dementia caregivers by general practitionners : a systematic review

Les interventions dédiées aux aidants familiaux de patients déments par le médecin généraliste (Revue de la littérature) [U - Thesis ; (Thesis)]: Université de Rouen; 2013.

79. Byeon H. Effects of Grief Focused Intervention on the Mental Health of Dementia Caregivers: Systematic Review and Meta-Analysis. Iran J Public Health. 2020;49(12):2275-86.

80. Cheng ST, Au A, Losada A, Thompson LW, Gallagher-Thompson D. Psychological Interventions for Dementia Caregivers: What We Have Achieved, What We Have Learned. Curr Psychiatry Rep. 2019;21(7):59.

81. Christie HL, Bartels SL, Boots LMM, Tange HJ, Verhey FJJ, de Vugt ME. A systematic review on the implementation of eHealth interventions for informal caregivers of people with dementia. Internet Interv. 2018;13:51-9.

82. Christie HL, Martin JL, Connor J, Tange HJ, Verhey FRJ, de Vugt ME, et al. eHealth interventions to support caregivers of people with dementia may be proven effective, but are they implementation-ready? Internet Interv. 2019;18:100260.

83. Cooke DD, McNally L, Mulligan KT, Harrison MJ, Newman SP. Psychosocial interventions for caregivers of people with dementia: a systematic review. Aging Ment Health. 2001;5(2):120-35.

84. Coumoundouros C, Mårtensson E, Ferraris G, Zuidberg JM, von Essen L, Sanderman R, et al. Implementation of e-Mental Health Interventions for Informal Caregivers of Adults With Chronic Diseases: Mixed Methods Systematic Review With a Qualitative Comparative Analysis and Thematic Synthesis. JMIR Ment Health. 2022;9(11):e41891.

85. Sari YM, Hill KD, Lee DCA, Burton E. Effectiveness of exercise programmes in improving physical function and reducing behavioural symptoms of community living older adults with dementia living in Asia, and impact on their informal carers: A systematic review and meta-analysis. Hong Kong Physiother J.15.

86. Gonella S, Mitchell G, Bavelaar L, Conti A, Vanalli M, Basso I, et al. Interventions to support family caregivers of people with advanced dementia at the end of life in nursing homes: A mixed-methods systematic review. Palliat Med. 2022;36(2):268-91.

87. Graven LJ, Glueckauf RL, Regal RA, Merbitz NK, Lustria MLA, James BA. Telehealth Interventions for Family Caregivers of Persons with Chronic Health Conditions: A Systematic Review of Randomized Controlled Trials. Int J Telemed Appl. 2021;2021:3518050.

88. Han A. Effects of Mindfulness-Based Interventions on Depressive Symptoms, Anxiety, Stress, and Quality of Life in Family Caregivers of Persons Living with Dementia: A Systematic Review and Meta-analysis. Res Aging. 2022;44(7-8):494-509.

89. He J, Wang J, Zhong H, Guan C. The Effectiveness of Multi-Component Interventions on the Positive and Negative Aspects of Well-Being among Informal Caregivers of People with Dementia: A Systematic Review and Meta-Analysis. Int J Environ Res Public Health. 2022;19(12).

90. Hui Z, Yang C, Lee DTF. Interventions for Family Members After Long-Term Care Placement of a Relative With Dementia: A Systematic Review and Meta-Analysis. Res Gerontol Nurs. 2021;14(1):43-52.

91. Irons JY, Garip G, Cross AJ, Sheffield D, Bird J. An integrative systematic review of creative arts interventions for older informal caregivers of people with neurological conditions. PLoS ONE. 2020;15(12):e0243461.

92. Klimova B, Valis M, Kuca K, Masopust J. E-learning as valuable caregivers' support for people with dementia - A systematic review. BMC Health Serv Res. 2019;19(1):781.

93. Lee E. Do Technology-Based Support Groups Reduce Care Burden Among Dementia Caregivers? A Review. J Evid Inf Soc Work. 2015;12(5):474-87.

94. Liu X, Wang Y, Wang S. The efficacy of psychological interventions for depressed primary caregivers of patients with Alzheimer's disease: A systematic review and meta-analysis. J Nurs Scholarsh. 2022;54(3):355-66.

95. Meng X, Su J, Li H, Ma D, Zhao Y, Li Y, et al. Effectiveness of caregiver non-pharmacological interventions for behavioural and psychological symptoms of dementia: An updated meta-analysis. Ageing Res Rev. 2021;71:101448.

96. Müller C, Lautenschläger S, Meyer G, Stephan A. Interventions to support people with dementia and their caregivers during the transition from home care to nursing home care: A systematic review. Int J Nurs Stud. 2017;71:139-52.

97. Murfield J, Moyle W, O'Donovan A. Mindfulness- and compassion-based interventions for family carers of older adults: A scoping review. Int J Nurs Stud. 2021;116:103495.

98. Ottaviani AC, Monteiro DQ, Oliveira D, Gratão ACM, Jacinto AF, Campos CRF, et al. Usability and acceptability of internet-based interventions for family carers of people living with dementia: systematic review. Aging Ment Health. 2022;26(10):1922-32.

99. Piersol CV, Canton K, Connor SE, Giller I, Lipman S, Sager S. Effectiveness of Interventions for Caregivers of People With Alzheimer's Disease and Related Major Neurocognitive Disorders: A Systematic Review. Am J Occup Ther. 2017;71(5):7105180020p1-p10.

100. Raj SE, Mackintosh S, Fryer C, Stanley M. Home-Based Occupational Therapy for Adults With Dementia and Their Informal Caregivers: A Systematic Review. Am J Occup Ther. 2021;75(1):7501205060p1-p27.

101. Richardson A, Pedley G, Pelone F, Akhtar F, Chang J, Muleya W, et al. Psychosocial interventions for people with young onset dementia and their carers: a systematic review. Int Psychogeriatr. 2016;28(9):1441-54.

102. Ruggiano N, Brown EL, Roberts L, Framil Suarez CV, Luo Y, Hao Z, et al. Chatbots to Support People With Dementia and Their Caregivers: Systematic Review of Functions and Quality. J Med Internet Res. 2021;23(6):e25006.

103. Saragih ID, Tonapa SI, Lin CJ, Lee BO. Effects of case management intervention for people with dementia and their carers: A systematic review and meta-analysis of experimental studies. Int J Nurs Stud. 2021;121:104012.

104. Saragih ID, Tonapa SI, Porta CM, Lee BO. Effects of telehealth intervention for people with dementia and their carers: A systematic review and meta-analysis of randomized controlled studies. J Nurs Scholarsh. 2022;54(6):704-19.

105. Scott JL, Dawkins S, Quinn MG, Sanderson K, Elliott KE, Stirling C, et al. Caring for the carer: a systematic review of pure technology-based cognitive behavioral therapy (TB-CBT) interventions for dementia carers. Aging Ment Health. 2016;20(8):793-803.

106. Shim M, Tilley JL, Im S, Price K, Gonzalez A. A Systematic Review of Mindfulness-Based Interventions for Patients with Mild Cognitive Impairment or Dementia and Caregivers. J Geriatr Psychiatry Neurol. 2021;34(6):528-54.

107. Sin J, Henderson C, Spain D, Cornelius V, Chen T, Gillard S. eHealth interventions for family carers of people with long term illness: A promising approach? Clin Psychol Rev. 2018;60:109-25.

108. Sitges-Maciá E, Bonete-López B, Sánchez-Cabaco A, Oltra-Cucarella J. Effects of e-Health Training and Social Support Interventions for Informal Caregivers of People with Dementia-A Narrative Review. Int J Environ Res Public Health. 2021;18(15).

109. Van Mierlo LD, Meiland FJ, Van der Roest HG, Dröes RM. Personalised caregiver support: effectiveness of psychosocial interventions in subgroups of caregivers of people with dementia. Int J Geriatr Psychiatry. 2012;27(1):1-14.

110. Waller A, Dilworth S, Mansfield E, Sanson-Fisher R. Computer and telephone delivered interventions to support caregivers of people with dementia: a systematic review of research output and quality. BMC Geriatr. 2017;17(1):265.

111. Wasilewski MB, Stinson JN, Cameron JI. Web-based health interventions for family caregivers of elderly individuals: A Scoping Review. Int J Med Inform. 2017;103:109-38.

112. Ying J, Wang Y, Zhang M, Wang S, Shi Y, Li H, et al. Effect of multicomponent interventions on competence of family caregivers of people with dementia: A systematic review. J Clin Nurs. 2018;27(9-10):1744-58.

113. Yousaf K, Mehmood Z, Saba T, Rehman A, Munshi AM, Alharbey R, et al. Mobile-Health Applications for the Efficient Delivery of Health Care Facility to People with Dementia (PwD) and Support to Their Carers: A Survey. Biomed Res Int. 2019;2019:7151475.

114. Zhu A, Cao W, Zhou Y, Xie A, Cheng Y, Chu SF. Tele-Health Intervention for Carers of Dementia Patients-A Systematic Review and Meta-Analysis of Randomized Controlled Trials. Front Aging Neurosci. 2021;13:612404.

115. Ramachandran M, Bangera K, Anita Dsouza S, Belchior P. A scoping review of family-centered interventions in dementia care. Dementia (London). 2023;22(2):405-38.

116. Wen Y, Xing Y, Ding Y, Xu W, Wang X. Challenges of conducting of online educational programs for family caregivers of people with dementia living at home: An integrative review. Int J Nurs Sci. 2023;10(1):121-8.

117. Yu Y, Xiao L, Ullah S, Meyer C, Wang J, Pot AM, et al. The effectiveness of internet-based psychoeducation programs for caregivers of people living with dementia: a systematic review and meta-analysis. Aging Ment Health. 2023:1-17.

118. Rueda Daz LJ, Monteiro da Cruz DL. The efficacy of telephone use to assist and improve the wellbeing of family caregivers of persons with chronic diseases: a systematic review. JBI Evidence Synthesis. 2014;12(12).

119. Etxeberria I, Salaberria K, Gorostiaga A. Online support for family caregivers of people with dementia: a systematic review and meta-analysis of RCTs and quasi-experimental studies. Aging & Mental Health. 2021;25(7):1165-80.

120. Jackson D, Roberts G, Wu ML, Ford R, Doyle C. A systematic review of the effect of telephone, internet or combined support for carers of people living with Alzheimer’s, vascular or mixed dementia in the community. Archives of Gerontology and Geriatrics. 2016;66:218-36.

121. Teahan Á, Lafferty A, McAuliffe E, Phelan A, O’Sullivan L, O’Shea D, et al. Psychosocial Interventions for Family Carers of People With Dementia: A Systematic Review and Meta-Analysis. Journal of Aging and Health. 2020;32(9):1198-213.

122. Tang WK, Chan CYJ. Effects of psychosocial interventions on self-efficacy of dementia caregivers: a literature review. International Journal of Geriatric Psychiatry. 2016;31(5):475-93.

123. Lucero RJ, Fehlberg EA, Patel AGM, Bjarnardottir RI, Williams R, Lee K, et al. The effects of information and communication technologies on informal caregivers of persons living with dementia: A systematic review. Alzheimer's and Dementia: Translational Research and Clinical Interventions. 2019;5:1-12.

124. Parra-Vidales E, Soto-Pérez F, Victoria Perea-Bartolomé M, Franco-Martín MA, Muñoz-Sánchez JL. Online interventions for caregivers of people with dementia: A systematic review. Actas Esp Psiquiatr. 2017;45(3):116-26.

125. Powell J, Chiu T, Eysenbach G. A systematic review of networked technologies supporting carers of people with dementia. J Telemed Telecare. 2008;14(3):154-6.

126. Ruggiano N, Brown Ellen L, Li J, Scaccianoce M. Rural Dementia Caregivers and Technology: What Is the Evidence? Res Gerontol Nurs. 2018;11(4):216-24.

127. Liu Z, Sun YY, Zhong BL. Mindfulness‐based stress reduction for family carers of people with dementia. Cochrane Database of Systematic Reviews. 2018(8).

128. Sörensen S, Pinquart M, Duberstein P. How effective are interventions with caregivers? An updated meta-analysis. Gerontologist. 2002;42(3):356-72.

129. Walter E, Pinquart M. How Effective Are Dementia Caregiver Interventions? An Updated Comprehensive Meta-Analysis. Gerontologist. 2020;60(8):609-19.

130. Frias CE, Garcia-Pascual M, Montoro M, Ribas N, Risco E, Zabalegui A. Effectiveness of a psychoeducational intervention for caregivers of People With Dementia with regard to burden, anxiety and depression: A systematic review. J Adv Nurs. 2020;76(3):787-802.

131. Hopwood J, Walker N, McDonagh L, Rait G, Walters K, Iliffe S, et al. Internet-Based Interventions Aimed at Supporting Family Caregivers of People With Dementia: Systematic Review. J Med Internet Res. 2018;20(6):e216.

132. Wennberg, Alexandra, Dye, Cheryl, Streetman-Loy, Blaiz, Pham, Hiep. Alzheimer's Patient Familial Caregivers: A Review of Burden and Interventions. Health & Social Work. 2015;40(4):e162-e9.

133. Shim M, Tilley JL, Im S, Price K, Gonzalez A. A Systematic Review of Mindfulness-Based Interventions for Patients with Mild Cognitive Impairment or Dementia and Caregivers. J Geriatr Psychiatry Neurol. 2021;34(6):528-54.

134. Chacko E, Ling B, Avny N, Barak Y, Cullum S, Sundram F, et al. Mindfulness-Based Cognitive Therapy for Stress Reduction in Family Carers of People Living with Dementia: A Systematic Review. International Journal of Environmental Research and Public Health. 2022;19(1):614.

135. Arruda EH, Paun O. Dementia Caregiver Grief and Bereavement: An Integrative Review. West J Nurs Res. 2017;39(6):825-51.

136. Gauthier S WC, Servaes S, Morais JA, Rosa-Neto P. World Alzheimer Report 2022: Life after diagnosis: Navigating treatment, care and support. London, England: Alzheimer's Disease International; 2022.

137. Bui LK, Park M, Giap TTT. eHealth interventions for the informal caregivers of people with dementia: A systematic review of systematic reviews. Geriatric Nursing. 2022;48:199-209.a

**References**

1 Zarit SH, Reever KE, Bach-Peterson J. Relatives of the impaired elderly: correlates of feelings of burden. *The Gerontologist*. 1980;**20**(6):649–55.

2 Novak M, Guest C. Application of a Multidimensional Caregiver Burden Inventory. *The Gerontologist*. 1989;**29**(6):798–803.

3 Kaufer DI, Cummings JL, Christine D, et al. Assessing the impact of neuropsychiatric symptoms in Alzheimer's disease: the Neuropsychiatric Inventory Caregiver Distress Scale. *Journal of the American Geriatrics Society*. 1998;**46**(2):210–5.

4 WHOQOL Group. Development of the World Health Organization WHOQOL-BREF quality of life assessment. *Psychological Medicine*. 1998;**28**(3):551–8.

5 EuroQOL Group. EuroQol--a new facility for the measurement of health-related quality of life. *Health Policy (Amsterdam, Netherlands)*. 1990;**16**(3):199–208.

6 Novelli MM, Nitrini R, Caramelli P. Validation of the Brazilian version of the quality of life scale for patients with Alzheimer's disease and their caregivers (QOL-AD). *Aging & Mental Health*. 2010;**14**(5):624–31.

7 Koçyigit H, Gülseren S, Erol A, Hizli N, Memis A. The reliability and validity of the Turkish version of Quality of Life Questionnaire of the European Foundation for Osteoporosis (QUALEFFO). *Clinical Rheumatology*. 2003;**22**(1):18–23.

8 Montazeri A, Goshtasebi A, Vahdaninia M, Gandek B. The Short Form Health Survey (SF-36): translation and validation study of the Iranian version. *Quality of Life Research: an International Journal of Quality of Life Aspects of Treatment, Care and Rehabilitation*. 2005;**14**(3):875–82.

9 WHOQOL Group. The World Health Organization Quality of Life Assessment (WHOQOL): development and general psychometric properties. *Social Science & Medicine (1982)*. 1998;**46**(12):1569–85.

10 Diener E, Emmons RA, Larsen RJ, Griffin S. The Satisfaction With Life Scale. *Journal of Personality Assessment*. 1985;**49**(1):71–5.

11 Duru Aşiret G, Bagcivan G, Kütmeç Yılmaz C. The Turkish Version of The Carer’s Assessment Of Satisfaction Index (CASI-TR): Its Validation And Reliability. *Türk Fizyoterapi ve Rehabilitasyon Dergisi*. 2019.

12 Goldberg DP, Williams P. A User's Guide to the General Health Questionnaire. Basingstoke: NFER-Nelson; 1988.

13 Mari JJ, Williams P. A comparison of the validity of two psychiatric screening questionnaires (GHQ-12 and SRQ-20) in Brazil, using Relative Operating Characteristic (ROC) analysis. *Psychological Medicine*. 1985;**15**(3):651–9.

14 Dupuy HJ. The general well-being schedule. In: McDowell I, Newell C, editors. A measuring health: A guide to rating scales and questionnaires. New York: Oxford University Press; 1977. p. 125–33.

15 Ryan RM, Frederick C. On energy, personality, and health: subjective vitality as a dynamic reflection of well-being. *Journal of Personality*. 1997;**65**(3):529–65.

16 Walker SN, Hill-Polerecky DM. Psychometric evaluation of the health-promoting lifestyle profile II. *Unpublished manuscript, University of Nebraska Medical Center*. 1996;**13**:120–26.

17 Friborg O, Hjemdal O, Rosenvinge JH, Martinussen M. A new rating scale for adult resilience: what are the central protective resources behind healthy adjustment? *International Journal of Methods in Psychiatric Research*. 2003;**12**(2):65–76.

18 Maneewat T, Lertmaharit S, Tangwongchai S. Development of caregiver resilience scale (CRS) for Thai caregivers of older persons with dementia. *Cogent Medicine*. 2016;**3**(1):1257409.

19 Connor KM, Davidson JR. Development of a new resilience scale: the Connor-Davidson Resilience Scale (CD-RISC). *Depression and Anxiety*. 2003;**18**(2):76–82.

20 Brown KW, Ryan RM. The benefits of being present: mindfulness and its role in psychological well-being. *Journal of Personality and Social Psychology*. 2003;**84**(4):822–48.

21 Neff KD. The Development and Validation of a Scale to Measure Self-Compassion. *Self and Identity*. 2003;**2**(3):223–50.

22 Montazeri A, Vahdaninia M, Mousavi SJ, Asadi-Lari M, Omidvari S, Tavousi M. The 12-item medical outcomes study short form health survey version 2.0 (SF-12v2): a population-based validation study from Tehran, Iran. *Health and Quality of Life Outcomes*. 2011;**9**:12.

23 Beck AT, Epstein N, Brown G, Steer RA. An inventory for measuring clinical anxiety: psychometric properties. *Journal of Consulting and Clinical Psychology*. 1988;**56**(6):893–7.

24 Kvaal K, Ulstein I, Nordhus IH, Engedal K. The Spielberger State-Trait Anxiety Inventory (STAI): the state scale in detecting mental disorders in geriatric patients. *International Journal of Geriatric Psychiatry*. 2005;**20**(7):629–34.

25 Mayzner Jr MS, Sersen E, Tresselt ME. The Taylor Manifest Anxiety Scale and Intelligence. *Journal of Consulting Psychology*. 1955;**19**:401–3.

26 Mostafa F, Ghaly MA. Translation of Taylor's Manifest Anxiety Scale. Cairo: Dar El Maaref; 1962.

27 Henry JD, Crawford JR. The short-form version of the Depression Anxiety Stress Scales (DASS-21): construct validity and normative data in a large non-clinical sample. *The British Journal of Clinical Psychology*. 2005;**44**(Pt 2):227–39.

28 Botega NJ, Bio MR, Zomignani MA, Garcia Jr C, Pereira WAB. Transtornos do humor em enfermaria de clínica médica e validação de escala de medida (HAD) de ansiedade e depressão. *Revista De Saude Publica*. 1995;**29**:359–63.

29 Beck A, Steer R. Brown manual for the beck depression inventory II. *San Antonio, TX: Psychological Corporation*. 1996.

30 Radloff LS. The CES-D Scale:A Self-Report Depression Scale for Research in the General Population. *Applied Psychological Measurement*. 1977;**1**(3):385–401.

31 Hamilton M. Development of a rating scale for primary depressive illness. *The British Journal of Social and Clinical Psychology*. 1967;**6**(4):278–96.

32 Fateem L. Translation of Hamilton depression rating scale. Cairo Al Anglo El Masrya; 1998.

33 Kroenke K, Spitzer RL, Williams JB. The PHQ-9: validity of a brief depression severity measure. *Journal of General Internal Medicine*. 2001;**16**(9):606–13.

34 Kroenke K, Spitzer RL, Williams JB, Löwe B. An ultra-brief screening scale for anxiety and depression: the PHQ-4. *Psychosomatics*. 2009;**50**(6):613–21.

35 Cohen S, Kamarck T, Mermelstein R. A global measure of perceived stress. *Journal of Health and Social Behavior*. 1983;**24**(4):385–96.

36 Robinson BC. Validation of a Caregiver Strain Index. *Journal of Gerontology*. 1983;**38**(3):344–8.

37 Lipp ML. Manual do Invent´ario de Sintomas de Estresse para Adultos de Lipp. S˜ao Paulo, Brazil: Casa do Psic´ologo; 2000.

38 Steffen AM, McKibbin C, Zeiss AM, Gallagher-Thompson D, Bandura A. The revised scale for caregiving self-efficacy: reliability and validity studies. *The Journals of Gerontology Series B, Psychological Sciences and Social Sciences*. 2002;**57**(1):74–86.

39 Gottlieb BH, Rooney JA. Validation of the RIS Eldercare Self-Efficacy Scale. *Canadian Journal on Aging / La Revue canadienne du vieillissement*. 2010;**22**(1):95–107.

40 Pearlin LI, Schooler C. The Structure of Coping. *Journal of Health and Social Behavior*. 1978;**19**(1):2–21.

41 Tran D, Nguyen H, Pham T, et al. Resources for Enhancing Alzheimer's Caregiver Health in Vietnam (REACH VN): Exploratory Analyses of Outcomes of a Cluster Randomized Controlled Trial to Test the Feasibility and Preliminary Efficacy of a Family Dementia Caregiver Intervention in Vietnam. *The American Journal of Geriatric Psychiatry: Official Journal of the American Association for Geriatric Psychiatry*. 2022;**30**(8):878–82.

42 Hinton L, Nguyen H, Nguyen HT, et al. Advancing family dementia caregiver interventions in low- and middle-income countries: A pilot cluster randomized controlled trial of Resources for Advancing Alzheimer's Caregiver Health in Vietnam (REACH VN). *Alzheimer's & Dementia (New York, NY)*. 2021;**6**(1):e12063.

43 Paynter R. 'EPOC LMICs Filter', Cochrane Information Specialist (CIS) Support Digest 2020 [Available from: <https://mailchi.mp/ebb18ffeece7/cochrane-information-specialist-support-team-digest-2864754#EPOC%20LMICs%20Filter> [Accessed 07/08 2021].

44 Dias A, Dewey ME, D'Souza J, et al. The effectiveness of a home care program for supporting caregivers of persons with dementia in developing countries: a randomised controlled trial from Goa, India. *PloS One*. 2008;**3**(6):e2333.

45 Novelli M, Machado SCB, Lima GB, et al. Effects of the Tailored Activity Program in Brazil (TAP-BR) for Persons With Dementia: A Randomized Pilot Trial. *Alzheimer Disease and Associated Disorders*. 2018;**32**(4):339–45.

46 Guerra M, Ferri CP, Fonseca M, Banerjee S, Prince M. Helping carers to care: the 10/66 dementia research group's randomized control trial of a caregiver intervention in Peru. *Revista Brasileira de Psiquiatria (Sao Paulo, Brazil : 1999)*. 2011;**33**(1):47–54.

47 Uyar F, Özmen D, Mavioğlu H, Atalay N. Assessment of the Impact of Dementia Care and Support Program in Both Patient and Caregiver Outcomes: An Intervention Study. *Turkish Journal of Geriatrics-Turk Geriatri Dergisi*. 2019;**22**.

48 Wang LQ, Chien WT, Lee IY. An experimental study on the effectiveness of a mutual support group for family caregivers of a relative with dementia in mainland China. *Contemporary Nurse*. 2012;**40**(2):210–24.

49 Gavrilova SI, Ferri CP, Mikhaylova N, Sokolova O, Banerjee S, Prince M. Helping carers to care--the 10/66 dementia research group's randomized control trial of a caregiver intervention in Russia. *International Journal of Geriatric Psychiatry*. 2009;**24**(4):347–54.

50 Danucalov MA, Kozasa EH, Ribas KT, et al. A yoga and compassion meditation program reduces stress in familial caregivers of Alzheimer's disease patients. *Evidence-Based Complementary and Alternative Medicine : eCAM*. 2013;**2013**.

51 Kamkhagi D, Costa ACO, Kusminsky S, et al. Benefits of psychodynamic group therapy on depression, burden and quality of life of family caregivers to Alzheimer’s disease patients. *Revista De Psiquiatria Clinica*. 2015;**42**:157–60.

52 Söylemez BA, Küçükgüçlü Ö, Buckwalter KC. Application of the Progressively Lowered Stress Threshold Model with Community-Based Caregivers: A Randomized Controlled Trial. *Journal of Gerontological Nursing*. 2016;**42**(7):44–54.

53 Danucalov MA, Kozasa EH, Afonso RF, Galduroz JC, Leite JR. Yoga and compassion meditation program improve quality of life and self-compassion in family caregivers of Alzheimer's disease patients: A randomized controlled trial. *Geriatrics & Gerontology International*. 2017;**17**(1):85–91.
